# Reactions of 3-Hydroxy-2-phenyl-1*H*-benzo[*e*]isoindol-1-one: A Route to 3-Hydroxy-/3-anilinobenzo[*e*]indan-1-ones and Benzo[*f*]phthalazin-1(2*H*)-ones

**DOI:** 10.3390/molecules27238319

**Published:** 2022-11-29

**Authors:** Zbigniew Malinowski, Emilia Fornal, Anna Stachniuk, Monika Nowak

**Affiliations:** 1 Department of Organic Chemistry, Faculty of Chemistry, University of Lodz, Tamka 12, 91-403 Lodz, Poland; 2 Department of Bioanalytics, Medical University of Lublin, Jaczewskiego 8b, 20-090 Lublin, Poland; 3 Department of Immunology and Infectious Biology, Faculty of Biology and Environmental Protection, University of Lodz, 12/16 Banacha, 90-236 Lodz, Poland

**Keywords:** indanones, isoindolinones, Pd cross-coupling, phthalazinones

## Abstract

New hydroxy- and anilinoindanone derivatives **3** and **4** were synthesized starting from 3-hydroxybenzo[*e*]isoindolinone **1** via the addition of alkyllithium (*s*-BuLi, *n*-BuLi, MeLi or *i*-PrLi) to the carbonyl group, followed by lactam ring opening and, finally, an intramolecular cyclization leading to target compounds. The same starting material was used for the preparation of the new benzo[*f*]phthalazinone derivatives **12–16** through multi-step reactions. The target derivative **16** was obtained from the corresponding bromolactam **15** by the Buchwald–Hartwig amination. Structures of the obtained compounds were confirmed by the NMR spectra.

## 1. Introduction

**Figure 1:**
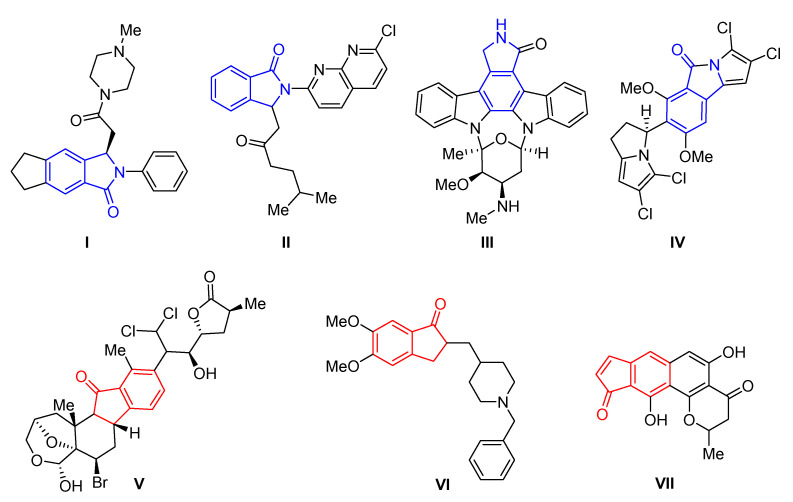
Biologically active isoindolinone and indanone derivatives.

The isoindolinone framework is present as the core unit in many synthetic and naturally occurring derivatives with interesting biological properties [1,2,3]. The ones commonly known are the hypotensive and inhibitory platelet aggregation activity of the nonbenzodiazepine sedative and hypnotic drug JM-1232 (**I**, Figure 1) [4,5,6] as well as the anxiolytic agents Pagoclone (**II**) [7] and Staurosporine (**III**) [8]. Additionally, cytotoxicity activity against the HCT-116 human colon cancer cell line of the naturally occurring Chlorizidine A (**IV**) [9] was found. Cytotoxicity properties also exhibit Nakiterpiosin (**V**) [10,11], a metabolite found in marine sponge (activity toward the P388 murine leukemia cell line, GI_50_ = 10 ng/mL). Moreover, indanone moiety builds the structure of Donepezil (**VI**) [12], a drug useful in the treatment of Alzheimer’s disease (the AChE inhibitor) [13]. Among this active group of compounds, the Euplectin (**VII**) benzo[*f*]indanone derivative with activity against murine mastocytoma cells P-815 (IC_50_ = 1.67 µg/mL) [14] has also found its place.

**Scheme 1:**
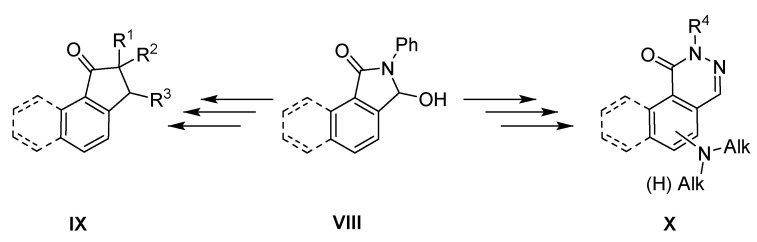
Planned synthetic route.

For a long time, we have been interested in the synthesis and functionalization of substituted isoindolinones [15,16]. In the present work, we focused on 3-hydroxybenzo[*e*]isoindol-1-one **VIII** and the possibilities of its conversion into new carbo- and heterocyclic systems, particularly into derivatives of indan-1-one **IX** and phthalazin-1-one **X** (Scheme 1).

## 2. Results and Discussion

One of the methods of modifying the isoindolinone skeleton is the strategy based on the lithiation of the aromatic ring with alkyllithium, followed by reaction with the electrophile [17,18,19]. Based on this methodology, we wanted to widen the library of substituted benzoisoindolinones, which can be used to prepare new benzo[*f*]phthalazinone derivatives.

The starting 3-hydroxy-2-phenyl-1*H*-benzo[*e*]isoindol-1-one **1** was prepared by treating lithiated *N*-phenylnaphthalene-1-carboxamide (*s*-BuLi, THF, 0 °C) with DMF at 0 °C and, finally, quenching with MeOH. A feature of naphthalene carboxamides is their tendency to undergo an addition reaction of organolithium to the naphthalene ring [20,21]. The increase in the reaction temperature from −78 °C up to 0 °C resulted in a reduction in the amount of formed side products and the formation of the target compound **1** in a 60% yield. Unfortunately, under these conditions, about 10% of the starting amide was recovered.

**Scheme 2:**
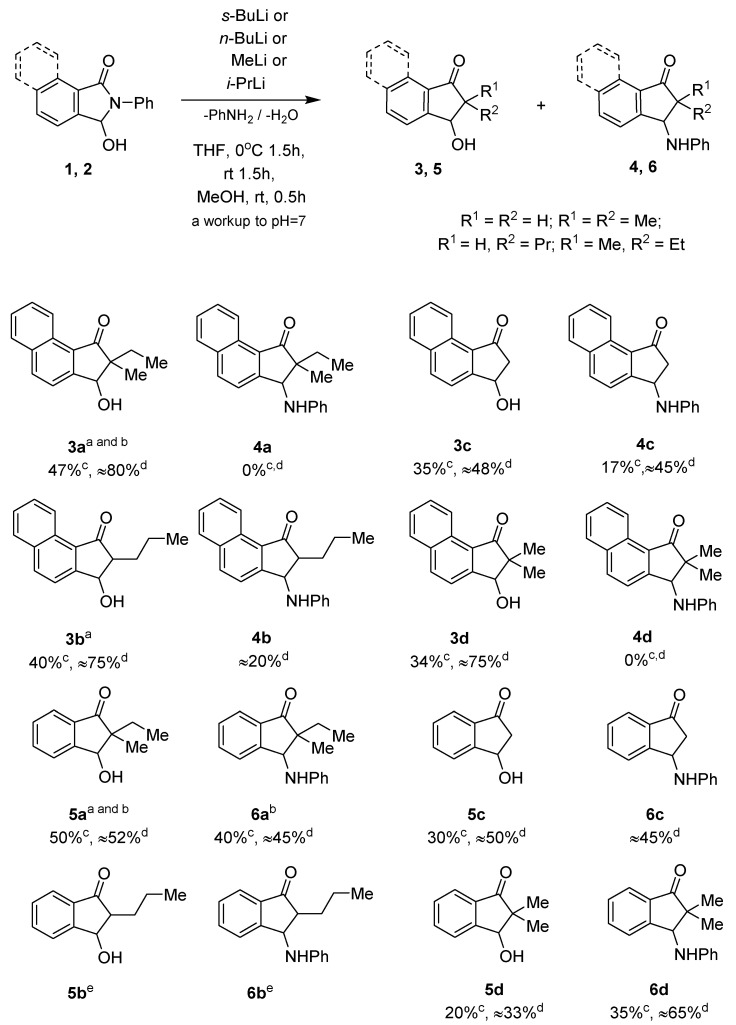
Reaction of isoindolinones **1**, **2** with alkyllithium compounds. ^a^ Isolated as single diastereoisomers; ^b^ isolated as a mixture of diastereoisomers; ^c^ total yield of the isolated product after silica gel chromatography; ^d^ total yield determined by ^1^H NMR analysis of the crude reaction mixture; ^e^ observed in the ^1^H NMR spectrum of the crude reaction mixture.

Unexpectedly, the treatment of benzoisoindolinone **1** with the 2.2 equiv. *s*-BuLi and subsequently with an electrophile, e.g., 1,2-dibromoethane or bromine [22,23,24], led to the formation of 3-hydroxyindanone **3a** as a main product along with a small amount of the starting compound **1** (Scheme 2). Despite repeated attempts, ^1^H NMR analysis of the resulting post-reaction mixtures, after quenching by adding MeOH and a workup to pH ≈ 7, consistently indicated the conversion of **1** into an indanone-type derivative (with and without the addition of an electrophile). Furthermore, ^1^H NMR spectra showed the presence of aniline in the post-reaction mixture, which was due to the formation of keto-alcohol **3a** from **1**.

Additionally, the reactions of **1** with other alkyllithium compounds, under the same reaction conditions as shown in Scheme 2, provided unexpected outcomes. In the case of *i*-PrLi, the course of the reaction with **1** was similar to that of *s*-BuLi, giving 3-hydroxyindanone **3d** as a sole product with a yield of 34%. In turn, the treatment of **1** with *n*-BuLi or MeLi led to the formation of two main products: hydroxy and anilino derivatives: **3b**, **4b** (*n*-BuLi), and **3c**, **4c** (MeLi), respectively, which were observed in the ^1^H NMR spectra of the crude post-reaction mixtures and isolated by flash chromatography. Only in the case of compound **4b** did its separation in its pure form end in failure.

On the other hand, the treatment of isoindolinone **2** with MeLi, *i*-PrLi, *n*-BuLi, or *s*-BuLi each time led to two main types of indanone derivatives, **5** and **6,** in moderate yields (Scheme 2). The structures of compounds **3**–**6** were confirmed with the aid of spectroscopic analyses (for details, see Sections S1.1, S1.2, and S2.3.1 of the Supplementary Materials).

Taking into account our results, the above described, and the recent literature reports on the construction of the indan-1-one skeleton [25], we decided to check how reaction conditions, such as time, temperature, final quenching, and the pH of the post-reaction mixture, might affect the course of the reaction and products’ distribution. For this purpose, we investigated the reaction between isoindolinone **2** and *i*-PrLi (2.2 equiv.) in THF under various conditions (Table 1).

The achieved results, summarized in Table 1, showed that the addition of *i*-PrLi to lactam **2** took place at each temperature. After about 10 min at 0 °C, both indanone derivatives **5d** and **6d** were present in the reaction mixture (^1^H NMR, Table 1, entry 1). Under these conditions, the molar amounts of indanones **5d** and **6d** were similar, although a slight advantage of anilinoindanone **6d** could be found.

Stable molar ratio products, **5d** and **6d**, were achieved by extending the reaction time at 0 °C to 1.5 h (^1^H NMR: **5d**/**6d** 0.47–0.48/1.0) and were not affected by slight changes in temperature or reaction time (Table 1, entries 2, 3, 4).

Lowering the reaction temperature to −78 °C or raising it to rt (Table 1, entries: 7, 8) did not significantly change the distribution of the products. However, when the reaction was carried out at −78 °C, a lot of unreacted **2** was observed.

The best degree of substrate conversion was obtained in the following cases: (1) *i*-PrLi, rt/1.5 h; (2) MeOH, rt/0.5 h (Table 1, entry 7) and (1) *i*-PrLi, 0 °C/1.5 h; (2) rt/1.5 h; (3) MeOH, rt/0.5 h (Table 1, entry 3).

Interestingly, the amount of the formed 3-hydroxyindanone **5d** was reduced by extending the reaction time at rt (Table 1, entries 5, 6). Thus, it seems possible that the initially formed **5d** existed in equilibrium with its open form and could be converted into the anilino derivative **6d**.

**Scheme 3:**
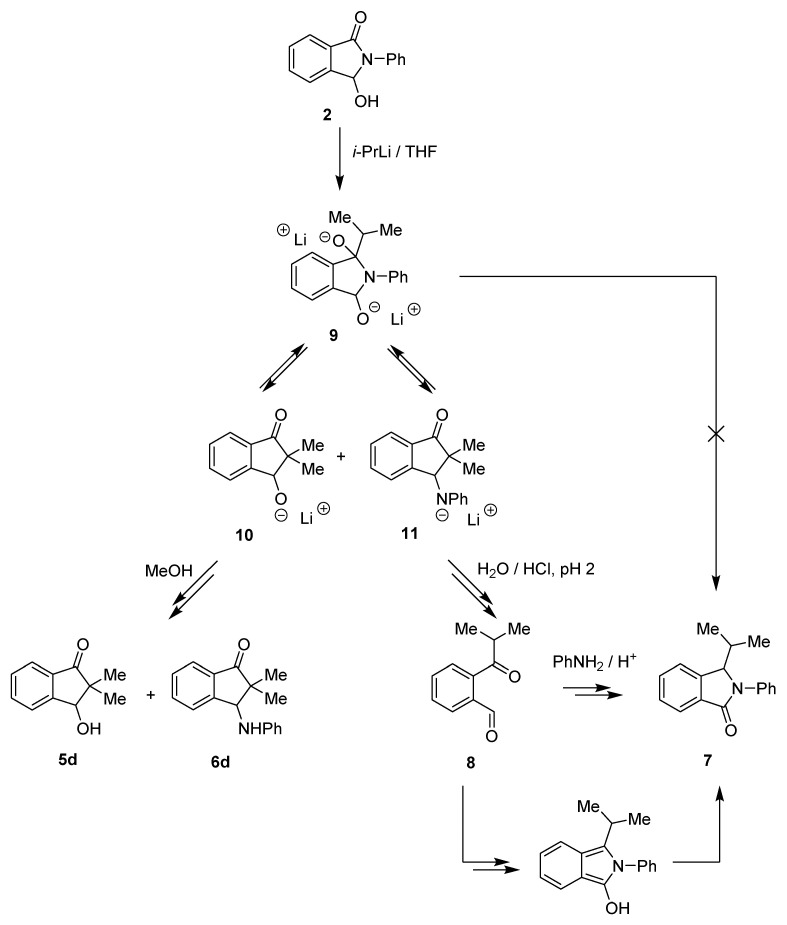
Reaction of isoindolinone **2** with isopropyllithium.

Based on an earlier publication [25], we also expected to obtain 2-phenyl-3-(propan-2-yl)-isoindol-1-one **7** (**2** → **9** → **7**, Scheme 3). Unfortunately, in all our tests (Table 1, entries 1–10 and Scheme 2) we did not observe even a trace of the formation of 3-alkyllactams type **7**.

Therefore, we decided to conduct the experiment under the specific conditions described in the literature [25] (Table 1, entry 11). The reaction was performed without the presence of TMEDA and was quenched with water and acidified to pH ≈ 2. The crude mixture was examined using ^1^H NMR analysis, which showed the characteristic proton signal of the CHO group at 10.05 ppm, corresponding to ketoaldehyde **8** (Table 1). The fractions isolated after chromatographic purification were analyzed one more time using ^1^H NMR. The ^1^H NMR spectrum obtained for the sample of aldehyde **8** was identical to the ones available in the literature [26]. Additionally, an HRMS analysis was performed; thereby, the structure of compound **8** was definitively confirmed.

In our opinion, the final quenching of the reaction had a strong influence on the type of formed products and their distribution. An addition of water followed by acidification (HCl_aq_ 1:1) of the mixture to pH ≈ 2 led to **7** and **8** as main products (the ^1^H NMR spectrum also showed traces of **5d**, **6d,** and **2**). On the other hand, the addition of methanol (without acidification) resulted in the formation of hydroxy- and anilino-indanones **5d** and **6d** without **7** and **8** (Table 1, Scheme 3).

The presented results suggested that isoindolinone **7** probably did not arise directly from 3-hydroxyisoinolinone **2** (via **9**), as was proposed in [25]; but its formation may be the effect of the specific conditions under which the reaction was finalized and the condensation of aldehyde **8** (formed, e.g., from hydroxyindanone **5d** by the retro aldol reaction) with aniline in the presence of acidic catalysts [27] (Scheme 3).

Presumably, the reaction between 3-hydroxyisoindolinones **1** and **2** and alkyllithiums can have several paths, and the choice of each of them is determined by the specific properties of the substrates and reaction conditions. At present, we have not discussed the detailed mechanism of the described reactions because that would be premature.

**Scheme 4:**
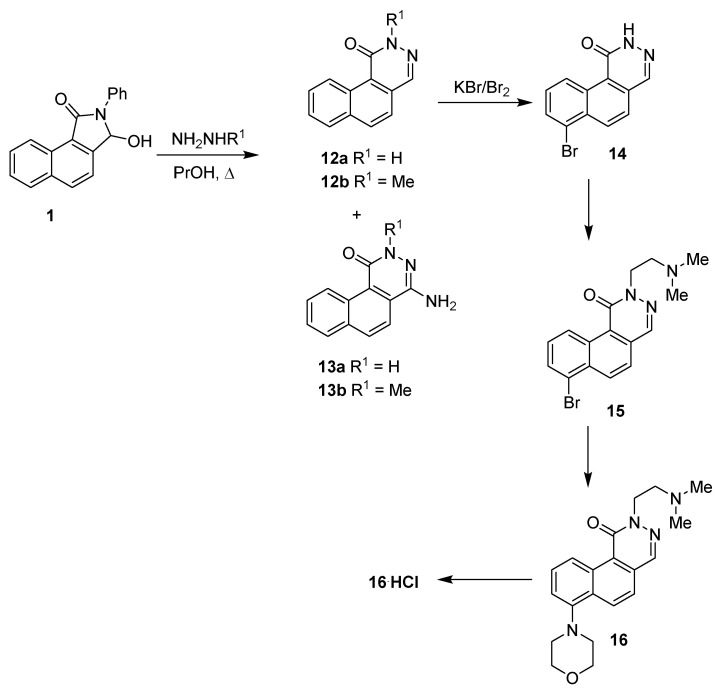
Synthesis of benzo[*f*]phthalazinone derivatives.

The second part of our research focused on the transformation of the benzo[*e*]isoindolinone skeleton into benzo[*f*]phthalazinone derivatives and their subsequent modifications. In the first stage, we conducted the heterocyclic ring expansion of **1** to benzo[*f*]phthalazinone **12** using hydrazine hydrates or methylhydrazine (Scheme 4) [28].

In general, the reaction between various substituted 3-hydroxyisoindolinones and hydrazine leads to the formation of phthalazinones, as sole products, with good yields [15,16]. Unexpectedly, the treatment of benzo[*e*]isoindolinone **1** with hydrazine hydrate resulted in the formation of benzo[*f*]phthalazinone **12a** (29%) and 4-aminobenzo[*f*]phthalazinone **13a** (25%) side by side.

A similar result was also achieved under the reaction of lactam **1** with methylhydrazine. Additionally, *N*-methylphthalazinone **12b** (24%), the 4-amino- derivative **13b** (9%), was isolated from the reaction mixture, too. The separation and purification process of both phthalazinone derivatives **12** and **13** turned out to be problematic due to their limited solubility.

The structures of derivatives **12** and **13** were characterized based on a comparison of the ^1^H NMR and ^13^C NMR spectra. The diagnostic value in the identification of phthalazinone derivatives had chemical shifts of the methine proton (N=CH) and NH_2_ protons of the amine substituent attached to carbon C-4. In the case of **12a,** the C4-H proton signal was appreciably more shifted downfield (singlet at 8.51 ppm) than in the instance of the *N*-methyl derivative **12b** (singlet at 8.24 ppm), while the protons of the NH_2_ groups of **13a**,**b** were present as singlets in the range of 6.01–6.12 ppm.

**Scheme 5:**
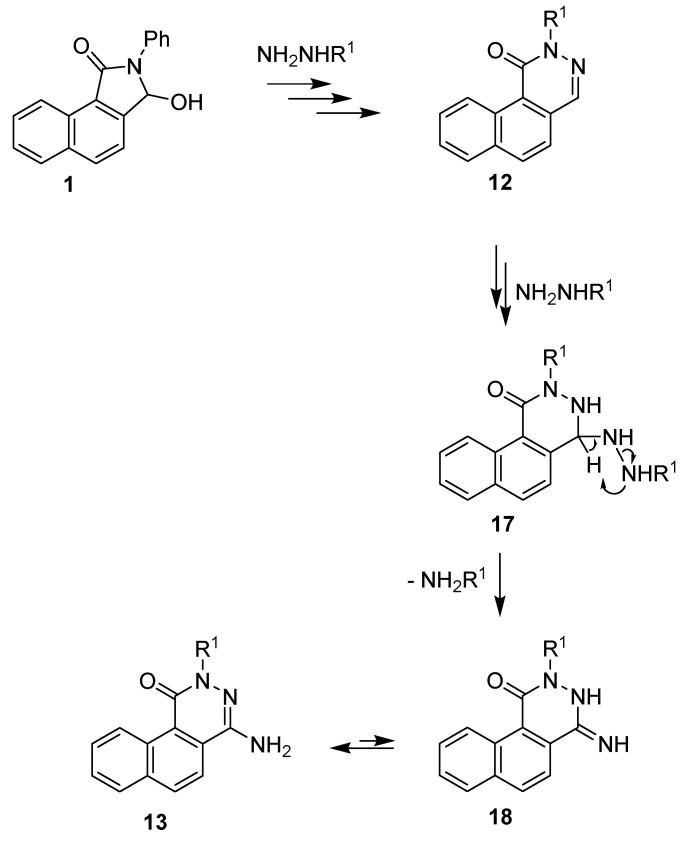
Proposed method of formation of 4-aminophthalazinones **13**.

In our opinion, the observed formation of aminophthalazinones **13** was a result of the reaction between benzo[*f*]phthalazinone **12** and excess hydrazine (methylhydrazine). The proposed mechanism for the formation of 4-aminophthalazinones is presented in Scheme 5. The key step in the formation of **13** was the addition of hydrazine (methylhydrazine) to the azomethine bond of lactams **12** (**12** → **17**), followed by the N–N bond cleavage of hydrazine derivatives **17** and, finally, the elimination of ammonia (methylamine), leading to the amine-imine tautomeric system **13**⇆**18**. Interestingly, the reaction did not occur when a stoichiometric amount of hydrazine was used.

The usual methods used for the cleavage of the N–N bonds in hydrazine derivatives include, among other things, reduction with Zn/H^+^, SmI_2_, and Na/NH_3_; catalytic hydrogenolysis by Ni-RANEY; PtO_2_ and oxidative cleavage with *meta*-chloroperbenzoic acid; etc. [29]. Some years ago, the synthesis of amines starting from substituted hydrazines and naphthols only under thermal conditions was also described [30].

In our recent works, we have shown the possibilities of phthalazinone skeleton modifications via the Pd cross-coupling reaction of *N*-substituted 4-bromophthalazinones with selected amines and thiols (Buchwald–Hartwig-type coupling) [28,31]. Therefore, we first started with the conversion of lactam **12a** into bromoderivative in a Br_2_-KBr acetate buffer solution. However, attempts to transform **12a** into 4-bromoderivative ended in failure. It turned out that the introduction of the additional benzene ring, condensed with phthalazinone moiety, significantly changed the reactivity of the molecule, whereby **12a** (Scheme 4) underwent bromination (KBr-Br_2_) [28] in the 7 position, leading to derivative **14**, with a yield of 87%. In the next stage, compound **14**, without an additional purification process, was treated with 2-chloroethyldimethylamine hydrochloride in the presence of MeONa in methanol, delivering the disubstituted benzo[*f*]phthalazinone **15** at an overall yield of 52% (**12a** → **14** → **15**).

The position of the bromine atom in **14** and **15** was confirmed by 1D and 2D NMR (COSY, HMQC, HMBC) spectroscopy and additionally, by comparison with literature data [32] (for details, see Sections S1.3 and S2.4.2 of the Supplementary Materials).

The palladium cross-coupling reaction between bromolactam **15** and morpholine in the presence of the Pd/*R*-BINAP catalytic system was performed according to the methodology described by us recently [28]. This method led to product **16** in a 44% yield.

Unexpectedly, the 1D and 2D NMR spectroscopy studies of diamine **16** were complicated by the use of an unstable CDCl_3_, which partially decomposed during storage to form HCl [33,34,35,36,37,38]. This unplanned complication resulted in the formation of hydrochloride salt of diamine **16** (Scheme 4, compound **16**•**HCl**) and prompted us to look more closely at the ^1^H and ^13^C NMR spectra of **16**•**HCl**.

Interestingly, the ^1^H and ^13^C NMR spectroscopy of amine salts, especially active substances, in solvents such as CDCl_3_ or DMSO-*d_6_*, is a useful research tool for their analysis [35,36].

When comparing the ^1^H NMR spectra of diamine **16** and salt **16**•**HCl**, attention is drawn to the widened singlet at 12.97 ppm, which is specific for the NH^+^ proton [35,38].

The difference of electronegativity between protonated and neutral nitrogen atoms causes chemical shifts of protons of methylene and methyl groups in the vicinity of NH^+,^ which are shifted downfield (Table S1, Supplementary Materials) [35,36].

It is known from the literature [39] that typical ^1^H chemical shift values for the *N*,*N*-dimethylaminoethyl moiety of 2-substituted phthalazinone derivatives fall in the ranges of 4.18–4.63 ppm and 2.75–2.88 ppm for protons of two CH_2_ groups and 2.26–2.37 ppm for protons of Me groups. The same is the case with diamine **16**.

The protons of the two methyl groups (NH^+^Me_2_) in the spectrum of **16**•**HCl** were seen as a six-proton doublet at 2.93 ppm; for diamine **16,** they were shown as a singlet at 2.37 ppm. In the case of the protons of ethylene moiety, the greatest changes in chemical shifts and multiplicity were visible for two protons of the methylene group closest to the protonated nitrogen atom. Their resonance signal was shifted downfield by 0.76 ppm in comparison to **16** (2.85 ppm).

On the other hand, the resonance signals of the protons of morpholin-4-yl moiety did not change their position and multiplicity (Table S1, Supplementary Materials), which may indicate that only the nitrogen atom of the NMe_2_ group was protonated. The absorptions of aromatic protons were slightly shifted downfield, without the C10-H.

Additionally, ^1^H and ^13^C NMR data analysis was performed for 2-[2-(dimethylamino)ethyl]phthalazin-1(2*H*)-one **19** [39] and its HCl salt (**19**•**HCl**, generated in situ in chloroform solution) (for details, see Sections S1.4 and S2.4.4 of the Supplementary Materials).

Based on the ^13^C chemical shift values of **19**, its derivative **19**•**HCl,** and also 2D NMR data (^1^H,^13^C HMQC), it was possible to assign resonance signals in the ^13^C NMR spectrum of **16**•**HCl**. In the range of 118 to 161 ppm, all signals of the aromatic carbons, C=O (160.7 ppm), and N=C (138.6 ppm) groups were visible. Similarly, the signals of carbons of the morpholin-4-yl substituent were displayed at 67.5 and 54.0 ppm. The carbon resonance pics of the ethylene and methyl groups were of low intensity and often difficult to observe; finally, they were assigned from the ^1^H,^13^C HMQC spectrum. The signals appearing at 56.0 and 47.7 ppm were attributed to *C*H_2_NH^+^ and C(O)N*C*H_2_, respectively. In turn, the signal of carbon methyl groups appeared at 44.1 ppm.

## 3. Materials and Methods

### 3.1. General Information

Melting points were determined on a Boetius hot-stage apparatus and were uncorrected. ^1^H and ^13^C NMR spectra were recorded on a Bruker Advance III spectrometer at 600 MHz and 150 MHz, respectively. Chemical shifts (δ_H_, δ_C_) were quoted in parts per million (ppm), referenced to the signal of TMS or to the appropriate residual solvent peak (CDCl_3_ at 7.26 ppm or DMSO-*d_6_* at 2.50 ppm for ^1^H NMR; CDCl_3_ at 77.16 ppm or DMSO-*d_6_* at 39.52 ppm for ^13^C NMR) [40]. Coupling constants (*J*) were quoted in Hertz (Hz). The 2D homonuclear ^1^H and ^1^H COSY and heteronuclear ^1^H, ^13^C HMQC, and HMBC spectra were used to assign the proton and carbon signals. IR spectra were recorded on a Nexus FT-IR spectrometer.

LC/HRMS analyses were performed using an Agilent Technologies HPLC 1290 coupled to an Agilent Technologies 6550 Accurate Mass Q-TOF LC-MS mass spectrometer equipped with a JetStream Technology ion source housed in the Department of Pathophysiology, Medical University of Lublin, Poland. Internal mass calibration was enabled; reference ions of *m*/*z* = 121.0509 and 922.0098 were used.

The analytical thin layer chromatography tests (TLC) were carried out on Sigma-Aldrich (Supelco) silica gel plates (Kiselgel 60 F254, layer thickness 0.2 mm), and the spots were visualized using a UV lamp. The flash column chromatography purifications were performed on Fluka silica gel (Silica gel 60, 0.040–0.063 mm).

Alkyllithium solutions (*s*-butyllithium in cyclohexane (*s*-BuLi), *n*-butyllithium in hexanes (*n*-BuLi), methyllithium in diethyl ether (MeLi), isopropyllithium in pentane (*i*-PrLi); Sigma-Aldrich) were each time titrated before use [41].

All reactions with organolithium and organopalladium compounds were performed under an argon atmosphere using the standard Schlenk technique. THF and toluene were distilled from sodium benzophenone ketyl prior to use.

Commercially available reagents (hydrazine monohydrate, methylhydrazine, 2-chloro-*N*,*N*-dimethylethylamine hydrochloride, *t*-BuOK, tris(dibenzylideneacetone)-dipalladium(0) (Pd_2_(dba)_3_), (*R*)-2,2′-bis(diphenylphosphino)-1,1′-binaphthalene (*R*-BINAP)) were purchased from Sigma-Aldrich and were used without further purification.

*N*-Phenylnaphthalene-1-carboxamide [42,43,44], 3-hydroxy-2-phenyl-2,3-dihydro-1*H*-isoindol-1-one **2** [45], and 2-[2-(dimethylamino)ethyl]-2*H*-phthalazin-1-one **19** [39] were obtained as described previously; their characterization data were in agreement with the reported analysis. The characterization data of 2-(2-methylpropanoyl) benzaldehyde **8** [26] and 3-hydroxy-2,3-dihydro-1*H*-inden-1-one **5c** [46] were in agreement with the already reported analysis.

### 3.2. Synthesis of 3-Hydroxy-2-phenyl-2,3-dihydro-1H-benzo[e]isoindol-1-one (1)

To the stirred at 0 °C under argon solution of *N*-phenylnaphthalene-1-carboxamide (4.04 × 10^−3^ mol) in THF (30 mL), *n*-BuLi in hexanes (8.89 × 10^−3^ mol) was added dropwise. The solution was held at 0 °C for 1 h. Then, DMF (2.68 × 10^−2^ mol) was added dropwise. The reaction mixture after 1 h at 0 °C was warmed to rt and stirred under these conditions for 2 h. After that, methanol (25 mL) was added, and stirring was continued for 0.5 h. Then, solvents were removed under reduced pressure. Water (25 mL) and DCM (25 mL) were added to the obtained residue, and the whole lot was neutralized with HCl_aq_ (1:1, *v*/*v*). The water layer was separated and extracted subsequently with DCM (3 × 15 mL). The organic phase was dried over MgSO_4_ and concentrated until dryness. The crude material was separated by flash chromatography (Hex-AcOEt 4:1, 3:1, 2:1).

### 3.3. Synthesis of 3-Hydroxy-2,3-dihydro-1H-inden-1-one 3 and 5 and 3-Anilino-2,3-dihydro-1H-inden-1-one 4 and 6 Derivatives

Under argon, to the solution of 3-hydroxyisoindolinone **1** or **2** (1.33 × 10^−3^ mol) in THF (20 mL) at 0 °C, the solution of alkyllithium compound (*s*-BuLi, *n*-BuLi, MeLi, or *i*-PrLi, 2.93 × 10^−3^ mol) was added dropwise. The solution was stirred at 0 °C for 1.5 h. Next, the reaction mixture was warmed to rt and stirred under these conditions for 1.5 h. After that, methanol (15 mL) was added and stirring was continued for 0.5 h. Then, solvents were removed under reduced pressure. Water (40 mL) and DCM (40 mL) were added to the obtained residue, and the whole lot was neutralized with HCl_aq_ (1:1, *v*/*v*). The water layer was separated and extracted subsequently with DCM (3 × 15 mL). The organic phase was dried over MgSO_4_ and concentrated until dryness. The crude material was separated by flash chromatography.

### 3.4. Synthesis of benzo[f]phthalazin-1(2H)-ones ***12*** and 4-amino-benzo[f]phthalazin-1(2H)-ones ***13***

Typical procedure [28].

A mixture of 3-hydroxyisoindolinone **1** (2.56 × 10^−3^ mol) and hydrazine monohydrate (3 mL) in propan-1-ol (15 mL) was heated with stirring under reflux for 38 h. After the completion of the reaction (TLC check), the mixture was cooled, and all volatile materials were removed under reduced pressure. To the residue, water was added and neutralized with acetic acid. The separated solid was collected by filtration, washed with water, dried by vacuum suction, and subjected to flash chromatography to obtain products **12** and **13**.

### 3.5. Synthesis of 7-bromo-2-[2-(dimethylamino)ethyl]benzo[f]phthalazin-1(2H)-one *(**15**)*

Step 1. Bromination of benzo[*f*]phthalazinone **12a** (analogous procedure as described in [28]).

To a suspension of benzophthalazinone **12a** (2.45 × 10^−4^ mol) in the acetate buffer solution pH ≈ 5.8 (5 mL) was added potassium bromide (2.69 × 10^−4^ mol) and bromine (2.69 × 10^−4^ mol) at an ambient temperature. The mixture was then stirred for 6 h at an ambient temperature; next, the whole was heated to boiling and stirred for 30 h until the bromine color entirely disappeared. After cooling to an ambient temperature, the separated solid was collected by filtration and washed with water (2 mL). The crude product **14** (Scheme 4, yield: 58 mg, 87%) was used in the next step without additional purification.

Step 2. Alkylation of 7-bromobenzo[*f*]phthalazinone **14** (analogous procedure as described in [38]).

To the solution of sodium methoxide (6.32 × 10^−4^ mol) in dry methanol (15 mL) was added bromophthalazinone **14** (2.11 × 10^−4^ mol) and (2-chloroethyl)dimethyl ammonium chloride (4.22 × 10^−4^ mol). The mixture was heated at reflux for 30 h. Afterwards, the inorganic material was collected by filtration and washed with dry methanol; the filtrate was evaporated to dryness. The residue was subjected to flash chromatography (MeOH-CHCl_3_ 1:1) to give the pure product **15**.

### 3.6. Synthesis of 2-[2-(dimethylamino)ethyl]-7-(morpholin-4-yl)benzo[f]phthalazin-1(2H)-one *(**16**)*

Analogous procedure as described in [31].

The reaction was carried out under an argon atmosphere in an oven-dried resealable Schlenk flask. A resealable Schlenk flask was charged with Pd_2_(dba)_3_ (1 mol%), (*R*)-BINAP (15 mol%), and freshly distilled toluene (5 mL). The contents of the flask were stirred for 1 min. Then, morpholine (2.4 equiv.) was added, and the whole was again stirred and heated at ≈100 °C for 20 min. After this time, the reaction was cooled. Next, bromophthalazinone **15** (5.78 × 10^−5^ mol), *t*-BuOK (1.2 equiv.), and toluene (2 mL) were added. This mixture was then stirred and heated at ≈100 °C for 20 h. After this time, it was cooled and diluted with chloroform (2 mL). The solid was filtered off and washed with chloroform (2 mL), and the filtrate was concentrated. The product was purified by flash chromatography.

## 4. Conclusions

This article considers the possibilities for the use of the 3-hydroxybenzoisoindol-1-one skeleton to form new indan-1-one or phthalazin-1-one derivatives. We performed a preliminary analysis of synthetic pathways for the reaction of isoindolinones **1** and **2** with selected organolithium compounds. The predicted mechanism without details of the noted products’ formation was discussed. As confirmed by the ^1^H NMR spectra, in the case of naphthalene derivatives **1**, the main products of the mentioned transformations were hydroxyindanones. In turn, after the reaction of isoindolinone **2** with organolithium compounds, in the ^1^H NMR spectra of crude reaction mixtures, two potential products were visible, amino- and hydroxyindanones, present in various ratios. The course of this process was not entirely clear, but our observations can suggest the existence of some specific equilibrium between both compounds.

Furthermore, the transformation of the benzoisoindolinone **1** skeleton to benzophthalazinone **12** or **13** derivatives was described. In the synthesis of these compounds, the key role was played by hydrazine, which was used in excess. Research also showed that Pd coupling of bromobenzophthalazinone **15** with morpholine can be performed by applying a Pd/*R*-BINAP catalytic system according to the method previously described by us.

It was concluded that this study has opened up the highly interesting option for the transformation of lactams **1** and **2** into cyclic hydroxy- and amino-ketones **3**–**6** and phthalazinones **12**–**16** with huge synthetic potential.

## Figures and Tables

**Table 1:**
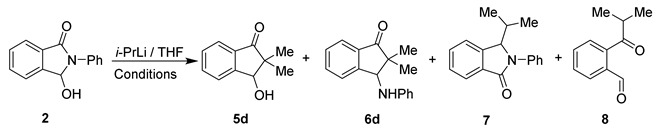
Effect of reaction conditions on products’ distribution.

Entry	Conditions	Molar Ratio of Products ^b^				
	Temperature/Time	2	5d	6d	7	8
1	(1) *i*-PrLi 0 °C and 10 min at 0 °C; (2) MeOH 0 °C, 10 min at 0 °C. ^a^	0.73	0.82	1.00	- ^c^	- ^c^
2	(1) *i*-PrLi 0 °C and 1.5 h at 0 °C; (2) MeOH 0 °C, 0.5 h at 0 °C. ^a^	0.31	0.48	1.00	- ^c^	- ^c^
3	(1) *i*-PrLi 0 °C and 1.5 h at 0 °C; (2) 0 °C → rt, 1,5 h at rt; (3) MeOH rt, 0.5 h at rt. ^a^	Trace	0.47	1.00	- ^c^	- ^c^
4	(1) *i*-PrLi 0 °C and 1.5 h 0 °C; (2) 0 °C → rt, 4 h at rt; (3) MeOH rt, 0.5 h at rt. ^a^	0.23	0.48	1.00	- ^c^	- ^c^
5	(1) *i*-PrLi 0 °C and 1.5 h at 0 °C; (2) 0 °C → rt, 20 h at rt; (3) MeOH rt, 0.5 h at rt. ^a^	0.18	0.10	1.00	- ^c^	- ^c^
6	(1) *i*-PrLi 0 °C and 1.5 h at 0 °C; (2) 0°C → rt, 36 h at rt; (3) MeOH rt, 0.5 h at rt. ^a^	0.21	0.10	1.00	- ^c^	- ^c^
7	(1) *i*-PrLi rt and 1.5 h at rt; (2) MeOH rt, 0.5 h at rt. ^a^	0.04	0.52	1.00	- ^c^	- ^c^
8	(1) *i*-PrLi −78 °C and 1.5 h at −78 °C; (2) MeOH, −78 °C, 0.5 h at −78 °C. ^a^	1.01	0.47	1.00	- ^c^	- ^c^
9	(1) *i*-PrLi 0 °C and 1.5 h at 0 °C; (2) 0 °C → rt, 0.5 h at rt; (3) MeOH rt, 0.5 h at rt. ^a^	0.18	0.56	1.00	- ^c^	- ^c^
10	(1) *i*-PrLi 0 °C and 1.5 h at 0 °C; (2) 0 °C → rt, 1,5 h at rt; (3) H_2_O rt, 0.5 h at rt, next to pH ≈ 7 (HClaq, 1:1, *v*/*v*).	1.30	0.29	1.00	- ^c^	- ^c^
11	(1) *i*-PrLi −78 °C and 1.5 h at −78 °C; (2) −78 °C → rt, 0.5 h at rt; (3) H_2_O rt, 0.5 h at rt, next to pH ≈ 2 (HClaq, 1:1, *v*/*v*).	0.85	Trace	Trace	0.35	1

^a^ To dryness, next DCM–H_2_O to pH ≈ 7 (HCl_aq_, 1:1, *v*/*v*); ^b^ the molar ratio was determined based on ^1^H NMR analysis of the crude reaction mixture; ^c^ the product was not observed in the ^1^H NMR spectrum of the crude reaction mixture.

## Data Availability

Not applicable.

## Supplementary Materials

The following supporting information can be downloaded at https://www.mdpi.com/article/10.3390/molecules27238319/s1. Table S1, ^1^H NMR chemical shifts of **16** and **16**•**HCl;** Table S2, ^1^H NMR chemical shifts of **19** and **19**•**HCl;** Table S3, ^13^C NMR chemical shifts of **19** and **19**•**HCl;** Figure S1**,** The coupling constants between protons in naphthalene moiety of benzophthalazinones **14**, **15** and benzoquinazolinone **20;** Figures S2–S76**,** ^1^H, ^13^C, 2D NMR spectra of compounds **1, 3–8, 12–16**•**HCl** and **19**•**HCl**.
