# Supplementary material for: Reactions of 3-Hydroxy-2-phenyl-1H-benzo[e]isoindol-1-one: A Route to 3-Hydroxy-/3-anilinobenzo[e]indan-1-ones and Benzo[f]phthalazin-1(2H)-ones
Source: Molecules. 2022 Nov 29;27(23):8319. doi: 10.3390/molecules27238319 (PMC9737834; doi:10.3390/molecules27238319)
Supplement: Supplementary file 1 [file molecules-27-08319-s001.zip › molecules-2010137-supplementary.pdf]

Supporting Information

For

Reactions of 3-Hydroxy-2-phenyl-1*H*-benzo[*e*]isoindol-1-one: A Route to 3-Hydroxy-/3-Anilinobenzo[*e*]indan-1-ones and Benzo[*f*]phthalazin-1(2*H*)-ones

Zbigniew Malinowski*[1], Emilia Fornal[2], Anna Stachniuk[2], Monika Nowak[3]

Table of Contents

**S1.**	**Theoretical**	**S2**
**S1.1.**	NMR data analysis of 3-hydroxyindanones **3, 5**	**S2**
**S1.2.**	NMR data analysis of 3-anilinoindanones **4**, **6**	**S2**
**S1.3.**	The position of bromine substituent in **14**, **15**	**S2**
**S1.4.**	NMR data analysis of of 2-[2-(dimethylamino)ethyl]phthalazin-1(2*H*)-one (**19**) and **19•HCl**	**S3**
**S2.**	**Experimental**	**S5**
**S2.1.**	General information	**S5**
**S2.2.**	Synthesis of 3-hydroxy-1*H*-isoindol-1-ones **1**	**S6**
**S2.3.**	Reactions of 3-hydroxy-1*H*-benzo[*e*]isoindol-1-one **1** and 3-hydroxy-1*H*-isoindol-1-one **2** with organolithium compounds: *s*-BuLi, *n*-BuLi, MeLi and *i*-PrLi	**S6**
**S2.3.1.**	Synthesis of 3-hydroxy-2,3-dihydro-1*H*-inden-1-one **3**, **5** and 3-anilino-2,3-dihydro-1*H*--inden-1-one derivatives **4**, **6**	**S6**
**S2.3.2.**	Synthesis of 2-phenyl-3-(propan-2-yl)-2,3-dihydro-1*H*-isoindol-1-one (**7**) and 2-(2-methylpropanoyl)benzaldehyde (**8**)	**S9**
**S2.4.**	Synthesis of benzo[*f*]phthalazin-1(2*H*)-one derivatives **12-16**	**S10**
**S2.4.1.**	Synthesis of benzo[f]phthalazin-1(2*H*)-ones **12** and 4-amino-benzo[*f*]phthalazin-1(2*H*)-ones **13**	**S10**
**S2.4.2.**	Synthesis of 7-bromo-2-[2-(dimethylamino)ethyl]benzo[*f*]phthalazine-1(2*H*)-one (**15**)	**S10**
**S2.4.3.**	Synthesis of 2-[2-(dimethylamino)ethyl]-7-(morpholin-4-yl)benzo[*f*]phthalazin-1(2*H*)-one (**16**)	**S11**
**S2.4.4.**	Hydrochlorides **16•HCl** and **19•HCl**	**S11**
**S2.5.**	^{1}H, ^{13}C, 2D NMR spectra of compounds **1**, **3-8**, **12-16•HCl** and **19•HCl**	**S13**

S1. **Theoretical**

S1.1. NMR data analysis of 3-hydroxyindanones **3**, **5**.

The diagnostic value in the identification of hydroxyindanones **3** and **5** had the chemical shift of the proton bonded to carbon C3 (H3 proton). The C3-H proton signals were observed in a range of 4.96-5.46 ppm as a doublet or doublet of doublets, as a result of coupling with the C2-H proton(s) and with the OH proton – for **3a**, **3b**). In turn, signals situated between 1.91 and 2.53 ppm belong to protons from OH groups. Additionally, the presence of OH proton was confirmed by exchange with D_2O (**3b**,**c**,**d**) or the HMQC analysis (**3c**,**d**, **5d**).

Furthermore, in the ^{13}C NMR spectra of **3**, **5** signals corresponding to the carbons of C_{sp2}-O and C_{sp3}-O moiety were observed in a range of 203.5-208.8 ppm and 68.3-78.7 ppm, respectively. Whereas for the carbon atom C2 at a range of 47.8-58.6 ppm.

The presence of alkyl groups attached to the C2 carbon had a pronounced influence on its chemical shift and caused a downfield shift of the signal. This tendency was visible, e.g., in the case of derivative **3c** (without alkyl substituents at C2) and **3b** (-Pr at C2) for which carbon signals were situated at 47.8 and 58.6 ppm, respectively. A similar effect can be seen in the case of the shift of C3 carbons. For **3c** the resonance of the C3 was located at 68.3 ppm, whereas for **3b** it was observed at 74.8 ppm.

The NMR spectra of **3**, **5** were assigned with the aid of 2D NMR experiments (COSY, HMQC, HMBC).

S1.2. NMR data analysis of 3-anilinoindanones **4**, **6**.

In the case of aminoindanones **4c**, **6a,d**, the C3-H proton signal was observed in a range of 4.98-5.27 ppm ($CDCl_3$) as a doublet or doublet of doublets, - a coupling with C2-H protons for **4c** or with the NH proton – for **6a**, **6d**. In turn, signals located between 3.88 and 4.05 ppm ($CDCl_3$) belong to proton from the NH (singlet for **4c** and doublets for **6a**,**d** ($J \approx$ 9.7-10.0 Hz). Similarly as in the cases of hydroxyindanones, the presence of the NH proton in **4**, **6** was confirmed by exchange with D_2O (**6a**,**d**) or the HMQC analysis (**4c**, **6a**,**d**). In the case of compound **6a**, the solvent change from $CDCl_3$ to DMSO-d_6 resulted in a downfield shift of the NH resonance signal from 3.88 to 5.97 ppm for **6a'** and from 4.01 to 6.06 ppm for **6a"**.

In the ^{13}C NMR spectra signals corresponding to the carbons of C=O group were observed in a range of 204.2-209.0 ppm. Whereas for carbon atoms C3 and C2 at a range 51.6-62.6 ppm and 46.1-58.5 ppm, respectively. The NMR spectra of **4**, **6** were assigned with the aid of 2D NMR experiments (COSY, HMQC, HMBC).

S1.3. The position of bromine substituent in **14**, **15**.

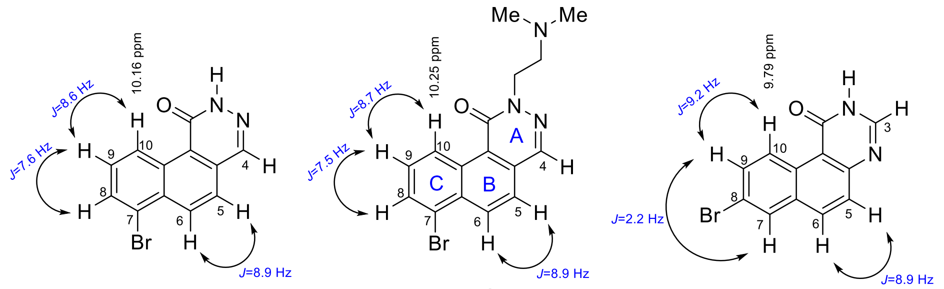

14 **15** **20**

Figure S1. The coupling constants between protons in naphthalene moiety of benzophthalazinones **14**, **15** and benzoquinazolinone **20** [32].

The ^1H NMR spectra of **14** and **15** revealed the absence of one proton from the naphthalene skeleton and the simultaneous presence of proton N=CH at 8.57 ppm (**14**, DMSO-d_6) and 8.28 ppm (**15**, $CDCl_3$). Moreover, the observed correlations in the ^1H, ^1H COSY spectrum of **15** indicated the presence of two groups of protons in naphthalene moiety (two protons of the B ring: 7.72, 8.68 ppm and three protons of the C ring: 8.02, 7.63 and 10.25 ppm. The analysis of the coupling constants between hydrogens of the C ring suggested that three hydrogen atoms could be located at the C8, C9, C10 positions or alternatively, at the C7, C8, C9, and they cannot be separated by the bromine atom as it is in the case of the compound **20**. Therefore, the consideration about the location of the bromine substituent in the

position C8 or C9 can be ignored [32]. On the other hand, the observed presence the strongly shifted to the lower field doublet signals in spectra of **14**, **15** and **20** [32], which must be caused by a neighbouring electron-withdrawing group, such as C=O. Thus, a signal at 10.25 ppm in **15** and 10.16 ppm in **14** corresponds to the proton connected with the carbon atom C10 and *ipso facto*, the bromine atom in **15**, as well as, in **14** should be attached to the C7.

S1.4. NMR data analysis of 2-[2-(dimethylamino)ethyl]phthalazin-1(2*H*)-one **19** and **19•HCl**

^{1}H and ^{13}C NMR data analysis was performed for 2-[2-(dimethylamino)ethyl]phthalazin-1(2*H*)-one **19** [38] and its HCl salt (**19•HCl**, generated in situ in chloroform solution). In the ^{1}H NMR spectrum of the HCl salt, a similar direction of changes of proton chemical shifts were observed as in the case of compounds **16**, **16•HCl** (Table S1 and S2), however, the resonance proton signals of methylene and methyl groups were broad singlets. The protonation of the amine nitrogen atom in **19** also led to changes in carbon chemical shifts. However, the direction of changes, especially for carbon atoms adjacent to NH^{+}, was opposite to that observed in proton resonance (Table S2 and S3)[37,38].
The ^{13}C resonance signals of ethylene and methyl groups were shifted upfield, from 57.2 (CH_2), 48.6 (CH_2), 45.4 ppm (Me) to 55.1, 45.6 and 43.3 ppm, respectively (Table S3). On the other hand, for example, the chemical shift of the C=O carbon atom was downfield from 159.4 to 159.9 ppm. Similar dependencies can be observed for other carbon atoms (Table S3). Thus, the observed changes in chemical shifts induced by the protonation of a nitrogen atom are consistent with examples from the literature [35-38].

Table S1. ^{1}H NMR chemical shifts of **16** and **16•HCl**

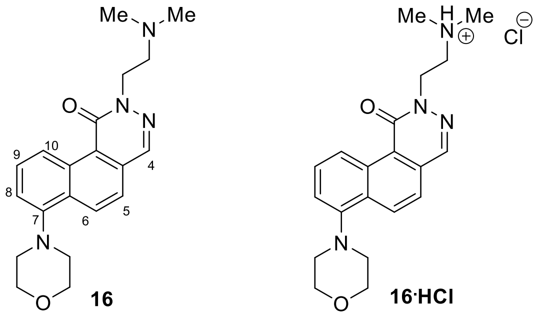

Entry		**16** δ1 (CDCl3, ppm)	**16•HCl** δ2 (CDCl3, ppm)	Δδ δ2-δ1 (ppm)
1	NH	-	12.97 (s)	-
2	C10-H	9.95 (d	9.80 (d)	-0.15
3	C9-H	7.77 – 7.71 (m)	7.76 (dd)	≈0.02
4	C8-H	7.41(d)	7.43 (d)	0.02
6	C6-H	8.72 (d)	8.77 (d)	0.05
7	C5-H	7.64 (d)	7.68 (d)	0.04
8	C4-H	8.28 (s)	8.34 (s)	0.06
9	CH_2	4.51 (t)	4.86 (t)	0.35
10	CH_2OCH_2	4.01 (d)	4.04 – 3.98 (m)	≈0
11	CH_2NCH_2	3.14 – 3.07 (m)	3.14 – 3.08 (m)	≈0
12	CH_2	2.85 (t)	3.61 (dd)	0.76
13	2xMe	2.37 (s)	2.93 (d)	0.56

Table S2. ^{1}H NMR chemical shifts of **19** and **19•HCl**

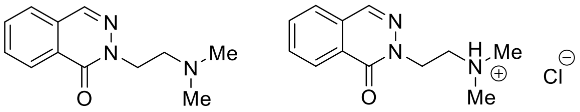

19 **19·HCl**

Entry		**19** [39] δ1 (CDCl3, ppm)	**19•HCl** δ2 (CDCl3, ppm)	Δδ δ2-δ1 (ppm)
1	NH	-	12.74 (s)	-
2	C8-H	8.46 –8.40 (m)	8.37 (d)	-0.06
3	C7-H	7.83–7.66 (m)	7.86 – 7.81 (m)	≈0
4	C6-H		7.80 – 7.76 (m)	
5	C5-H		7.72 (d)	
6	C4-H	8.17 (s)	8.23 (s)	0.06
7	CH_2	4.38 (t)	4.71 (br s)	0.33
8	CH_2	2.78 (t)	3.56 (br s)	0.78
9	2xMe	2.33 (s)	2.91 (br s)	0.58

Table S3. ^{13}C NMR chemical shifts of **19** and **19•HCl**

Entry		**19** [39] δ1 (CDCl3, ppm)	**19•HCl** δ2 (CDCl3, ppm)	Δδ δ2-δ1 (ppm)
1	C=O	159.4	159.9	0.5
2		137.8	139.5	1.7
3		132.9	133.9	1.0
4		131.5	132.3	0.8
5		129.6	129.8	0.2
6		127.8	127.7	-0.1
7		126.6	126.8	0.2
8		125.9	126.7	0.8
9	CH_2	57.2	55.1	-2.1
10	CH_2	48.6	45.6	-3.0
11	2xMe	45.4	43.3	-2.1

S2. Experimental

S2.1 General information

Melting points were determined on a Boetius hot stage apparatus and were uncorrected. ^{1}H, ^{13}C NMR spectra were recorded on a Bruker Advance III spectrometer at 600 MHz and 150 MHz respectively. Chemical shifts (δ_H, δ_C) were quoted in parts per million (ppm), referenced to the signal of TMS or to the appropriate residual solvent peak ($CDCl_3$ at 7.26 ppm or DMSO-d_6 at 2.50 ppm for ^{1}H NMR and $CDCl_3$ at 77.16 ppm or DMSO-d_6 at 39.52 ppm for ^{13}C NMR) [40]. Coupling constants (*J*) were quoted in Hertz (Hz). 2D Homonuclear ^{1}H, ^{1}H COSY and heteronuclear ^{1}H, ^{13}C HMQC, HMBC spectra were used to assign the proton and carbon signals. IR spectra were recorded on a Nexus FT-IR spectrometer.
LC/HRMS analyses were performed using an Agilent Technologies HPLC 1290 coupled to an Agilent Technologies 6550 Accurate Mass Q-TOF LC–MS mass spectrometer equipped with a JetStream Technology ion source housed in the Department of Pathophysiology, Medical University of Lublin, Poland. Internal mass calibration was enabled; reference ions of $m/z = 121.0509$ and 922.0098 were used.
The analytical thin layer chromatography tests (TLC) were carried out on Sigma-Aldrich (Supelco) silica gel plates (Kiselgel 60 F254, layer thickness 0.2 mm) and the spots were visualized using UV lamp. The flash column chromatography purifications were performed on Fluka silica gel (Silica gel 60, 0.040–0.063 mm).
Alkyllithium solutions (*s*-butylithium in cyclohexane (*s*-BuLi), *n*-butyllitium in hexanes (*n*-BuLi), methyllithium in diethyl ether (MeLi), isopropylithium in pentane (*i*-PrLi) – Sigma-Aldrich) were each time titrated before use [41].
All reactions with organolithium and organopalladium compounds were performed under an argon atmosphere using standard Schlenk technique. THF, toluene were distilled from sodium benzophenone ketyl prior to use.
Commercially available reagents: Hydrazine monohydrate, methylhydrazine, 2-chloro-*N*,*N*-dimethylethylamine hydrochloride, *t*-BuOK, tris(dibenzylideneacetone)-dipalladium(0) ($Pd_2(dba)_3$), (*R*)-2,2′-bis(diphenylphosphino)-1,1′-binaphthalene (*R*-BINAP) were purchased from Sigma-Aldrich and were used without further purification.
N-Phenylnaphthalene-1-carboxamide [42-44], 3-hydroxy-2-phenyl-2,3-dihydro-1*H*-isoindol-1-one **2** [45], 2-[2-(dimethylamino)ethyl]-2*H*-phthalazin-1-one **19** [39] were obtained as described previously and their characterization data were in agreement with reported analysis. The characterization data of 2-(2-methylpropanoyl) benzaldehyde **8** [26], 3-hydroxy-2,3-dihydro-1*H*-inden-1-one **5c** [46] were in agreement with already reported analysis.

S2.2 Synthesis of 3-hydroxy-2-phenyl-2,3-dihydro-1*H*-benzo[*e*]isoindol-1-one (**1**)

To the stirred at 0 °C under argon solution of *N*-phenylnaphthalene-1-carboxamide (4.04×10^{-3} mol) in THF (30 mL) *n*-BuLi in hexanes (8.89×10^{-3} mol) was added dropwise. The solution was held at 0 °C for 1 hour, then DMF (2.68×10^{-2} mol) was added dropwise. Reaction mixture after 1 hour at 0 °C was warmed to rt and stirred under these conditions for 2 hour. After that methanol (25 mL) was added and stirring was continued for 0.5 hour. Then solvent were removed under reduced pressure. Water (25 mL) and DCM (25 mL) were added to obtained residue and the whole lot was neutralised with HCl_{aq} (1:1, v/v). The water layer was separated and extracted subsequently with DCM (3×15 mL). The organic phase was dried over $MgSO_4$ and concentrated till dryness. The crude material was separated by flash chromatography (Hex-AcOEt 4:1, 3:1, 2:1).

White solid; Yield: 665 mg, 60%; mp: 169–171 °C; R_f=0.44 (Hex-AcOEt 2:1);
FT-IR (KBr): ν = 3309, 3056, 1676, 1657, 1596, 1503, 1403, 1386 cm^{-1};
^{1}H NMR (600 MHz, $CDCl_3$): δ = 9.05 (d, *J* = 8.4 Hz, 1H, ArH), 8.00 (d, *J* = 8.2 Hz, 1H, ArH), 7.86 (d, *J* = 8.1 Hz, 1H, ArH), 7.77 (dd, *J* = 8.6, 1.0 Hz, 2H, PhH), 7.65 (d, *J* = 8.3 Hz, 1H, ArH), 7.61 (ddd, *J* = 8.3, 6.9, 1.3 Hz, 1H, ArH), 7.56 (ddd, *J* = 8.1, 6.9, 1.2 Hz, 1H, ArH), 7.42 – 7.36 (m, 2H, PhH), 7.21 – 7.17 (m, 1H, PhH), 6.34 (d, *J* = 11.1 Hz, 1H, OH), 3.06 (d, *J* = 11.2 Hz, 1H, CH) ppm;
^{13}C NMR (151 MHz, $CDCl_3$): δ = 167.4 (C=O), 143.1, 137.2, 134.2, 1343.0, 129.1, 128.9, 128.5, 128.4, 127.2, 125.4, 125.2, 124.2, 121.9, 119.7, 82.3 (C3) ppm;
HRMS (ESI) m/z: calcd for $C_{18}H_{14}NO_2$ [M+H]$^+$ 276.1019, found 276.1015.

S2.3 Reactions of 3-hydroxy-1*H*-benzo[*e*]isoindol-1-one **1** and 3-hydroxy-1*H*-isoindol-1-one **2** with organolithium compounds: *s*-BuLi, *n*-BuLi, MeLi and *i*-PrLi.

S2.3.1 Synthesis of 3-hydroxy-2,3-dihydro-1*H*-inden-1-one **3** and **5** and 3-anilino-2,3-dihydro-1*H*-inden-1-one **4** and **6** derivatives.

General procedure:

Under argon, to the solution of 3-hydroxyisoindolinone **1** or **2** (1.33×10^{-3} mol) in THF (20 mL) at 0 °C, the solution of alkyllithium compound (*s*-BuLi, *n*-BuLi, MeLi or *i*-PrLi, 2.93×10^{-3} mol) was added dropwise. The solution was stirred at 0 °C for 1.5 h. Next reaction mixture was warmed to rt and stirred under these conditions for 1.5 h. After that methanol (15 mL) was added and stirring was continued for 0.5 h. Then solvents were removed under reduced pressure. Water (40 mL) and DCM (40 mL) were added to obtained residue and the whole lot was neutralised with HCl_{aq} (1:1, v/v). The water layer was separated and extracted subsequently with DCM (3×15 mL). The organic phase was dried over $MgSO_4$ and concentrated till dryness. The crude material was separated by flash chromatography.

2-Ethyl-3-hydroxy-2-methyl-2,3-dihydro-1*H*-cyclopenta[*a*]naphthalen-1-one (**3a**) (as a mixture of diastereoisomers **3a′**, **3a″**)

Diastereoisomer **3a′**:

Yellow oil; Yield: 11 mg, 9%; R_f= 0.32 (DCM-Hex-AcOEt 6:1:0.5);
FT-IR (KBr): ν = 3336, 3056, 2962, 2919, 1697, 1575, 1517, 1440 cm^{-1};
^{1}H NMR (600 MHz, $CDCl_3$): δ = 9.11 (d, *J* = 8.4 Hz, 1H, ArH), 8.14 (d, *J* = 8.4 Hz, 1H, ArH), 7.92 (d, *J* = 8.2 Hz, 1H, ArH), 7.76 (d, *J* = 8.4 Hz, 1H, ArH), 7.71-7.66 (m, 1H, ArH), 7.62 – 7.57 (m, 1H, ArH), 5.07 (d, *J* = 5.7 Hz, 1H, C3-H), 2.09 (d, *J* = 7.1 Hz, 1H, OH), 1.77 (dq, *J* = 15.0, 7.5 Hz, 1H, CH_2), 1.66 (dq, *J* = 14.9, 7.5 Hz, 1H, CH_2), 1.33 (s, 3H, Me), 0.89 (t, *J* = 7.5 Hz, 3H, Me) ppm;
^{13}C NMR (151 MHz, $CDCl_3$): δ = 208.0 (C=O), 155.0, 136.2, 133.8, 129.5, 129.1, 128.8, 128.2, 127.1, 124.9, 122.0, 78.5 (C3), 54.9 (C2) 27.3, 20.7, 9.5 ppm;
HRMS (ESI) m/z: calcd for $C_{16}H_{17}O_2$ [M+H]$^+$ 241.1223, found 241.1222.

Diastereoisomer **3a″**:

Yellow oil; Yield: 11 mg, 9%; R_f= 0.36 (DCM-Hex-AcOEt 6:1:0.5);
FT-IR (KBr): ν = 3370, 3057, 2928, 1694, 1573, 1516, 1462 cm^{-1};
^{1}H NMR (600 MHz, $CDCl_3$): δ = 9.13 (d, *J* = 8.3 Hz, 1H, ArH), 8.15 (d, *J* = 8.4 Hz, 1H, ArH), 7.92 (d, *J* = 8.2 Hz, 1H, ArH), 7.78 (d, *J* = 8.4 Hz, 1H, ArH), 7.71-7.67 (m, 1H, ArH), 7.62 – 7.57 (m, 1H, ArH), 5.18 (d, *J* =

7.3 Hz, 1H, C3-H), 1.91 (d, *J* = 8.1 Hz, 1H, OH), 1.84 – 1.72 (m, 2H, CH_2), 1.21 (s, 3H, Me), 0.95 (t, *J* = 7.5 Hz, 3H, Me) ppm;
^{13}C NMR (151 MHz, $CDCl_3$): δ = 208.6 (C=O), 155.3, 136.4, 133.8, 129.2, 128.9, 128.2, 127.2, 124.9, 122.4, 75.2 (C3), 54.8 (C2), 29.45, 18.5, 9.1 ppm;
HRMS (ESI) m/z: calcd for $C_{16}H_{17}O_2$ $[M+H]^+$ 241.1223, found 241.1221.

Mixture of diastereoisomers **3a′** and **3a″**, molar ratio **3a′** : **3a″** 0.4 : 0.6 (^{1}H NMR $CDCl_3$).
Yellow oil; Yield: 35 mg, 29%; R_f= 0.36, 0.32 (DCM-Hex-AcOEt 6:1:0.5);
^{1}H NMR (600 MHz, $CDCl_3$): δ = 9.13 – 9.08 (m, 1H, ArH **3a′**, **3a″**), 8.13 (d, *J* = 8.4 Hz, 1H, ArH **3a′**, **3a″**), 7.91 (d, *J* = 8.2 Hz, 1H, ArH **3a′**, **3a″**), 7.78 – 7.73 (m, 1H, ArH **3a′**, **3a″**), 7.70 – 7.65 (m, 1H, ArH **3a′**, **3a″**), 7.62 – 7.56 (m, 1H, ArH **3a′**, **3a″**), 5.17 (s, 0.6H, C3-H **3a″**), 5.05 (s, 0.4H, C3-H **3a′**), 2.30 (s, 0.4H, OH **3a′**), 2.14 (s, 0.6H, OH **3a″**), 1.82 – 1.72 (m, 1.4H CH_2 **3a′**, **3a″**), 1.69 – 1.59 (m, 0.6H, CH_2 **3a′**), 1.32 (s, 1.2H, Me **3a′**), 1.20 (s, 1.8H, Me **3a″**), 0.94 (t, *J* = 7.5 Hz, 1.8H, Me **3a″**), 0.88 (t, *J* = 7.5 Hz, 1.2H, Me **3a′**) ppm;
^{13}C NMR (151 MHz, CDCl3): δ = 208.8 (**3a″**), 208.1 (**3a′**), 155.3 (**3a″**), 155.1 (**3a′**), 136.4 (**3a″**), 136.2 (**3a′**), 133.8 (**3a″**), 133.8 (**3a′**), 129.4 (**3a′**), 129.2 (**3a″**), 129.1 (**3a′**), 128.9 (**3a″**), 128.8 (**3a′**), 128.2 (**3a′**), 128.2 (**3a″**), 127.2 (**3a″**), 127.1 (**3a′**), 124.9 (**3a″**), 124.8 (**3a′**), 122.4 (**3a″**), 122.0 (**3a′**), 78.4 (**3a′**), 75.1 (**3a″**), 54.9 (**3a″**), 54.8 (**3a′**), 29.5 (**3a″**), 27.3 (**3a′**), 20.6 (**3a′**), 18.5 (**3a″**), 9.4 (**3a′**), 9.1(**3a″**) ppm.

3-Hydroxy-2-propyl-2,3-dihydro-1*H*-cyclopenta[*a*]naphthalen-1-one (**3b**)
Yellowish wax; Yield: 34 mg, 40%; R_f = 0.05 (Hex-AcOEt 7:1)
FT-IR (KBr): ν = 3337, 3056, 2952, 2869, 1671, 1518 cm^{-1};
^{1}H NMR (600 MHz, $CDCl_3$): δ = 9.10 (d, *J* = 8.4 Hz, 1H, 9ArH), 8.12 (d, *J* = 8.4 Hz, 1H, ArH), 7.91 (d, *J* = 8.2 Hz, 1H, ArH), 7.73 (d, *J* = 8.4 Hz, 1H, ArH), 7.68 (ddd, *J* = 8.3, 7.0, 1.2 Hz, 1H, ArH), 7.59 (ddd, *J* = 8.1, 7.0, 1.2 Hz, 1H, ArH), 5.13 (dd, *J* = 6.3, 2.8 Hz, 1H, C3-H), 2.70 – 2.66 (m, 1H, C2-H), 2.27 (d, *J* = 7.2 Hz, 1H, OH), 2.09 – 2.01 (m, 1H, CH_2), 1.70 – 1.58 (m, 3H, CH_2), 1.03 (t, *J* = 7.2 Hz, 3H, Me) ppm;
^{13}C NMR (151 MHz, $CDCl_3$): δ = 205.0 (C=O), 155.6, 136.3, 133.7, 130.2, 129.2, 128.7, 128.2, 127.3, 124.85, 122.1, 74.8 (C3), 58.6 (C2), 31.5, 20.8, 14.2 ppm;
HRMS (ESI) m/z: calcd for $C_{16}H_{17}O_2$ $[M+H]^+$ 241.1223, found 241,1223.

3-Hydroxy-2,3-dihydro-1*H*-cyclopenta[*a*]naphthalen-1-one (**3c**)
Beige solid; Yield: 338 mg, 35%; mp: 123–125 °C; R_f = 0.31 (Hex-AcOEt 1:1);
FT-IR (KBr): ν = 3293, 3188, 3054, 2959, 2914, 1714, 1574, 1514, 1439 cm^{-1};
^{1}H NMR (600 MHz, $CDCl_3$): δ = 9.08 (d, *J* = 8.3 Hz, 1H, ArH), 8.10 (d, *J* = 8.4 Hz, 1H, ArH), 7.89 (d, *J* = 8.1 Hz, 1H, ArH), 7.72 (d, *J* = 8.4 Hz, 1H, ArH), 7.69 – 7.64 (m, 1H, ArH), 7.61 – 7.56 (m, 1H, ArH), 5.46 (dd, *J* = 6.4, 2.3 Hz, 1H, C3-H), 3.21 (dd, *J* = 18.4, 6.6 Hz, 1H, C2-H_2), 2.72 (dd, *J* = 18.4, 2.7 Hz, 1H, C2-H_2), 2.53 (br s, 1H, OH) ppm;
^{13}C NMR (151 MHz, $CDCl_3$): δ = 203.5 (C=O), 157.3, 136.5, 133.6, 130.7, 129.3, 128.6, 128.2, 127.4, 124.7, 122.4, 68.3 (C3), 47.8 (C2) ppm;
HRMS (ESI) m/z: calcd for $C_{13}H_{11}O_2$ $[M+H]^+$ 199.0754, found 199.0756.

3-Anilino-2,3-dihydro-1*H*-cyclopenta[*a*]naphthalen-1-one (**4c**)
Yellowish solid; Yield: 21 mg, 17%; mp: 204–206 °C; R_f = 0.36 (Hex-AcOEt 1:1);
FT-IR (KBr): ν = 3401, 3048, 2918, 2850, 1693, 1599, 1509, 1433 cm^{-1};
^{1}H NMR (600 MHz, $CDCl_3$): δ = 9.18 (d, *J* = 8.3 Hz, 1H, ArH), 8.12 (d, *J* = 8.4 Hz, 1H, ArH), 7.93 (d, *J* = 8.2 Hz, 1H, ArH), 7.74 (d, *J* = 8.4 Hz, 1H, ArH), 7.71 (ddd, *J* = 8.3, 7.1, 1.2 Hz, 1H, ArH), 7.63 – 7.59 (m, 1H, ArH), 7.27 – 7.22 (m, 2H, PhH), 6.80-6.82 (m, 1H, PhH), 6.74 (d, *J* = 7.7 Hz, 2H, PhH), 5.27 (dd, *J* = 6.5, 2.7 Hz, 1H, C3-H), 4.05 (s, 1H, NH), 3.32 (dd, *J* = 18.5, 6.6 Hz, 1H, CH_2), 2.70 (dd, *J* = 18.5, 2.9 Hz, 1H, CH_2) ppm;
^{13}C NMR (151 MHz, $CDCl_3$): δ = 204.2 (C=O), 157.0, 147.0, 136.5, 133.6, 131.2, 129.6, 129.3, 128.9, 128.2, 127.3, 124.6, 122.6, 118.5, 113.5, 51.6 (C3), 46.1 (C2) ppm;
HRMS (ESI) m/z: calcd for $C_{19}H_{16}NO$ $[M+H]^+$ 274.1226, found 274.1225.

3-Hydroxy-2,2-dimethyl-2,3-dihydro-1*H*-cyclopenta[*a*]naphthalen-1-one (**3d**)
Beige solid; Yield: 77 mg, 34%; mp: 116–117 °C (Et_2O);
FT-IR (KBr): ν = 3284, 3197, 3066, 2972, 2866, 1702, 1573, 1518, 1440 cm^{-1};
^{1}H NMR (600 MHz, $CDCl_3$): δ = 9.10 (d, *J* = 8.2 Hz, 1H, ArH), 8.15 (d, *J* = 8.4 Hz, 1H, ArH), 7.92 (d, *J* = 8.2 Hz, 1H, ArH), 7.77 (d, *J* = 8.4 Hz, 1H, ArH), 7.69 (ddd, *J* = 8.3, 7.0, 1.2 Hz, 1H, ArH), 7.60 (ddd, *J* = 8.1, 7.0, 1.2 Hz, 1H, ArH), 5.05 (s, 1H, C3-H), 1.99 (br s, 1H, OH), 1.36 (s, 3H, Me), 1.22 (s, 3H, Me) ppm;
^{13}C NMR (151 MHz, $CDCl_3$): δ = 208.8 (C=O), 154.8, 136.6, 133.9, 129.3, 129.1, 128.7, 128.4, 127.3, 125.0, 122.5, 78.1 (C3), 51.4 (C2), 23.3 (Me), 20.6 (Me) ppm;
HRMS (ESI) m/z: calcd for $C_{15}H_{15}O_2$ $[M+H]^+$ 227.1067, found 227.1064.

2-Ethyl-3-hydroxy-2-methyl-2,3-dihydro-1*H*-inden-1-one (**5a′**)
Yellowish oil; Yield: 19 mg, 11%; R_f = 0.32 (Hex-AcOEt 3:1);
FT-IR (KBr): ν = 3418, 3075, 2963, 2920, 1693, 1607, 1454 cm^{-1};
^{1}H NMR (600 MHz, $CDCl_3$): δ = 7.75 (d, *J* = 7.6 Hz, 1H, ArH), 7.71 – 7.66 (m, 2H, ArH), 7.50 – 7.45 (m, 1H, ArH), 4.97 (s, 1H, C3-H), 2.19 (br s, 1H, OH), 1.72 (dq, *J* = 15.0, 7.5 Hz, 1H, CH_2), 1.58 (dq, *J* = 14.9, 7.5 Hz, 1H, CH_2), 1.26 (s, 3H, Me), 0.86 (t, *J* = 7.5 Hz, 3H, Me) ppm;
^{13}C NMR (151 MHz, $CDCl_3$): δ = 207.7 (C=O), 153.2, 135.4, 135.2, 129.5, 125.4, 123.6, 78.7 (C3), 54.7 (C2), 26.8, 20.4, 9.3 ppm;
HRMS (ESI) m/z: calcd for $C_{12}H_{15}O_2$ $[M+H]^+$ 191.1067, found 191,1060.

Mixture of diastereoisomers **5a′** and **5a″** (molar ratio **5a′** : **5a**″ 0.4 : 0.6 (^{1}H NMR)).
Yellow oil; Yield: 66 mg, 39%; R_f = 0.28 (Hex-AcOEt 3:1);
^{1}H NMR (600 MHz, $CDCl_3$): δ = 7.75 (d, *J* = 7.6 Hz, 1H, ArH **5a′**, **5a″**), 7.73 – 7.65 (m, 2H, ArH **5a′**, **5a″**), 7.50 – 7.45 (m, 1H, ArH **5a′**, **5a″**), 5.09 (s, 0.6H, C3-H **5a″**), 4.97 (s, 0.4H, C3-H **5a′**), 2.16 (s, 0.4H, OH **5a′**), 2.02 (s, 0.6H, OH **5a″**), 1.76 – 1.66 (m, 1.4H, CH_2 **5a′**, **5a″**), 1.62 – 1.51 (m, 0.6H, CH_2 **5a′**), 1.26 (s, 1.2H, Me **5a′**), 1.14 (s, 1.8H, Me **5a″**), 0.91 (t, *J* = 7.5 Hz, 1.8H, Me **5a″**), 0.86 (t, *J* = 7.5 Hz, 1.2H, Me **5a′**) ppm;
HRMS (ESI) m/z: calcd for $C_{12}H_{15}O_2$ $[M+H]^+$ 191.1067, found 191,1066.

3-Anilino-2-ethyl-2-methyl-2,3-dihydro-1*H*-inden-1-one (**6a**)
Mixture of diastereoisomers **6a′** and **6a″** (molar ratio **6a′** : **6a″** 0.1 : 0.9 (^{1}H NMR)).
Brown thick oil; Yield: 95 mg, 40%; R_f = 0.70 (Hex-AcOEt 3:1);
FT-IR (KBr): ν = 3383, 3032, 2969, 2878, 1702, 1601, 1513, 1372 cm^{-1};
^{1}H NMR (600 MHz, $CDCl_3$): δ = 7.78 – 7.76 (m, 1H, ArH, **6a′**, **6a″**), 7.65 – 7.60 (m, 2H, ArH, **6a′**, **6a″**), 7.49 – 7.43 (m, 1H, ArH, **6a′**, **6a″**), 7.27 – 7.20 (m, 2H, ArH, **6a′**, **6a″**), 6.82 – 6.75 (m, 3H, ArH, **6a′**, **6a″**), 5.11 (d, *J* = 10.0 Hz, 0.1H, C3-H **6a′**), 4.99 (d, *J* = 10.1 Hz, 0.9H, C3-H **6a″**), 4.01 (d, *J* = 10.0 Hz, 0.9H, NH **6a″**), 3.88 (d, *J* = 9.9 Hz, 0.1H, NH **6a′**), 1.92 (dq, *J* = 14.9, 7.5 Hz, 0.1H, CH_2 **6a′**), 1.75 (dq, *J* = 14.8, 7.5 Hz, 0.1H, CH_2 **6a′**), 1.67 (dq, *J* = 15.0, 7.5 Hz, 0.9H, CH_2 **6a″**), 1.47 (dq, *J* = 14.8, 7.4, 0.9H, CH_2 **6a″**), 1.40 (s, 2.7H, Me **6a″**), 1.12 (s, 0.3H, Me **6a′**), 0.91 (t, *J* = 7.5 Hz, 0.3H, Me, **6a′**), 0.80 (t, *J* = 7.5 Hz, 2.7H, Me **6a″**) ppm;
^{13}C NMR (151 MHz, $CDCl_3$): δ = 207.7 (C=O), 153.1, 147.9, 135.6, 135.3), 135.1), 129.6), 129.0, 126.1, 125.2, 123.6, 123.6, 118.1, 113.0, 112.9, 62.6, 58.5, 54.9, 30.3, 27.8, 21.4, 19.7, 9.2 ppm;

^{1}H NMR (600 MHz, DMSO-d_6): δ= 7.76 – 7.71 (m, 1H, ArH, **6a′**, **6a″**), 7.68 (d, *J* = 7.5 Hz, 1H, ArH, **6a′**, **6a″**), 7.65 – 7.60 (m, 1H, ArH, **6a′**, **6a″**), 7.54 – 7.49 (m, 1H, ArH, **6a′**, **6a″**), 7.14 – 7.08 (m, 2H, ArH, **6a′**, **6a″**), 6.86 (d, *J* = 8.0 Hz, 1.8H, ArH, **6a″**), 6.83 (d, *J* = 8.0 Hz, 0.2H, **6a′**), 6.60 – 6.54 (m, 1H, **6a′**, **6a″**), 6.06 (d, *J* = 10.1 Hz, 0.9H, NH, **6a″**), 5.97 (d, *J* = 10.4 Hz, 0.1H, NH, **6a′**), 5.15 (d, *J* = 10.5 Hz, 0.9H, C3-H, **6a′**), 5.12 (d, *J* = 10.1 Hz, 0.1H, C3-H, **6a″**), 1.78 – 1.71 (m, 0.1H, CH_2 **6a′**), 1.65 – 1.58 (m, 0.1H, CH_2, **6a′**), 1.51 (dq, *J* = 15.1, 7.6 Hz, 0.9H, CH_2, **6a″**), 1.37 (dq, *J* = 14.8, 7.5 Hz, 0.9H, CH_2, **6a″**), 1.28 (s, 2.7H, Me, **6a″**), 0.99 (s, 0.1H, **6a′**), 0.82 (t, *J* = 7.5 Hz, 0.1H, Me, **6a′**), 0.73 (t, *J* = 7.5 Hz, 2.7H, Me, **6a″**) ppm;
^{13}C NMR (151 MHz, DMSO-d_6): δ= 207.4 (C=O), 153.2, 148.9, 135.0, 134.7, 129.00, 128.7, 125.8, 122.7, 116.1, 112.4, 61.1 (C3), 54.4 (C2), 27.1, 20.9, 8.6 ppm;
HRMS (ESI) m/z: calcd for $C_{18}H_{20}NO$ $[M+H]^+$ 266.1539, found 266,1534.

3-Hydroxy-2,3-dihydro-1*H*-inden-1-one (**5c**) *^{1}H NMR data are in agreement with previously reported [46]*
Beige solid; Yield: 60 mg, 30%; R_f = 0.34 (Hex-AcOEt 1:1);
^{1}H NMR (600 MHz, $CDCl_3$) d 7.74 (d, *J* = 7.7 Hz, 1H), ArH, 7.71 (d, *J* = 7.5 Hz, 1H, ArH), 7.70 – 7.66 (m, 1H, ArH), 7.51 – 7.47 (m, 1H, ArH), 5.43 (dd, *J* = 6.7, 2.8 Hz, 1H, C3-H), 3.11 (dd, *J* = 18.8, 6.8 Hz, 1H, CH_2), 2.62 (dd, *J* = 18.8, 2.9 Hz, 1H, CH_2), 2.52 (brs, 1H, OH).

3-Hydroxy-2,2-dimethyl-2,3-dihydro-1*H*-inden-1-one (**5d**)
Beige solid; Yield: 46 mg, 20%; mp: 91–93 ^oC; R_f = 0.42 (Hex-AcOEt 5:1);
FT-IR (KBr): ν = 3285, 3198, 3065, 2971, 2863, 1712, 1603, 1470 cm^{-1};
^{1}H NMR (600 MHz, $CDCl_3$): δ = 7.77 (d, *J* = 7.6 Hz, 1H, ArH), 7.74 – 7.68 (m, 2H, ArH), 7.53 – 7.47 (m, 1H, ArH), 4.96 (d, *J* = 7.8 Hz, 1H, C3-H), 2.08 (d, *J* = 8.0 Hz, 1H, OH), 1.29 (s, 3H, Me), 1.15 (s, 3H, Me) ppm;
^{13}C NMR (151 MHz, $CDCl_3$): δ = 208.2 (C=O), 152.6, 135.3, 134.4, 129.5, 125.7, 123.9, 78.1 (C3), 51.0 (C2), 22.9 (Me), 19.9 (Me) ppm;
HRMS (ESI) m/z: calcd for $C_{11}H_{13}O_2$ $[M+H]^+$ 177.0910, found 177.0908.

3-Anilino-2,2-dimethyl-2,3-dihydro-1*H*-inden-1-one (**6d**)
Beige solid; Yield: 115 mg, 35%; mp: 115–117 ^oC; R_f = 0.72 (Hex-AcOEt-DCM 3:1:0.5);
FT-IR (KBr): ν = 3365, 3079, 2968, 2865, 1704, 1596, 1522, 1496 cm^{-1};
^{1}H NMR (600 MHz, $CDCl_3$): δ = 7.79 (d, *J* = 7.6 Hz, 1H, ArH), 7.68 – 7.63 (m, 2H, ArH), 7.51 – 7.45 (m, 1H, ArH), 7.24 (dd, *J* = 8.4, 7.4 Hz, 2H, ArH), 6.81 – 6.75 (m, 3H, ArH), 4.98 (d, *J* = 9.8 Hz, 1H, C3-H), 3.96 (d, *J* = 9.7 Hz, 1H, NH), 1.42 (s, 3H, Me), 1.12 (s, 3H, Me) ppm;
^{13}C NMR (151 MHz, $CDCl_3$): δ = 209.0 (C=O), 152.7, 147.9, 135.3, 134.6, 129.6, 129.2, 125.9, 123.9, 118.0, 113.0, 62.0 (C3), 51.4 (C2), 24.3 (Me), 20.7 (Me) ppm;
HRMS (ESI) m/z: calcd for $C_{17}H_{18}NO$ $[M+H]^+$ 252.1383, found 252.1380.

S2.3.2 Synthesis of 2-phenyl-3-(propan-2-yl)-2,3-dihydro-1*H*-isoindol-1-one (**7**) and 2-(2-methylpropanoyl)benzaldehyde (**8**)
Analogous procedure as described in [25].
Under argon, to the solution of 3-hydroxyisoindolinone **2** (1.57×10^{-3} mol) in THF (25 mL) at -78 ^oC, the solution of *i*-PrLi in (3.46×10^{-3} mol) was added dropwise. The solution was stirred at -78 ^oC for 1.5 h. Next reaction mixture was warmed to rt and stirred under these conditions for 0.5 h. After that water (15 mL) was added and the mixture was adjusted to pH ≈ 2 with HCl_{aq} (1:1, v/v), and then the organic layer was separated. The water layer was extracted with a mixture of $CHCl_3$-THF (1:1, v/v, 3×15 mL). The organic phase was dried over $MgSO_4$ and concentrated till dryness. The crude material was separated by flash chromatography.

2-Phenyl-3-(propan-2-yl)-2,3-dihydro-1*H*-isoindol-1-one (**7**)
White solid; Yield: 134 mg, 12%; mp: 154–156 ^oC; R_f = 0.32 (Hex-AcOEt 6:1);
FT-IR (KBr): ν = 3059, 2963, 2873, 1673, 1597, 1500, 1393 cm^{-1};
^{1}H NMR (600 MHz, $CDCl_3$): δ = 7.97 – 7.93 (m, 1H, ArH), 7.59 – 7.54 (m, 4H, ArH), 7.53 – 7.50 (m, 1H, ArH), 7.48 – 7.43 (m, 2H, ArH), 7.27 – 7.23 (m, 1H, ArH), 5.15 (d, *J* = 3.2 Hz, 1H, C3-H), 2.29 (heptd, *J* = 7.0, 3.4 Hz, 1H, CH), 1.21 (d, *J* = 7.1 Hz, 3H, Me), 0.42 (d, *J* = 6.8 Hz, 3H, Me) ppm;
^{13}C NMR (151 MHz, $CDCl_3$): δ = 167.4, 143.0, 137.4, 133.4, 131.6, 129.3, 128.5, 125.8, 124.4, 124.3, 123.3, 66.3 (C3), 29.5, 19.2, 15.1 ppm;
HRMS (ESI) m/z: calcd for $C_{17}H_{18}NO$ $[M+H]^+$ 252.1383, found 252.1378.

2-(2-Methylpropanoyl)benzaldehyde (**8**) *^{1}H NMR data are in agreement with previously reported [26]*
Oil; Yield: 21 mg, 27%; R_f = 0.42 (Hex-AcOEt 6:1;
^{1}H NMR (600 MHz, $CDCl_3$): δ = 10.05 (s, 1H, CHO), 7.90 (dd, *J* = 7.5, 1.3 Hz, 1H, ArH), 7.67 – 7.59 (m, 2H, ArH), 7.56 (dd, *J* = 7.4, 1.2 Hz, 1H, ArH), 3.26 (hept, *J* = 6.9 Hz, 1H, CH), 1.22 (d, *J* = 6.9 Hz, 6H, 2×Me) ppm;
HRMS (ESI) m/z: calcd for $C_{11}H_{13}O_2$ $[M+H]^+$ 177.0910, found 177.0908.

S2.4 Synthesis of benzo[*f*]phthalazin-1(2*H*)-one derivatives **12**-**16**

S2.4.1 Synthesis of benzo[*f*]phthalazin-1(2*H*)-ones **12** and 4-amino-benzo[*f*]phthalazin-1(2*H*)-ones **13**

Typical procedure [28].

A mixture of 3-hydroxyisoindolinone **1** (2.56×10^{-3} mol) and hydrazine monohydrate (3 mL), in propan-1-ol (15 mL) was heated with stirring under reflux for 38 h. After the completion of the reaction (TLC check) the mixture was cooled and all volatile materials were removed under reduced pressure. To the residue water was added and neutralized with acetic acid. The separated solid was collected by filtration, washed with water and then dried by vacuum suction and next subjected to flash chromatography to give products **12** and **13**.

Benzo[*f*]phthalazin-1(2*H*)-one (**12a**)
Yellow solid; Yield: 147 mg, 29%; mp: 175–176 °C; R_f=0.4 (Hex-AcOEt 1:1);
FT-IR (KBr): ν = 3129, 3033, 1633, 1549, 1507, 1425 cm^{-1};
^{1}H NMR (600 MHz, DMSO-d_6): δ =13.02 (s, 1H, NH), 10.07 – 9.97 (m, 1H, ArH), 8.51 (s, 1H, N=CH), 8.41 (d, *J* = 8.5 Hz, 1H, ArH), 8.18 – 8.12 (m, 1H, ArH), 7.93 (d, *J* = 8.6 Hz, 1H, ArH), 7.86 – 7.77 (m, 2H, ArH) ppm;
^{13}C NMR (151 MHz, DMSO-d_6): δ = 160.9 (C=O), 138.2, 134.9, 133.8, 130.7, 129.4, 128.7, 128.7, 128.3, 127.3, 123.3, 122.3 ppm;
HRMS (ESI) m/z: calcd for $C_{12}H_9N_2O$ [M+H]$^+$ 197.0709, found 197.0709.

4-Aminobenzo[*f*]phthalazin-1(2*H*)-one (**13a**)
Beige solid; Yield: 134 mg, 25%; mp: 321–322 °C; R_f=0.08 (Hex-AcOE 1:1);
FT-IR (KBr): ν = 3409, 3337, 3138, 1655, 1603, 1545, 1509, 1481 cm^{-1};
^{1}H NMR (600 MHz, DMSO-d_6) δ = 11.91 (s, 1H, NH), 10.24 (d, *J* = 9.9 Hz, 1H, ArH), 8.39 (d, *J* = 8.8 Hz, 1H, ArH), 8.13 (dd, *J* = 7.4, 2.1 Hz, 1H, ArH), 8.07 (d, *J* = 8.9 Hz, 1H, ArH), 7.81 – 7.75 (m, 2H, ArH), 6.01 (s, 2H, NH_2) ppm;
^{13}C NMR (151 MHz, DMSO-d_6): δ = 159.5 (C=O), 146.5, 134.1, 133.7, 130.2, 128.3, 128.3, 128.1, 124.8, 123.0 120.6 ppm;
HRMS (ESI) m/z: calcd for $C_{12}H_{10}N_3O$ [M+H]$^+$ 212.0818, found 212.0815.

2-Methylbenzo[*f*]phthalazin-1(2*H*)-one (**12b**)
Orange solid; Yield: 54 mg, 24%; mp: 108–111 °C; R_f=0.74 (DCM-AcOEt 4:1);
FT-IR (KBr): ν = 3032, 1639, 1618, 1601, 1587, 1547, 1508, 1469, cm^{-1};
^{1}H NMR (600 MHz, $CDCl_3$): δ = 10.17 (d, *J* = 8.6 Hz, 1H, ArH), 8.24 (s, 1H, N=CH), 8.17 (d, *J* = 8.5 Hz, 1H, ArH), 7.97 (d, *J* = 8.0 Hz, 1H, ArH), 7.85 – 7.78 (m, 1H, ArH), 7.77 – 7.70 (m, 1H, ArH), 7.63 (d, *J* = 8.5 Hz, 1H, ArH), 3.99 (s, 3H, Me) ppm;
^{13}C NMR (151 MHz, $CDCl_3$): δ = 160.7 (C=O), 137.2, 134.8, 134.3, 130.5, 130.3, 129.1, 128.5, 128.4, 128.4, 122.9, 122.4, 40.5 (Me) ppm;
HRMS (ESI) m/z: calcd for $C_{13}H_{11}N_2O$ [M+H]$^+$ 211.0866, found 211.0861.

4-Amino-2-methylbenzo[*f*]phthalazin-1(2*H*)-one (**13b**)
Yellow-orange solid; Yield: 21 mg, 9%; mp: 228–231 °C; R_f=0.27 (Hex-AcOEt 1:1);
FT-IR (KBr): ν = 3475, 3415, 1637, 1618, 1443, cm^{-1};
^{1}H NMR (600 MHz, DMSO-d_6): δ = 10.35 – 10.11 (m, 1H, ArH), 8.38 (d, *J* = 8.9 Hz, 1H, ArH), 8.14 – 8.11 (m, 1H, ArH), 8.07 (d, *J* = 8.9 Hz, 1H, ArH), 7.82 – 7.75 (m, 2H, ArH), 6.12 (s, 2H, NH_2), 3.67 (s, 3H, Me) ppm;
^{13}C NMR (600 MHz, DMSO-d_6): δ = 157.9, 145.8, 133.9, 130.1, 128.4, 128.06, 124.4, 122.7, 120.3, 39.0 (Me) ppm;
HRMS (ESI) m/z: calcd for $C_{13}H_{12}N_3O$ [M+H]$^+$ 226.0975, found 226.0972.

S2.4.2 Synthesis of 7-bromo-2-[2-(dimethylamino)ethyl]benzo[*f*]phthalazin-1(2*H*)-one (**15**)

Step 1 - Bromination of benzo[*f*]phthalazinone **12a** - analogous procedure as described in [28].

To a suspension of benzophthalazinone **12a** (2.45×10^{-4} mol) in the acetate buffer solution pH≈5.8 (5 mL), was added potassium bromide (2.69×10^{-4} mol) and bromine (2.69×10^{-4} mol) at an ambient temperature. The mixture was then stirred for 6 h at an ambient temperature, and next the whole was heated to

boiling and stirred for 30 h until the bromine colour entirely disappeared. After cooling to an ambient temperature the separated solid was collected by filtration and washed with water (2 mL). The crude product **14** (Scheme 4, yield: 58 mg, 87%) was used in the next step without additional purification.

7-Bromobenzo[*f*]phthalazin-1(2*H*)-one (**14**)
^{1}H NMR *diagnostic spectrum* (600 MHz, DMSO-d_6): δ = 13.15 (s, 1H, NH), 10.16 (d, *J* = 8.6 Hz, 1H, ArH), 8.66 (d, *J* = 8.9 Hz, 1H, ArH), 8.57 (s, 1H, N=CH), 8.18 (d, *J* = 7.6 Hz, 1H, ArH), 8.09 (d, *J* = 8.9 Hz, 1H, ArH), 7.77 – 7.73 (m, 1H, ArH) ppm.

Step 2 – Alkylation of 7-bromobenzo[*f*]phthalazinone **14** – analogous procedure as described in [39].
To the solution of sodium methoxide (6.32×10^{-4} mol) in dry methanol (15 mL) was added bromophthalazinone **14** (2.11×10^{-4} mol) and (2-chloroethyl)dimethyl ammonium chloride (4.22×10^{-4} mol). The mixture was heated at reflux for 30 hours. Afterwards, the inorganic material was collected by filtration, washed with dry methanol and, the filtrate was evaporated to dryness. The residue was subjected to flash chromatography (MeOH-$CHCl_3$ 1:1) to give the pure product **15**.

7-Bromo-2-[2-(dimethylamino)ethyl]benzo[*f*]phthalazin-1(2*H*)-one (**15**)
Yellow-orange solid; Yield: 44 mg, 60%; mp: 130–133 °C; R_f=0.44 (MeOH-$CHCl_3$ 1:1);
FT-IR (KBr): ν = 3056, 2952, 2869, 1671, 1575, 1518, 1466 cm^{-1};
^{1}H NMR (600 MHz, $CDCl_3$): δ = 10.25 (d, *J* = 8.7 Hz, 1H, 10ArH), 8.68 (d, *J* = 8.9 Hz, 1H, ArH), 8.28 (s, 1H, N=CH), 8.02 (dd, *J* = 7.5, 0.8 Hz, 1H, ArH), 7.72 (d, *J* = 8.9 Hz, 1H, ArH), 7.63 (dd, *J* = 8.6, 7.7 Hz, 1H, ArH), 4.51 (t, *J* = 6.9 Hz, 2H, CH_2), 2.86 (t, *J* = 6.9 Hz, 2H, CH_2), 2.36 (s, 6H, 2×Me) ppm;
^{13}C NMR (151 MHz, $CDCl_3$): δ = 160.1 (C=O), 137.1, 133.6, 132.6, 132.5, 132.1, 130.5, 129.2, 128.3, 123.9, 123.1, 122.9, 57.4, 50.0, 45.7 (2×Me) ppm;
HRMS (ESI) m/z: calcd for $C_{16}H_{17}BrN_3O$ [M+H]$^+$ 346.0549, found 346.0545.

S2.4.3 Synthesis of 2-[2-(dimethylamino)ethyl]-7-(morpholin-4-yl)benzo[*f*]phthalazin-1(2*H*)-one (**16**)
Analogous procedure as described in [31].
The reaction was carried out under an argon atmosphere in an oven dried resealable Schlenk flask. A resealable Schlenk flask was charged with $Pd_2(dba)_3$ (1 mol%), (*R*)-BINAP (15 mol%) and freshly distilled toluene (5 mL). The contents of the flask was stirred for 1 minute, then morpholine (2.4 equiv.) was added and whole was again stirred and heated at ≈100 °C for 20 minutes. After this time reaction was cooled and next bromophthalazinone **15** (5.78×10^{-5} mol), *t*-BuOK (1.2 equiv.) and toluene (2 mL) was added. This mixture was then stirred and heated at ≈100 °C for 20 h. After this time it was cooled and diluted with chloroform (2 mL). The solid was filtered off, washed with chloroform (2 mL) and the filtrate concentrated. The product was purified by flash chromatography

Oil; Yield: 9 mg, 44%; R_f=0.36 (MeOH-AcOEt-Hex 2:1:0.1);
FT-IR (KBr): ν = 3080, 2974, 2932, 2883, 2841, 1636, 1578, 1538, 1470 cm^{-1};
^{1}H NMR (600 MHz, $CDCl_3$): δ = 9.95 (d, *J* = 8.7 Hz, 1H, ArH), 8.72 (d, *J* = 8.8 Hz, 1H, ArH), 8.28 (s, 1H, N=CH), 7.77 – 7.71 (m, 1H, ArH), 7.64 (d, *J* = 8.8 Hz, 1H, ArH), 7.41 (d, *J* = 7.5 Hz, 1H, ArH), 4.51 (t, *J* = 7.0 Hz, 2H, CH_2), 4.01 (d, *J* = 4.0 Hz, 4H, CH_2OCH_2), 3.14 – 3.07 (m, 4H, CH_2NCH_2), 2.85 (t, *J* = 6.9 Hz, 2H, CH_2), 2.37 (s, 6H, 2×Me) ppm.

S2.4.4 Hydrochlorides **16•HCl** and **19•HCl**
N,*N*-Dimethyl-2-(7-(morpholin-4-yl)-1-oxobenzo[*f*]phthalazin-2(1*H*)-yl)ethan-1-aminium chloride (**16•HCl**)
FT-IR (KBr): ν = 3115, 2920, 2850, 1652, 1637, 1616, 1593, 1558, 1506, 1458 cm^{-1};
^{1}H NMR (600 MHz, $CDCl_3$): δ = 12.97 (s, 1H, NH$^+$), 9.80 (d, *J* = 8.7 Hz, 1H, ArH), 8.77 (d, *J* = 8.8 Hz, 1H, ArH), 8.34 (s, 1H, N=CH), 7.76 (dd, *J* = 8.6, 7.7 Hz, 1H, ArH), 7.68 (d, *J* = 8.8 Hz, 1H, ArH), 7.43 (d, *J* = 7.1 Hz, 1H ArH), 4.86 (t, *J* = 6.5 Hz, 2H, CH_2), 4.04 – 3.98 (m, 4H, CH_2OCH_2), 3.61 (dd, *J* = 11.3, 6.4 Hz, 2H, CH_2NH^+), 3.14 – 3.08 (m, 4H, CH_2NCH_2), 2.93 (d, *J* = 4.9 Hz, 6H, 2×Me) ppm;
^{13}C NMR (151 MHz, CDCl3): δ = 160.7 (C=O), 150.0, 138.6, 131.9, 130.8, 130.6, 130.2, 129.5, 124.0, 123.5, 122.1, 118.5, 67.5, 56.0, 54.0, 47.7, 44.1 ppm;
HRMS (ESI) m/z: calcd for $C_{20}H_{25}N_4O_2$ [M+H]$^+$ 353.1972, found 353,1977.

N,*N*-Dimethyl-2-(1-oxophthalazin-2(1*H*)-yl)ethan-1-aminium chloride (**19•HCl**)
^{1}H NMR (600 MHz, $CDCl_3$): δ= 12.74 (s, 1H, NH), 8.37 (d, *J* = 7.8 Hz, 1H, ArH), 8.23 (s, 1H, N=CH), 7.86 – 7.81 (m, 1H, ArH), 7.80 – 7.76 (m, 1H, ArH), 7.72 (d, *J* = 7.6 Hz, 1H, ArH), 4.71 (br s, 2H, CH_2), 3.56 (br s, 2H, CH_2), 2.91 (br s, 6H, 2×Me) ppm;
^{13}C NMR (151 MHz, $CDCl_3$): δ= 159.9 (C=O), 139.5 (N=CH), 133.9, 132.3, 129.8, 127.7, 126.8, 126.7, 55.1 (CH_2), 45.6 (CH_2), 43.3 (2×Me) ppm.

S2.5 ^{1}H, ^{13}C, 2D NMR spectra of compounds **1, 3–8, 12–16•HCl** and **19•HCl**

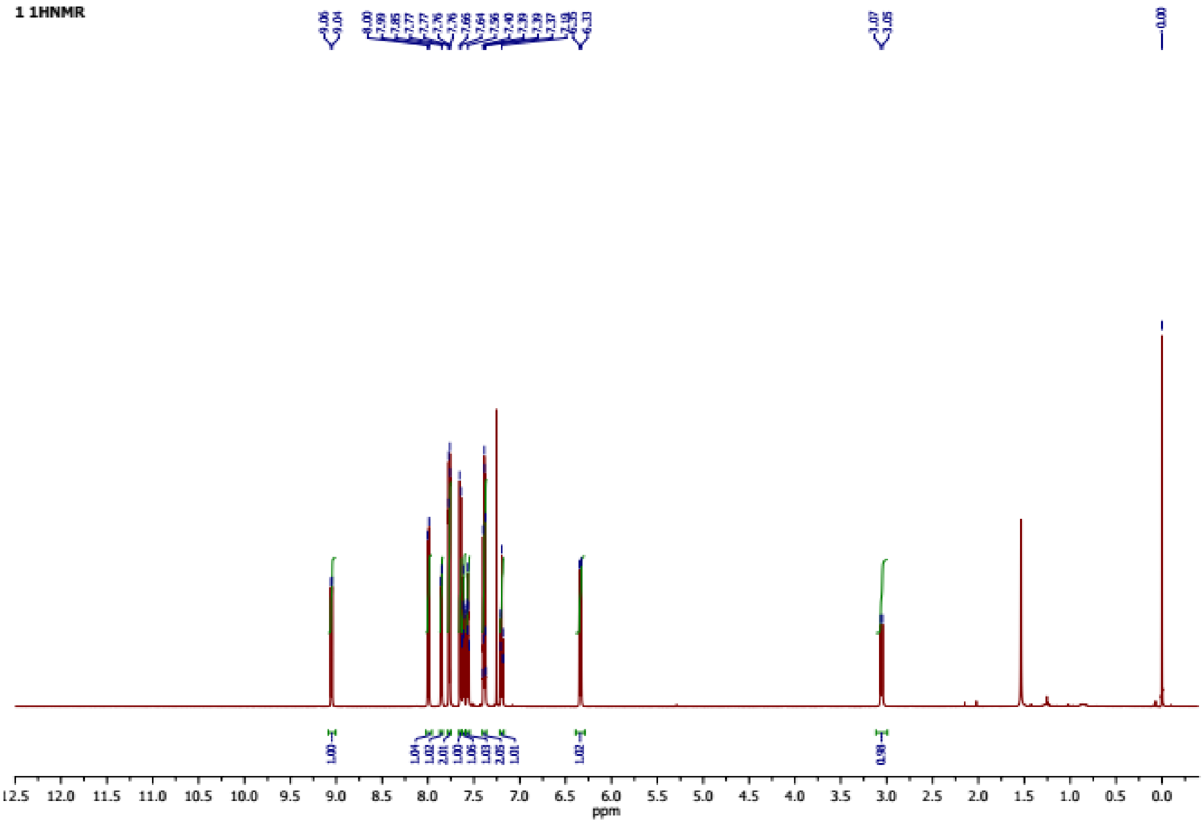

Figure S2. ^{1}H NMR spectrum of **1**.

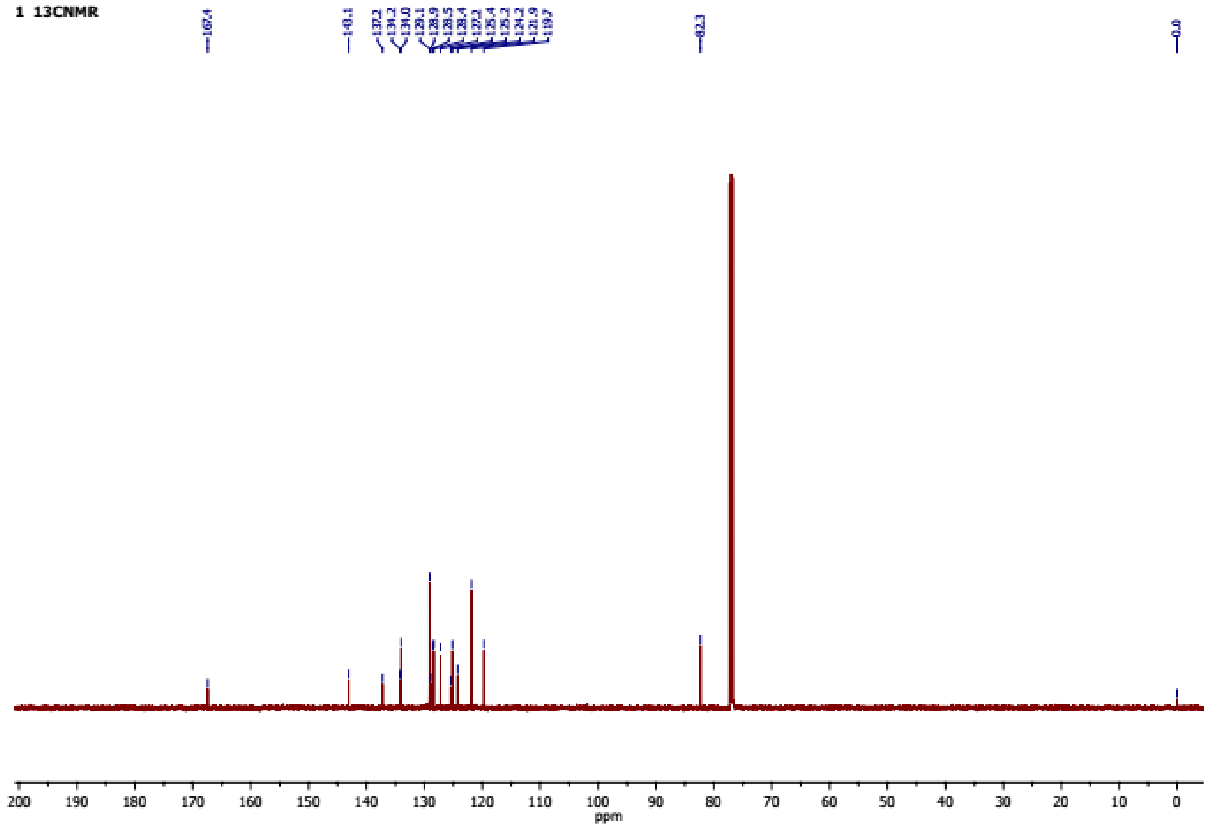

Figure S3. ^{13}C NMR spectrum of **1**.

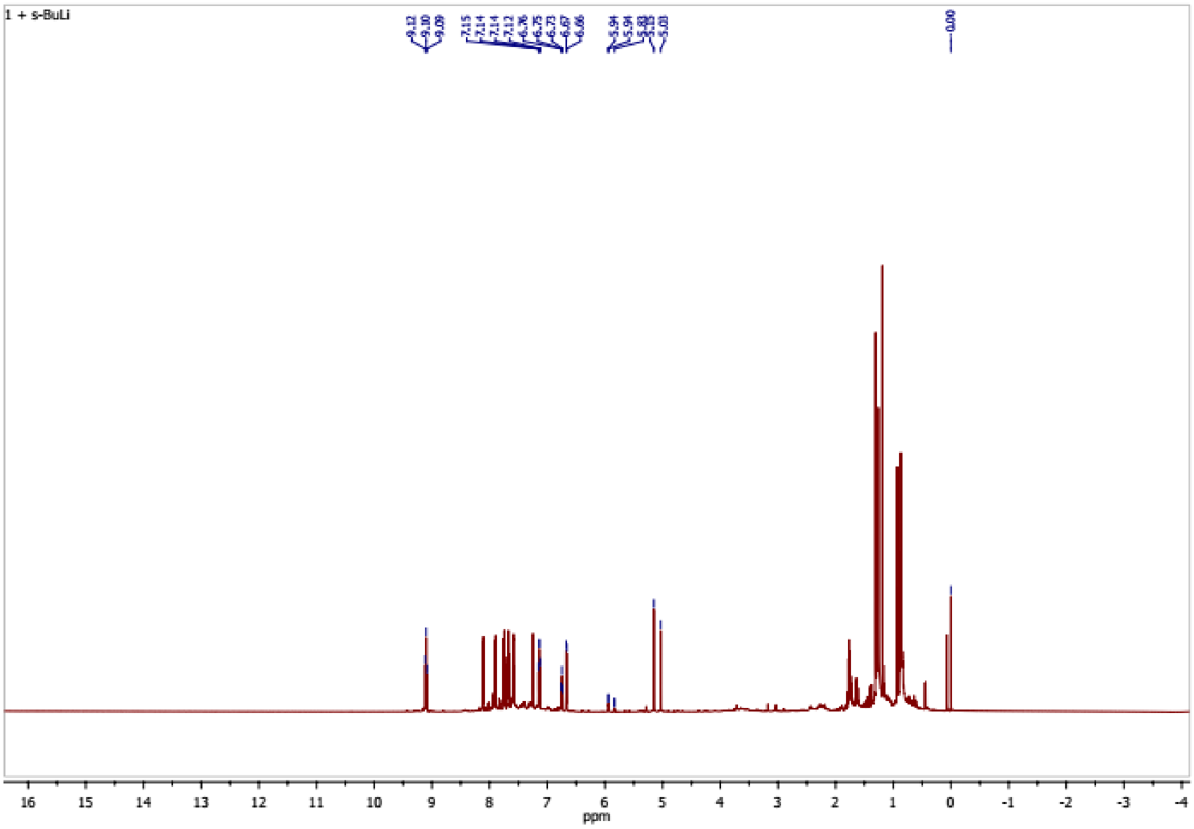

Figure S4. ¹H NMR spectrum of **1+*s*BuLi**.

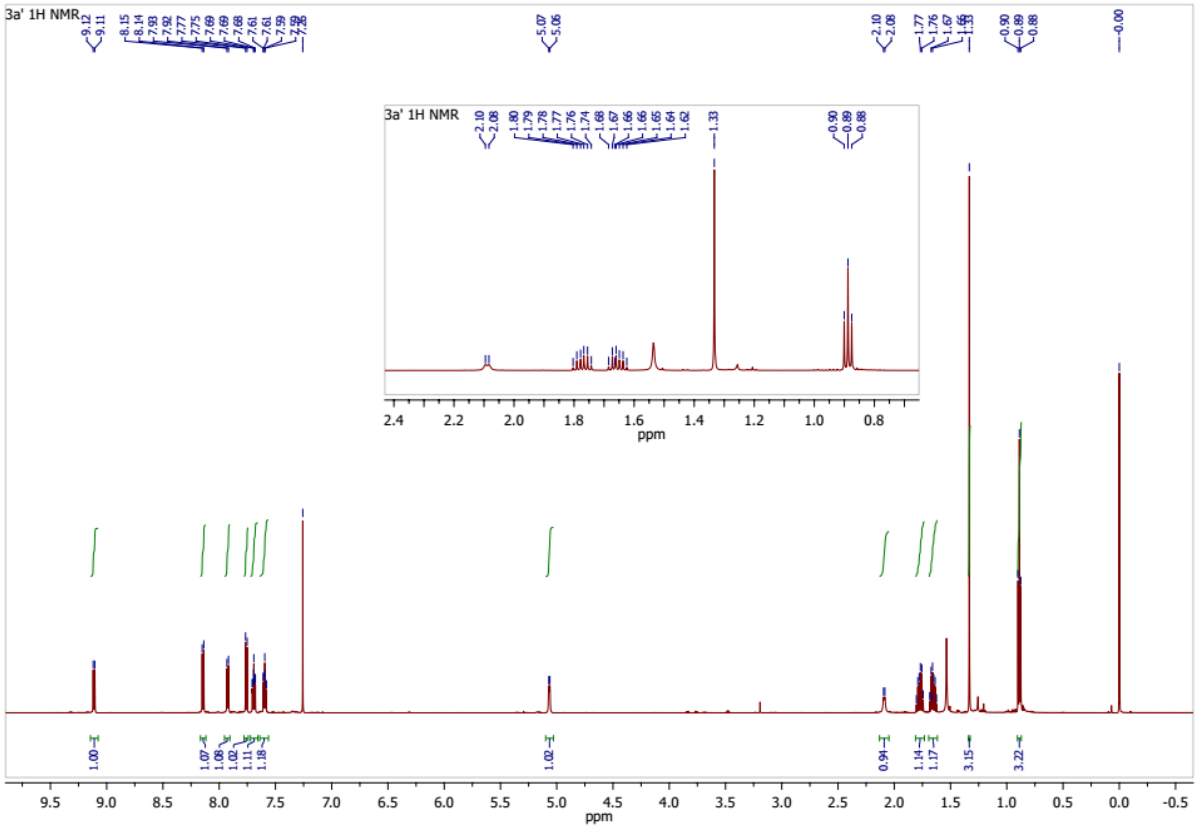

Figure S5. ¹H NMR spectrum of **3a′**.

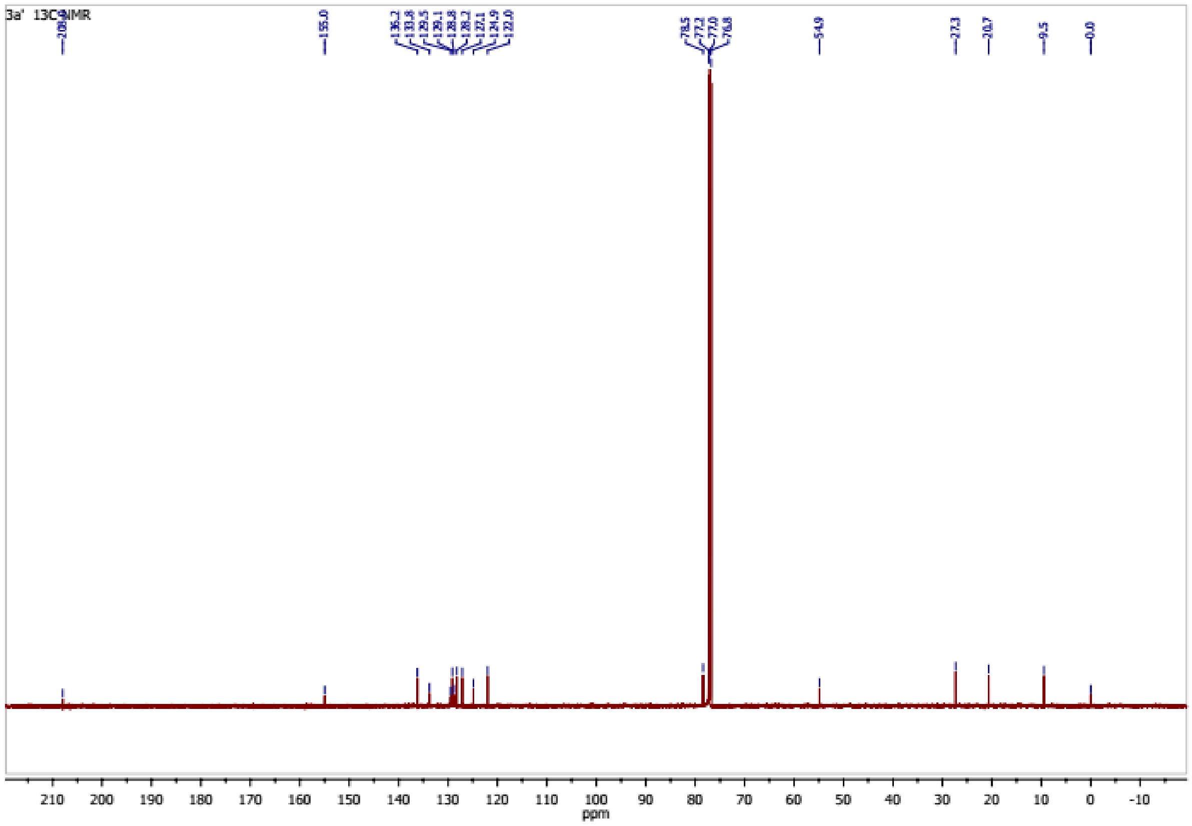

Figure S6. ^{13}C NMR spectrum of **3a′**.

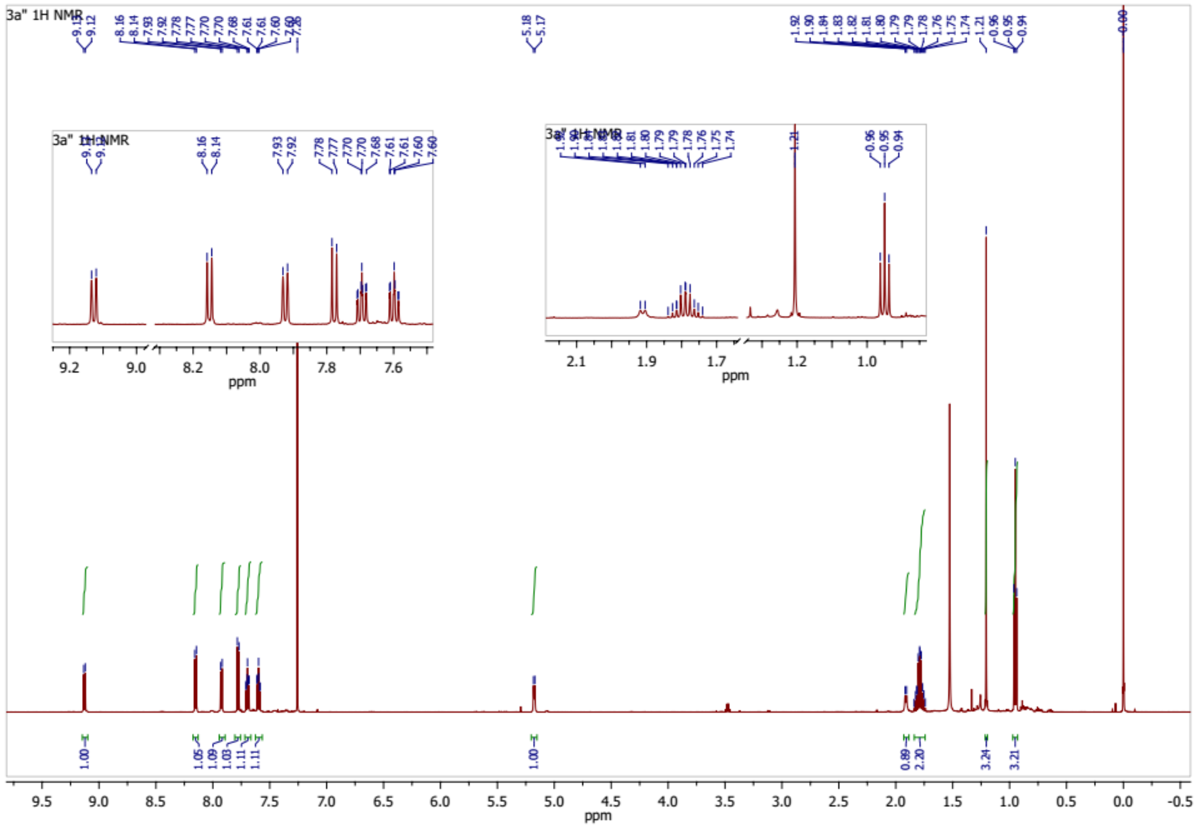

Figure S7. ^{1}H NMR spectrum of **3a′′**.

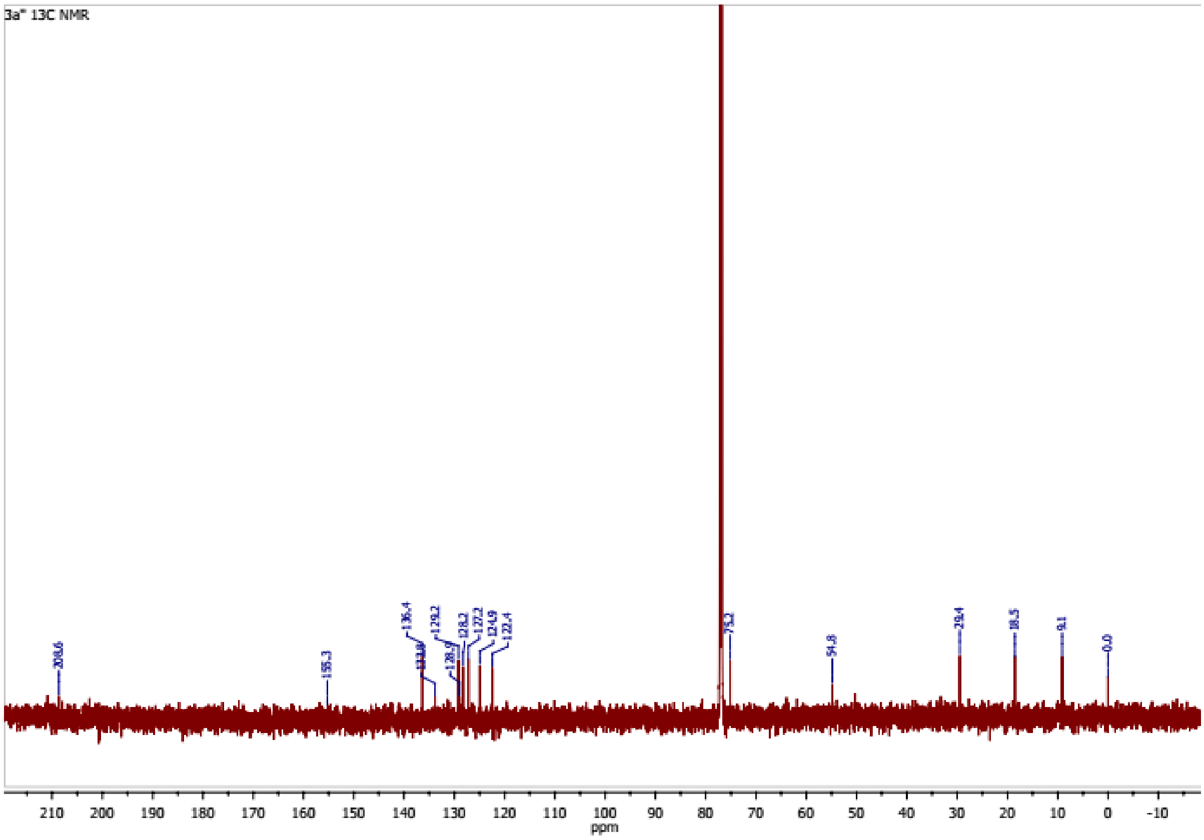

Figure S8. ^{13}C NMR spectrum of **3a′′**.

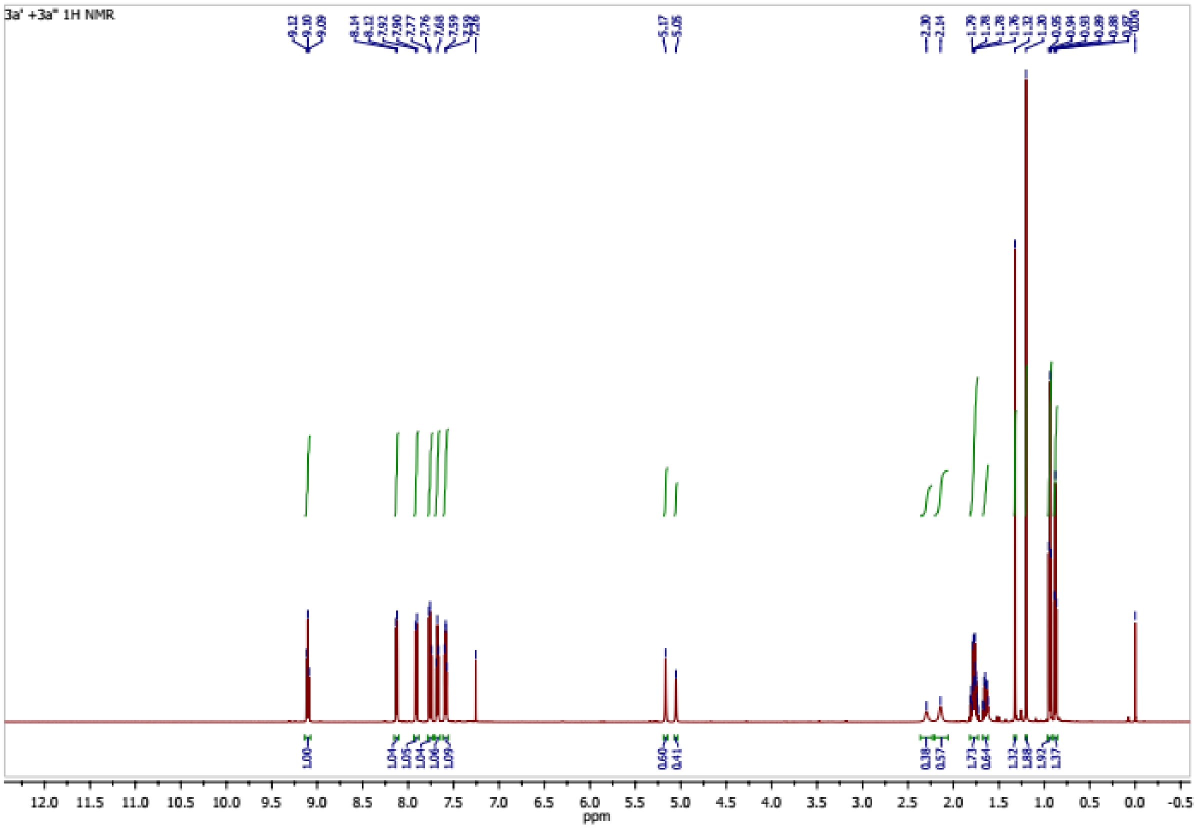

Figure S9. ^{1}H NMR spectrum of **3a′+3a′′**.

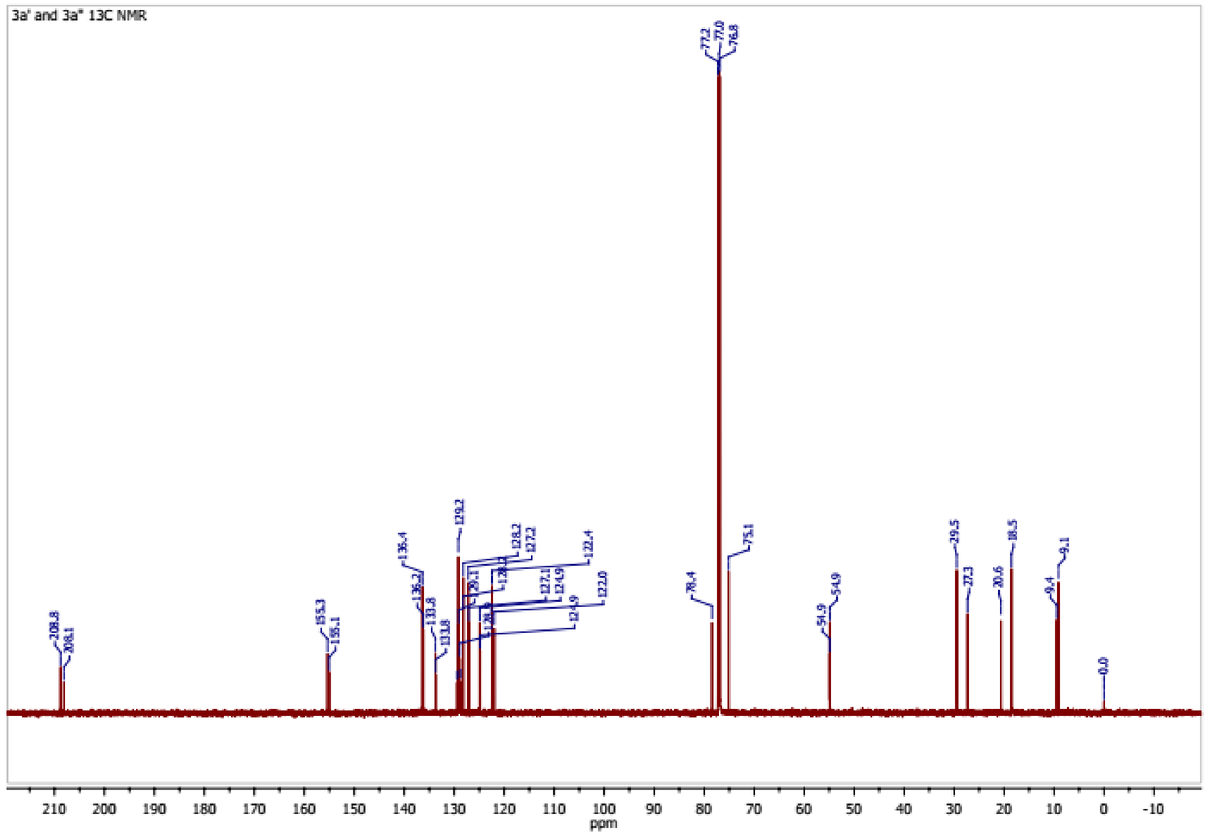

Figure S10. ^{13}C NMR spectrum of **3a′+3a″**.

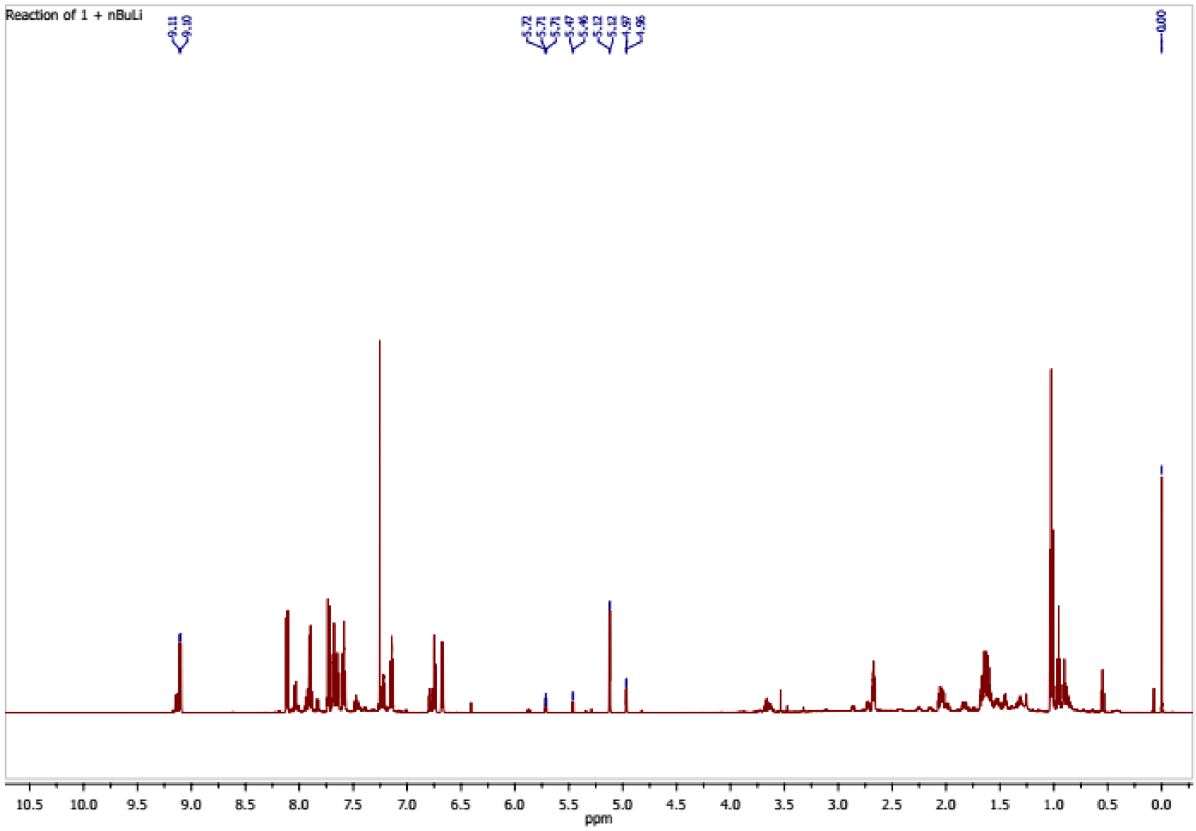

Figure S11. ^{1}H NMR spectrum of **1+*n*BuLi**.

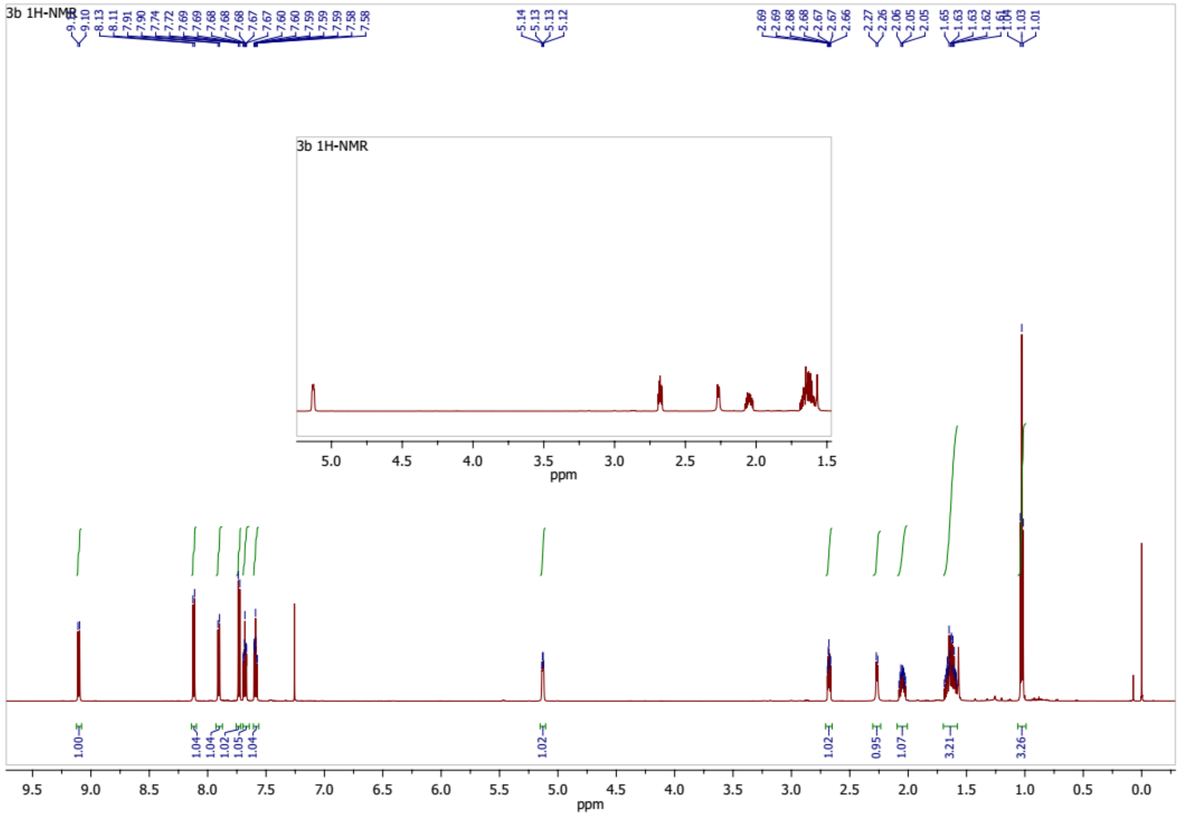

Figure S12. ^{1}H NMR spectrum of **3b**.

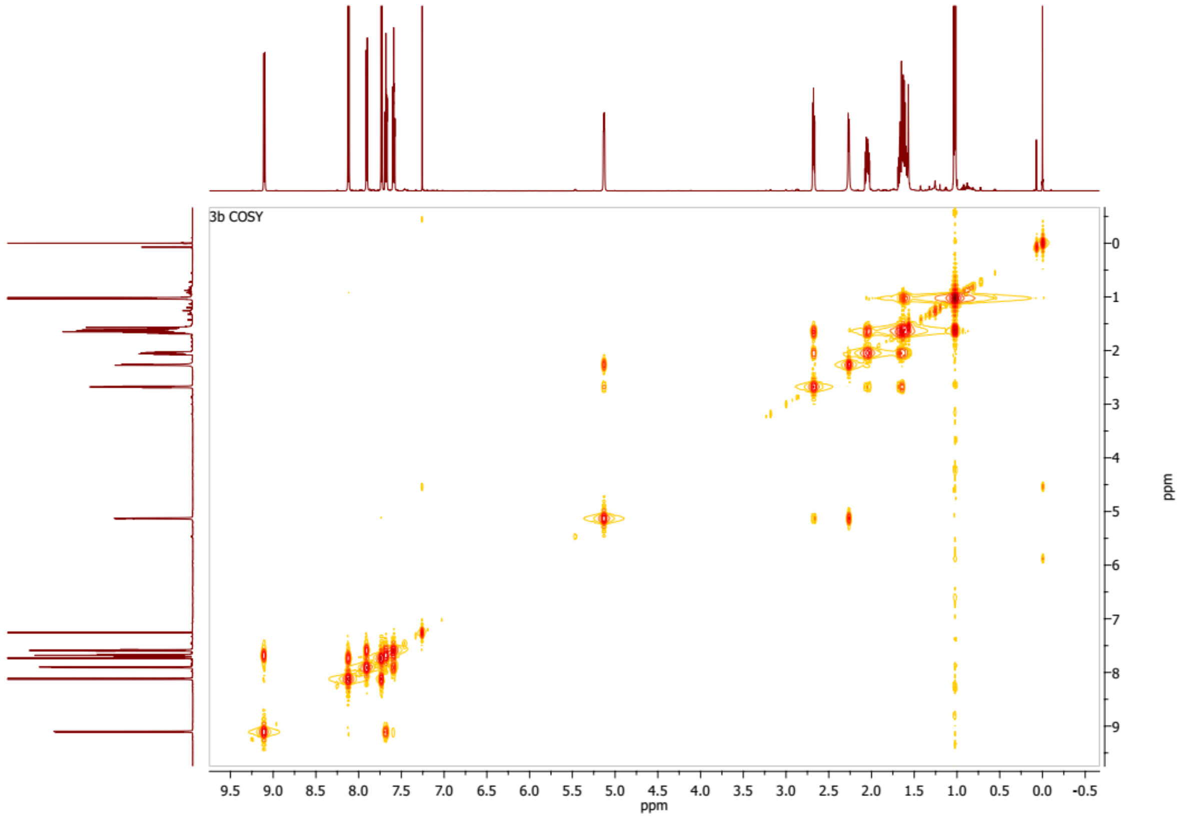

Figure S13. ${}^{1}H,{}^{1}H$ COSY NMR spectrum of **3b** (expansion).

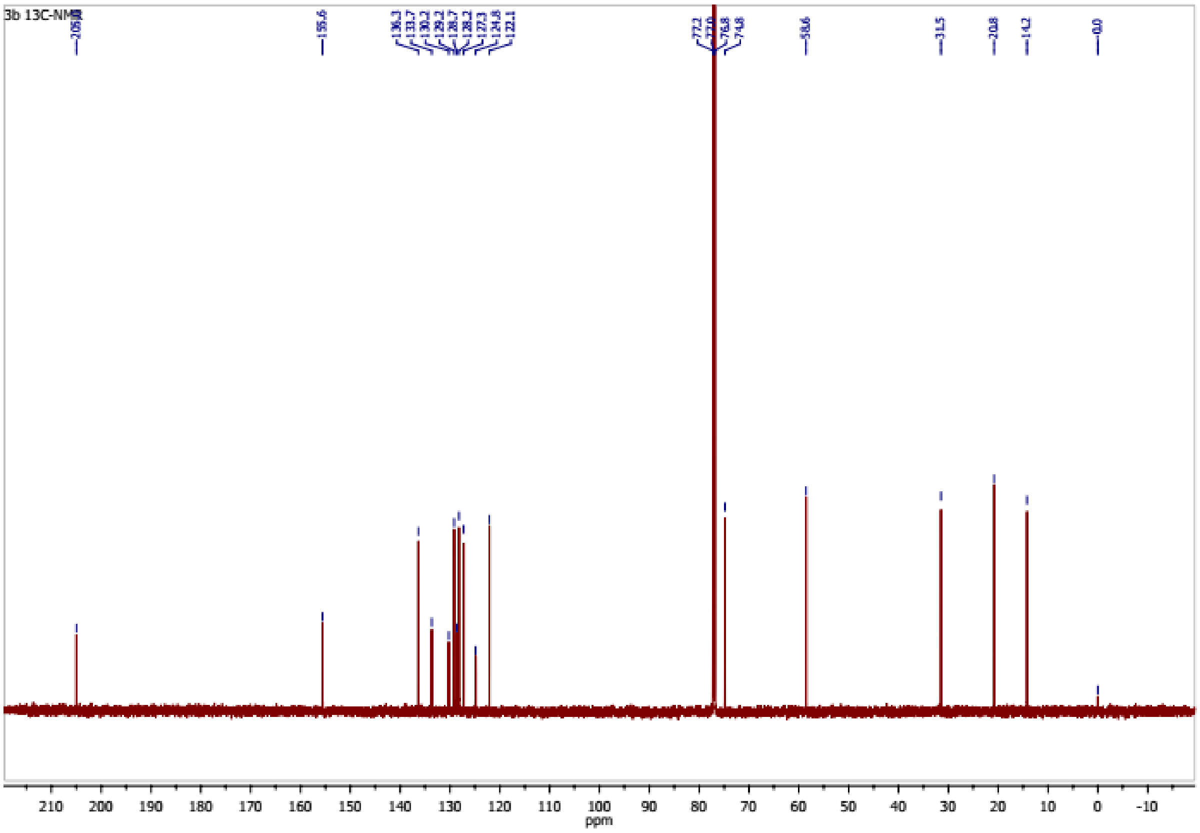

Figure 14. ${}^{13}C$ NMR spectrum of **3b**.

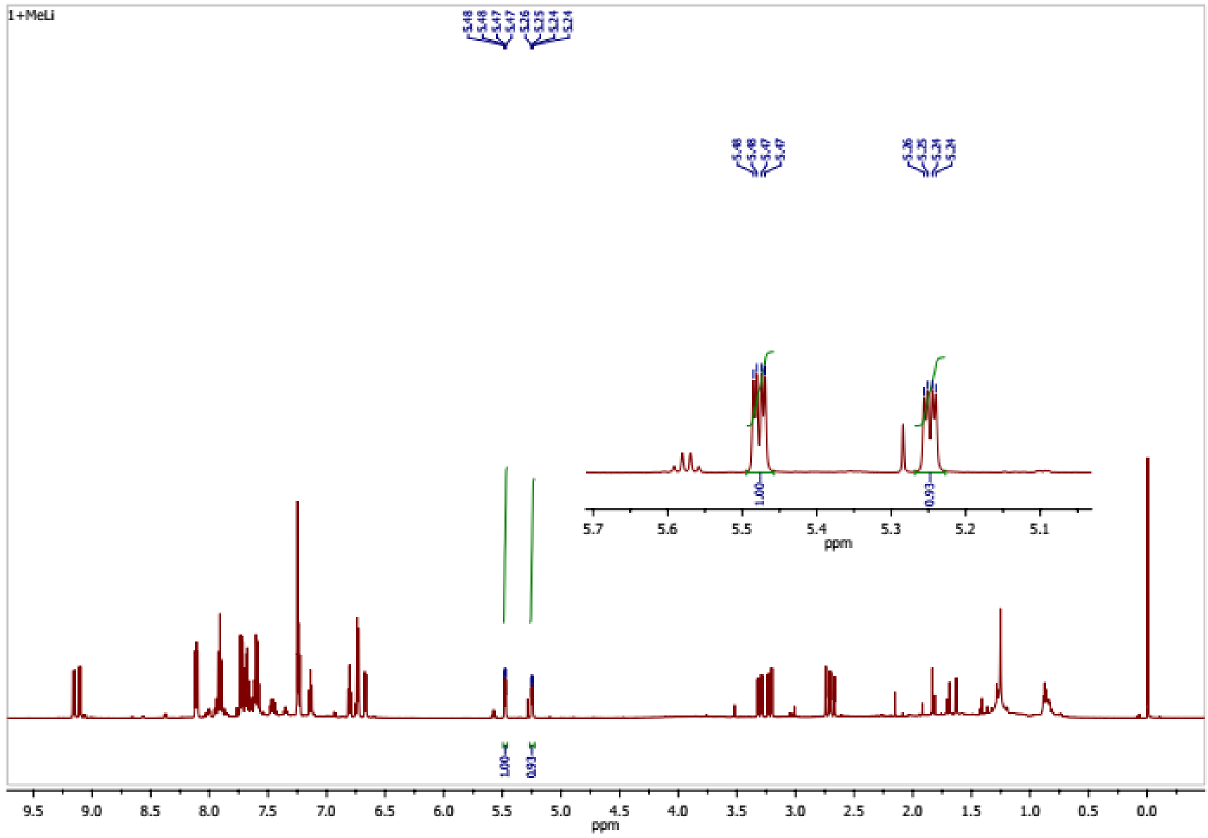

Figure S15. ^{1}H NMR spectrum of **1+MeLi**.

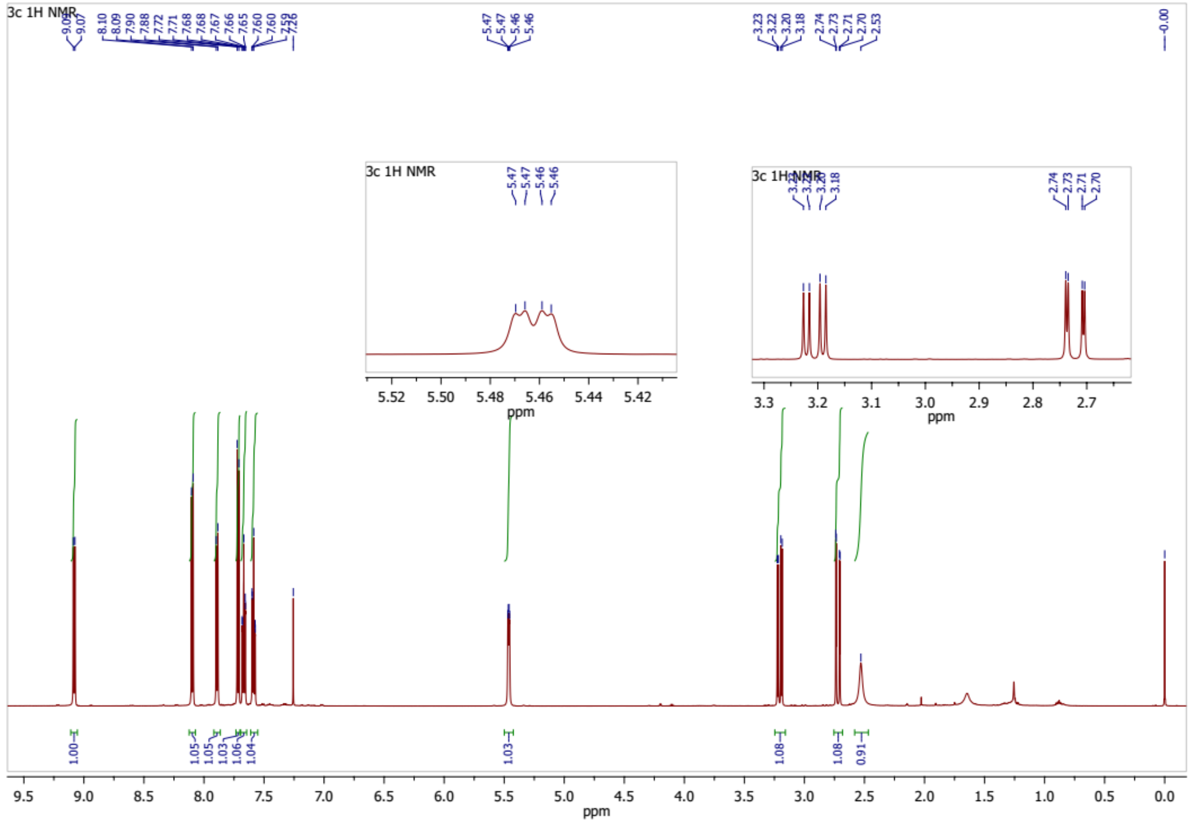

Figure S16. ^{1}H NMR spectrum of **3c**.

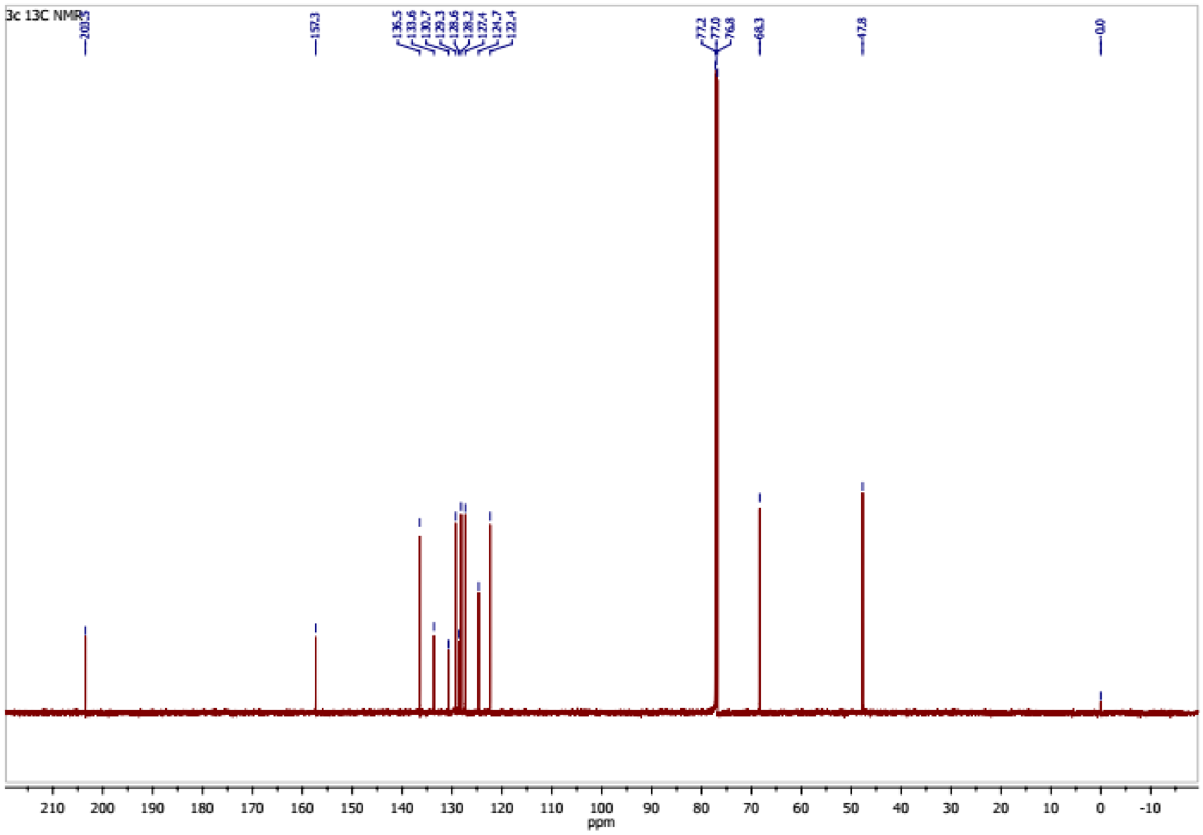

Figure S17. ^{13}C NMR spectrum of **3c**.

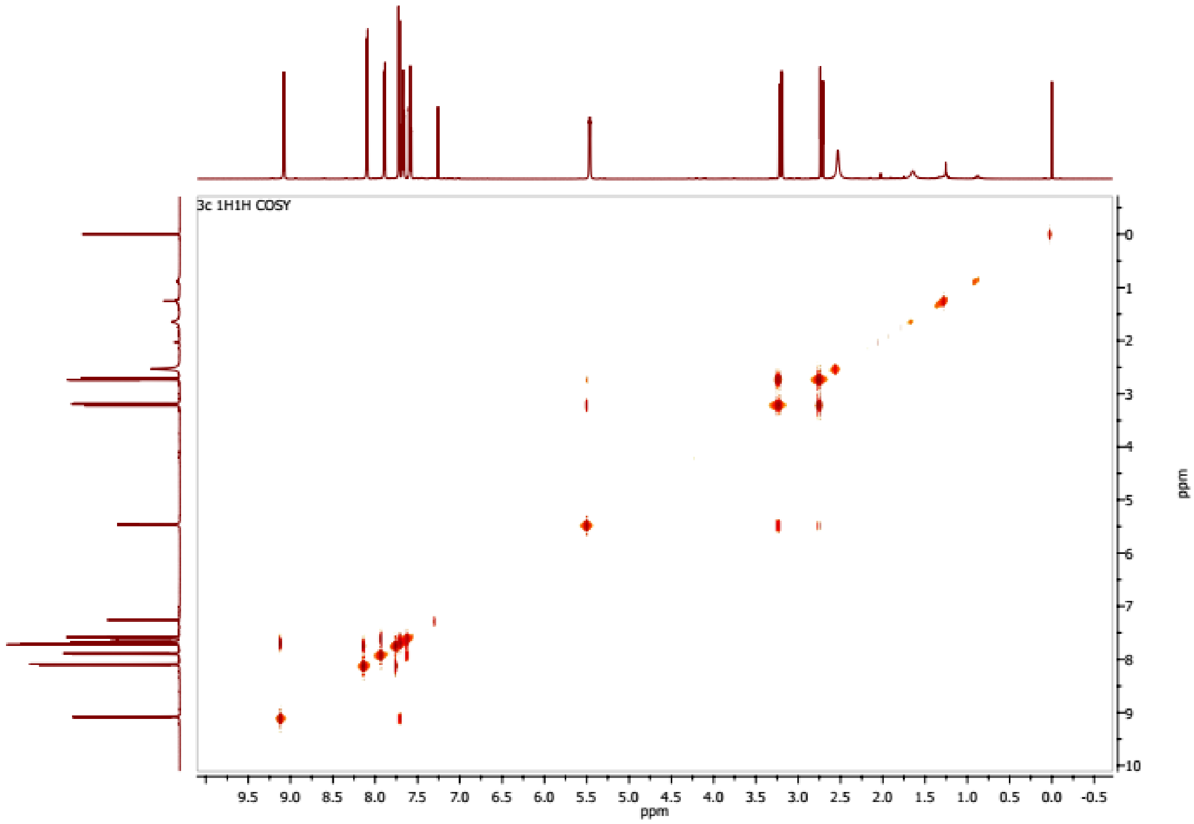

Figure S18. $^{1}H,^{1}H$ COSY NMR spectrum of **3c**.

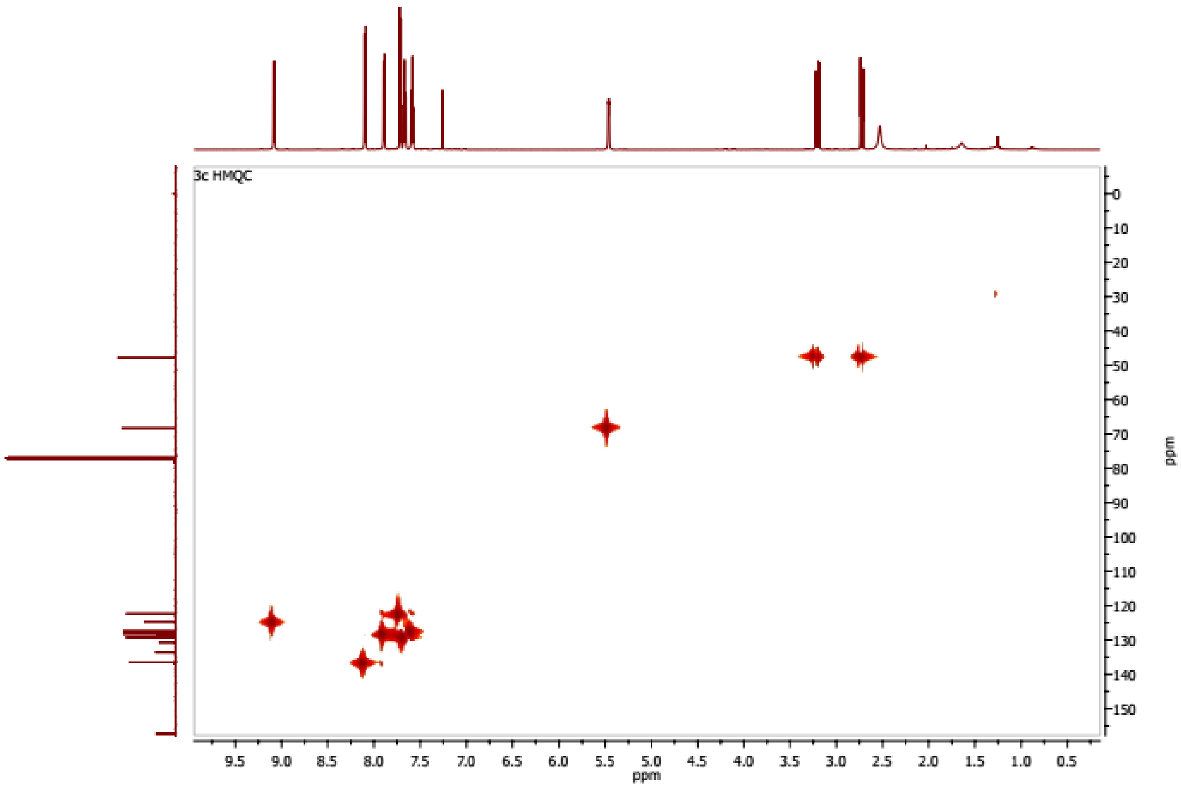

Figure S19. ^{1}H,^{13}C HMQC NMR spectrum of **3c**.

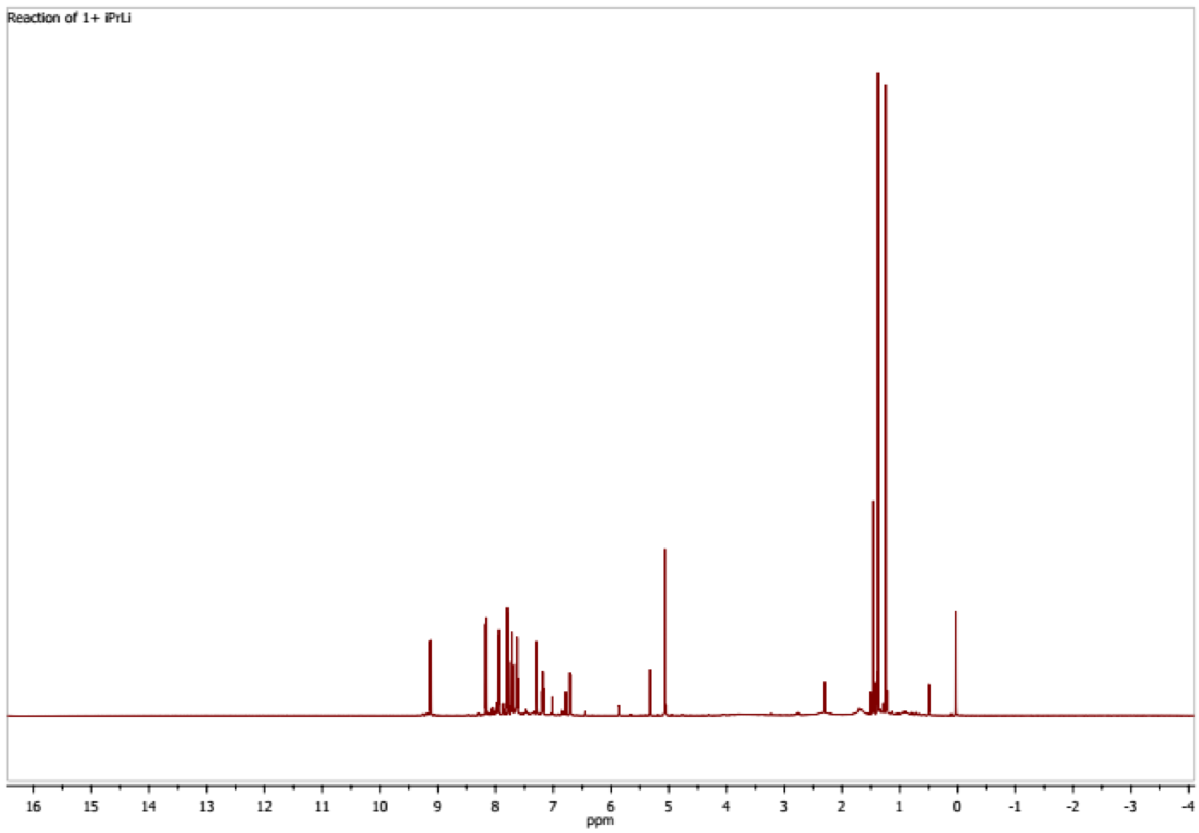

Figure S20. ^{1}H NMR spectrum of **1+*i*PrLi**.

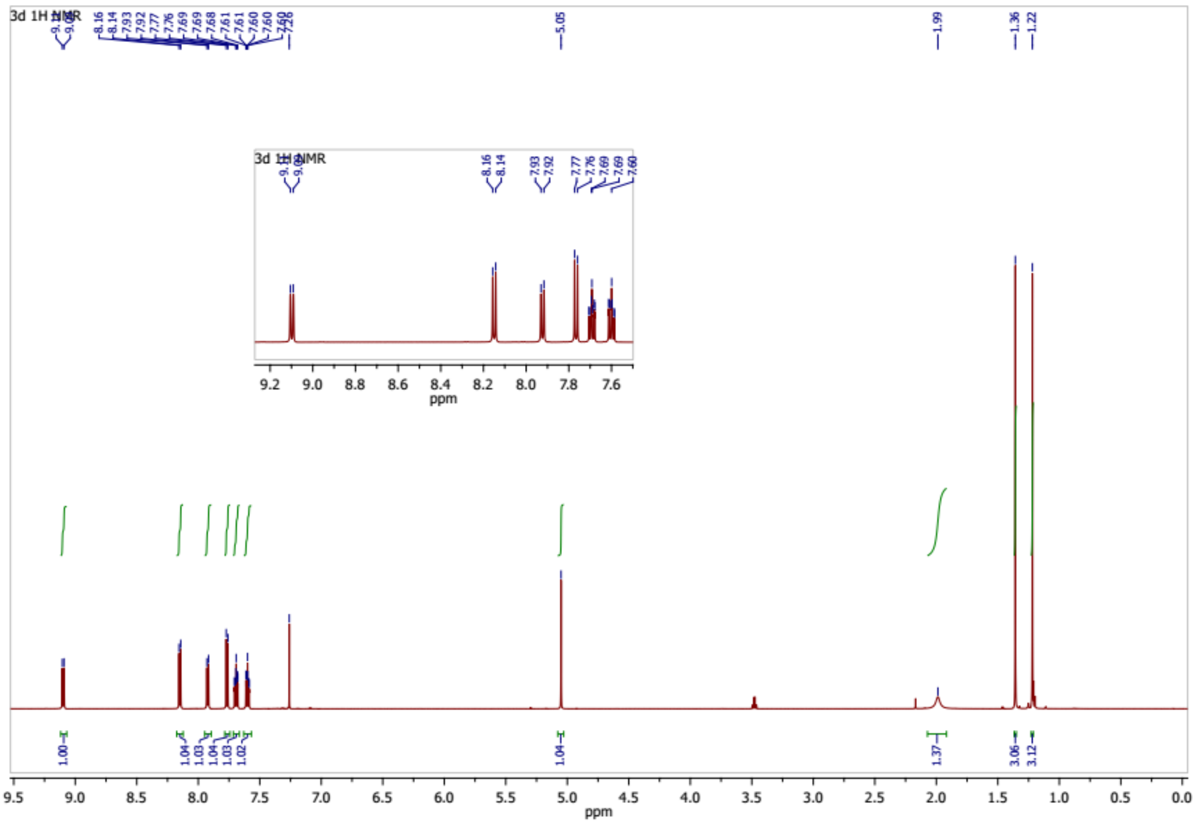

Figure S21. ^{1}H NMR spectrum of **3d**.

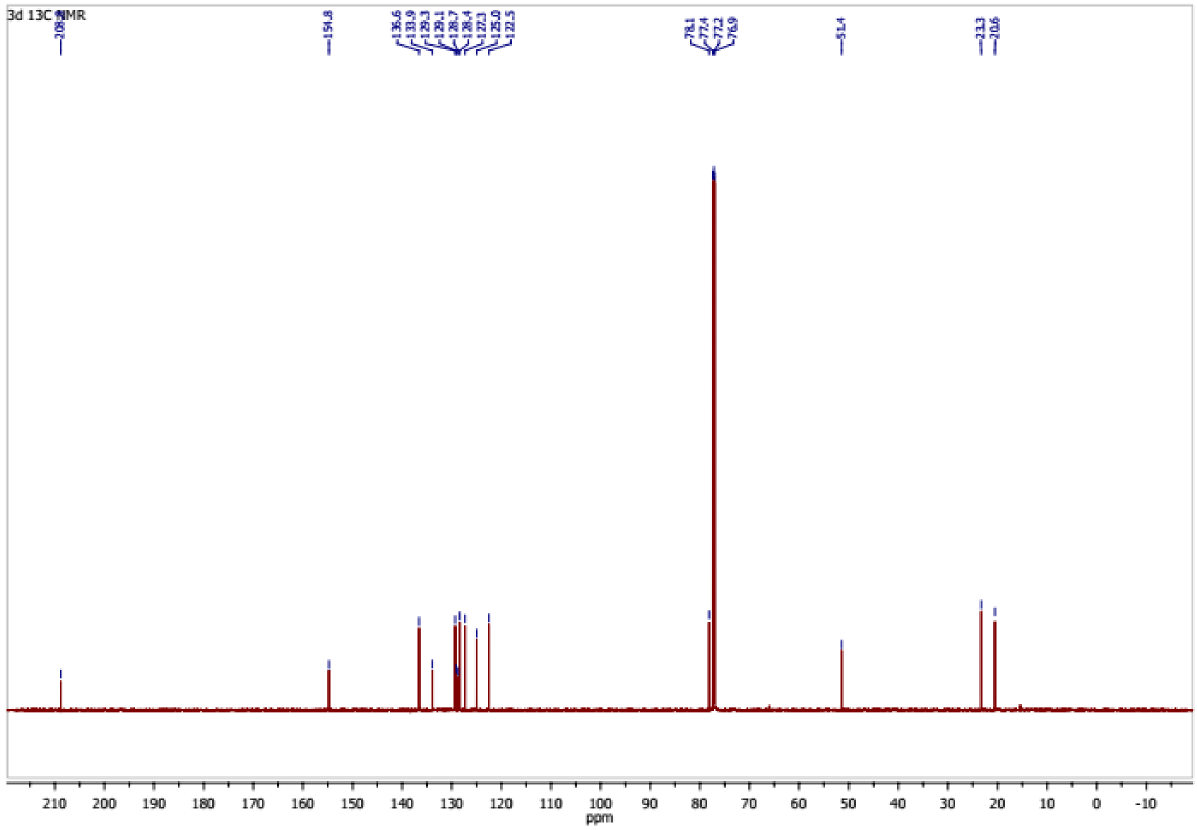

Figure S22. ^{13}C NMR spectrum of **3d**.

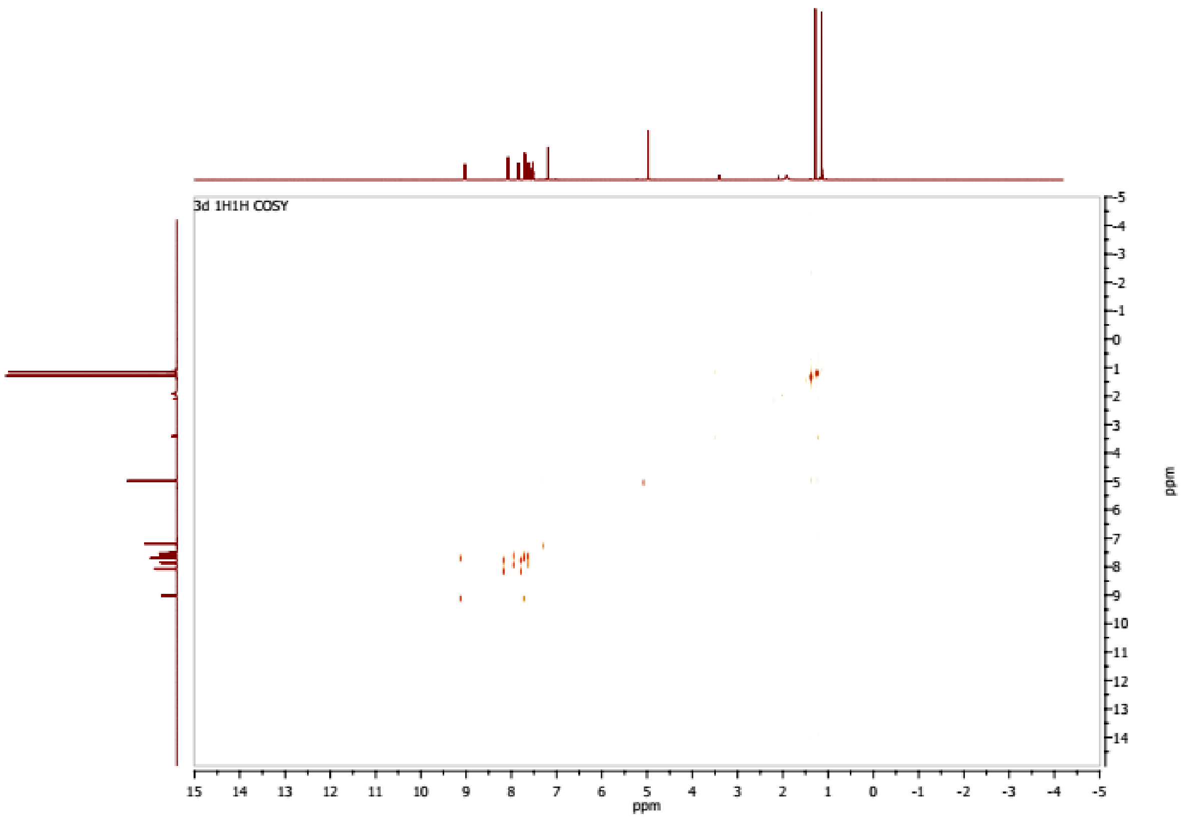

Figure S23. ^{1}H, ^{1}H COSY NMR spectrum of **3d**.

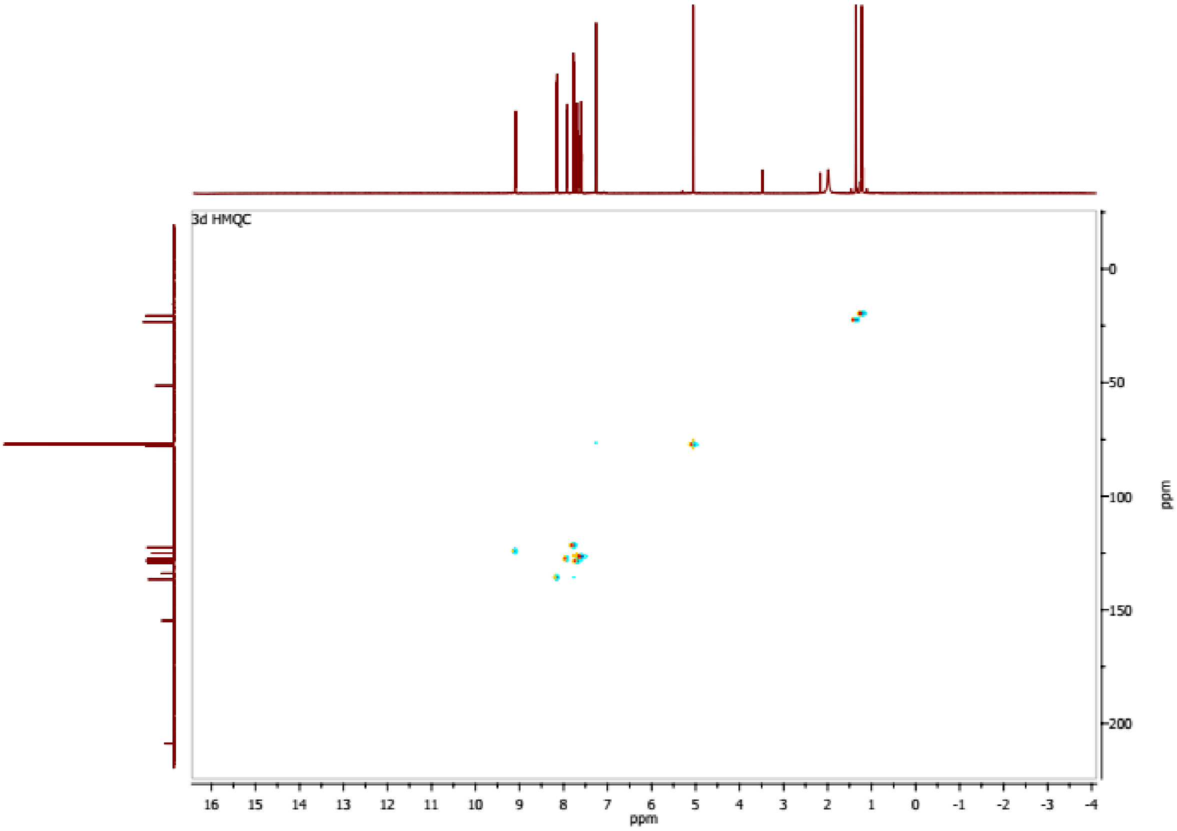

Figure S24. $^{1}H,^{13}C$ HMQC NMR spectrum of **3d**

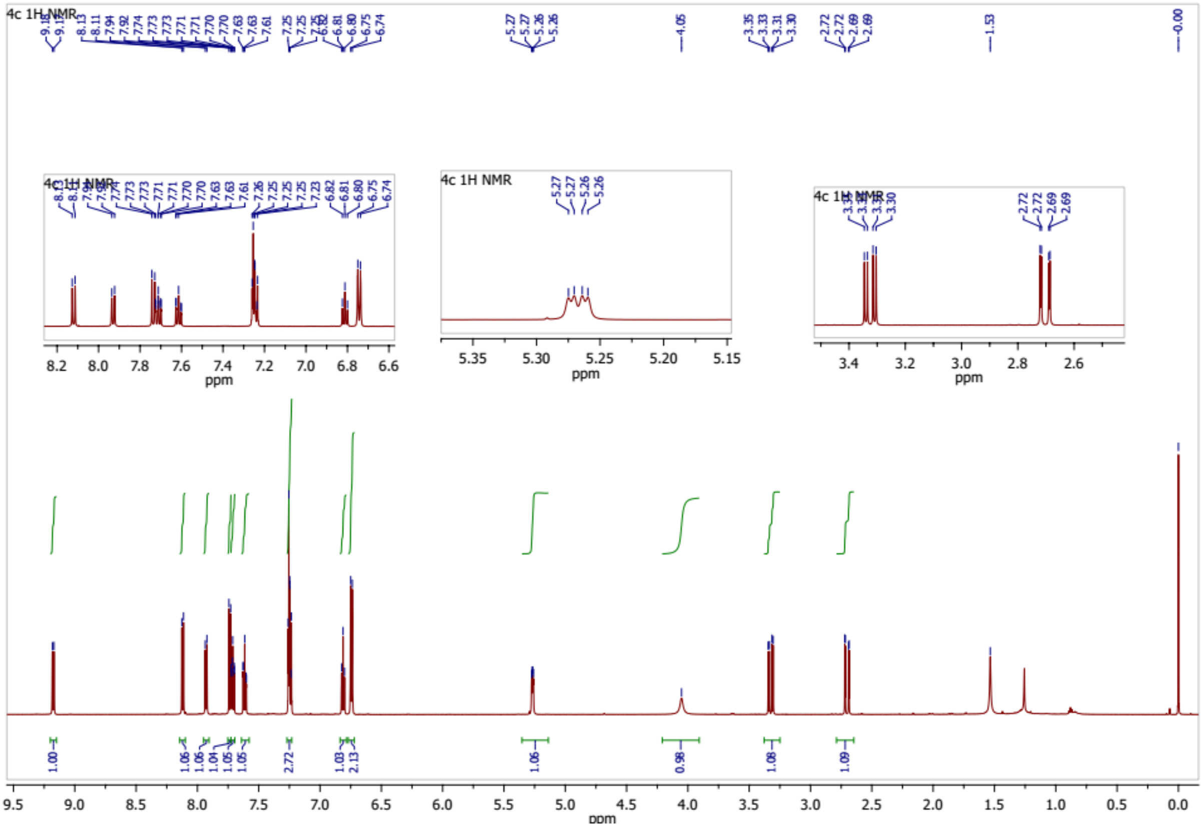

Figure S25. ^{1}H NMR spectrum of **4c**.

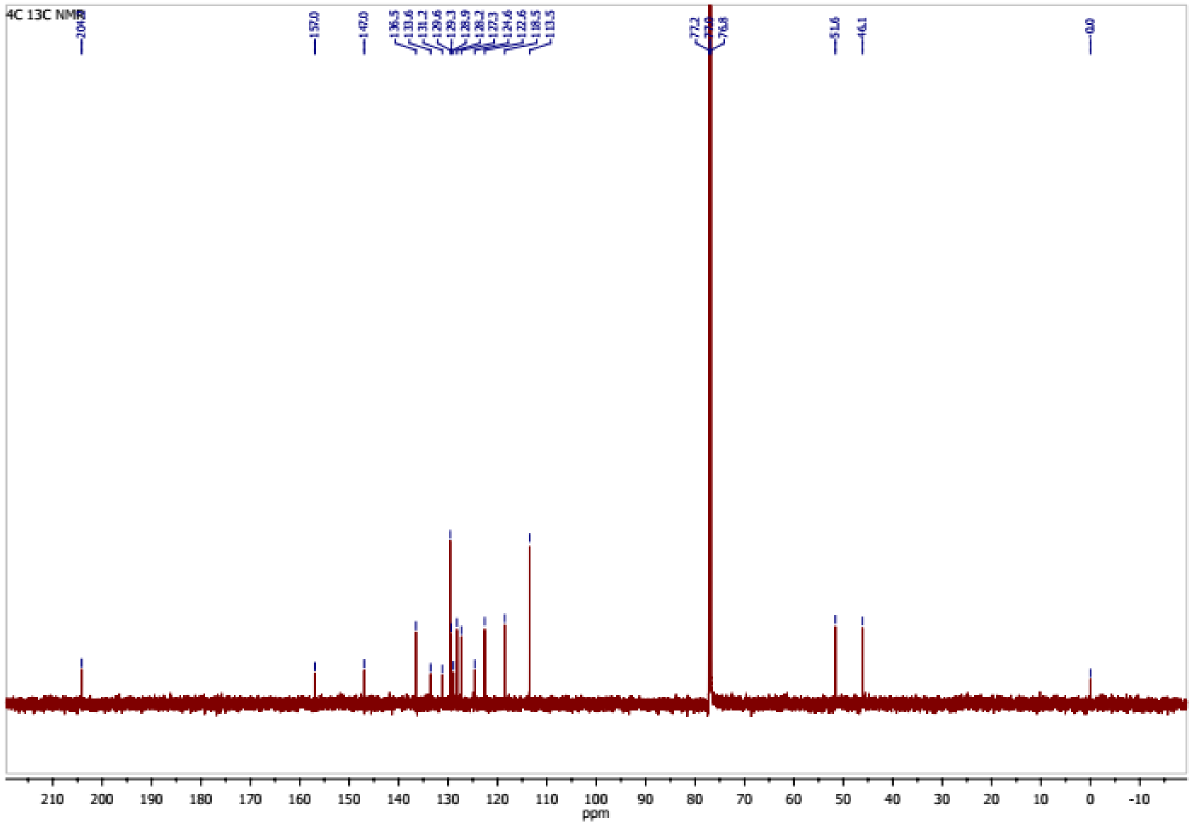

Figure S26. ^{13}C NMR spectrum of **4c**.

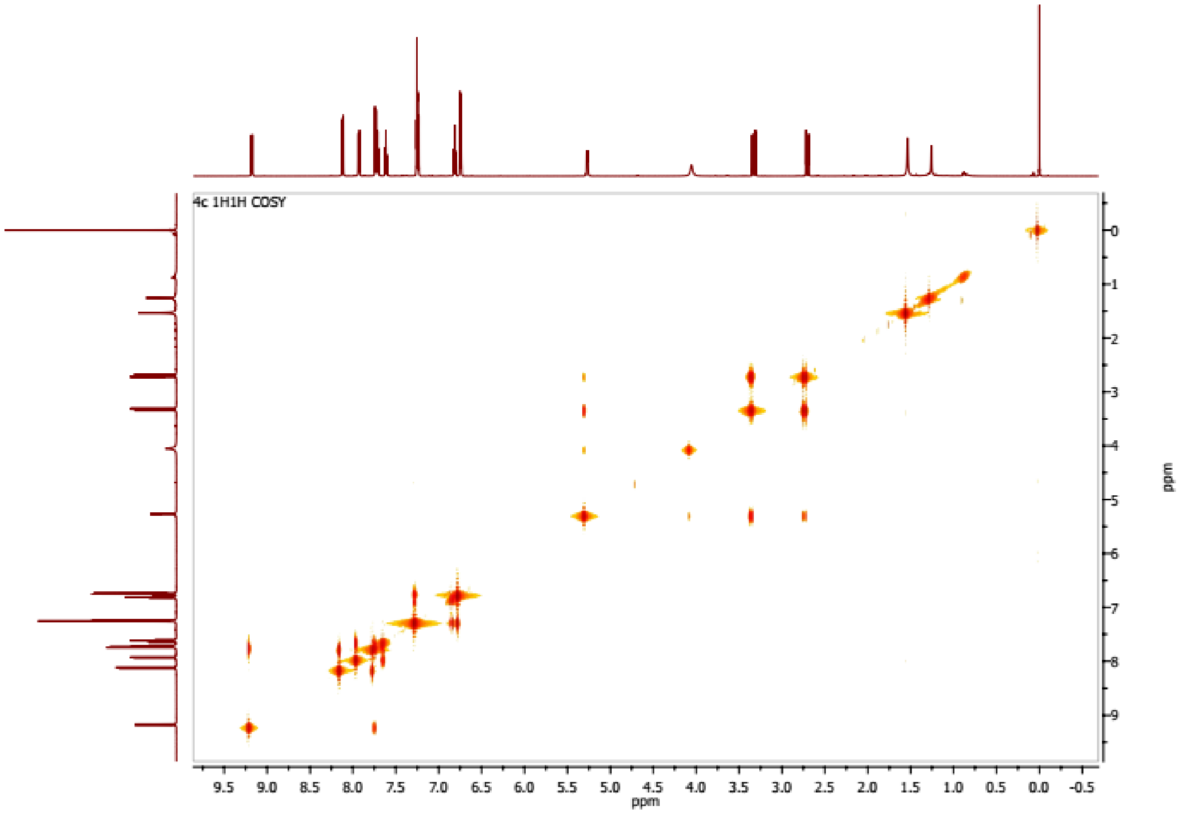

Figure S27. ^{1}H, ^{1}H COSY NMR spectrum of **4c**.

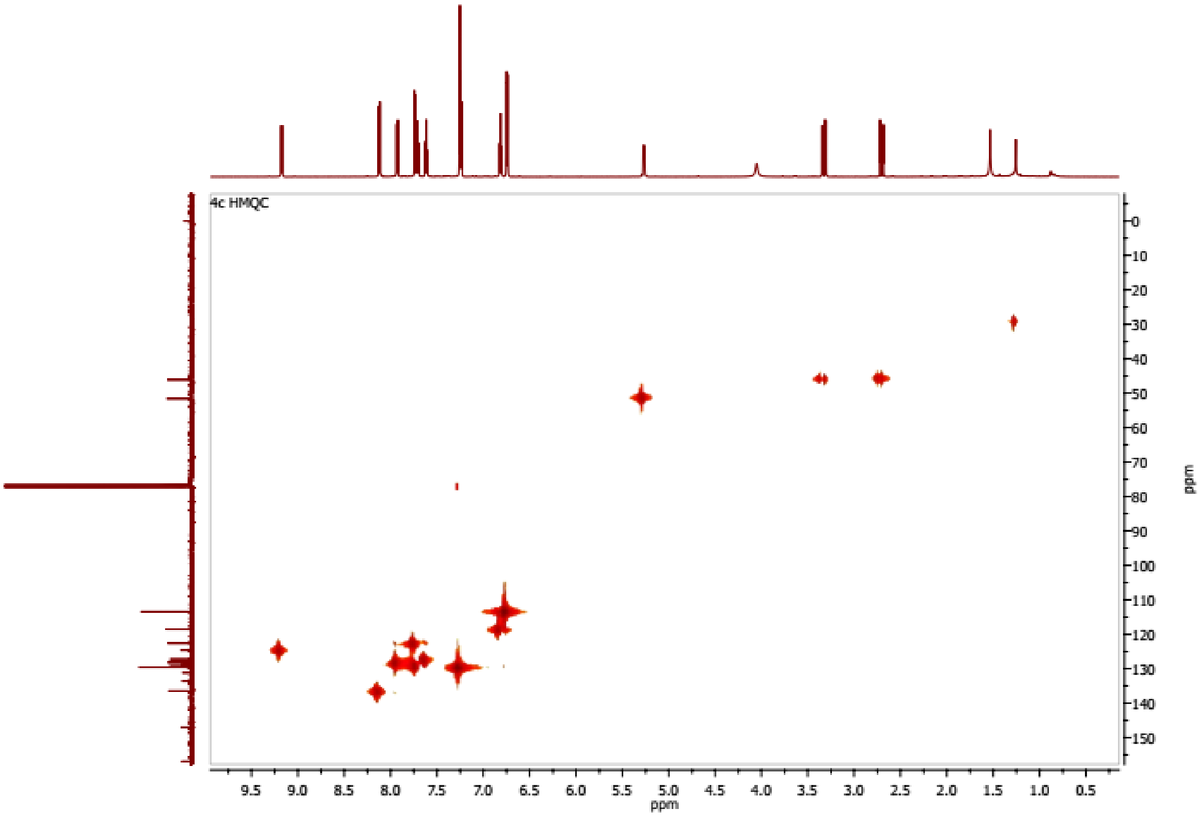

Figure S28. ^{1}H, ^{13}C HMQC NMR spectrum of **4c**.

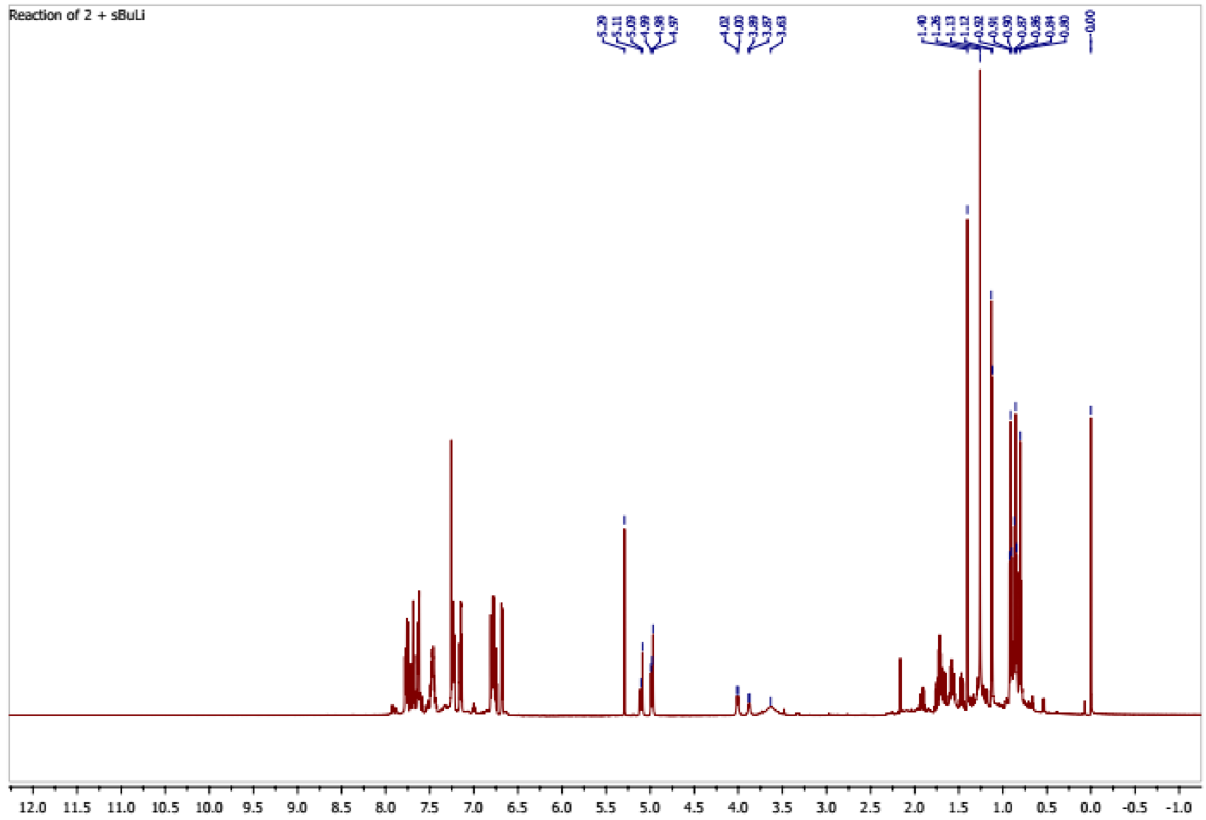

Figure S29. ^{1}H NMR spectrum of **2+*s*BuLi**.

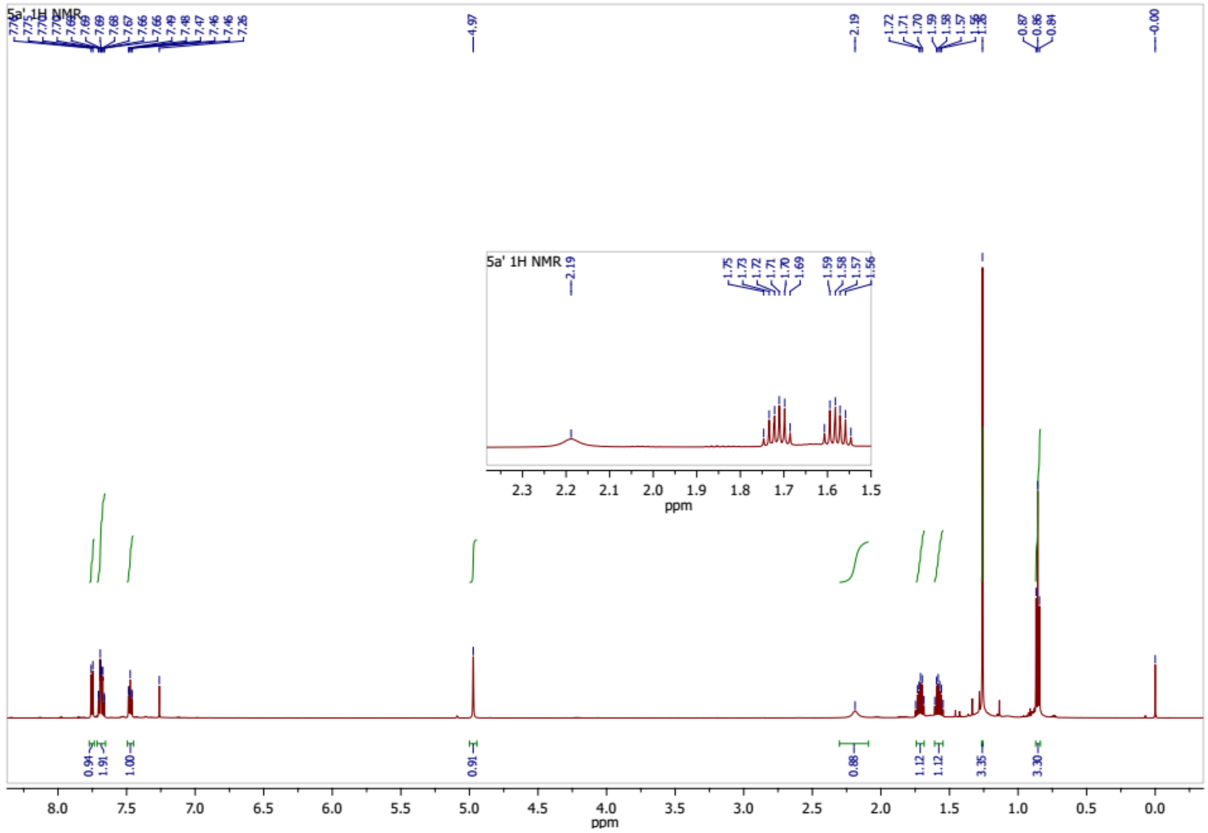

Figure S30. ^{1}H NMR spectrum of **5a'**.

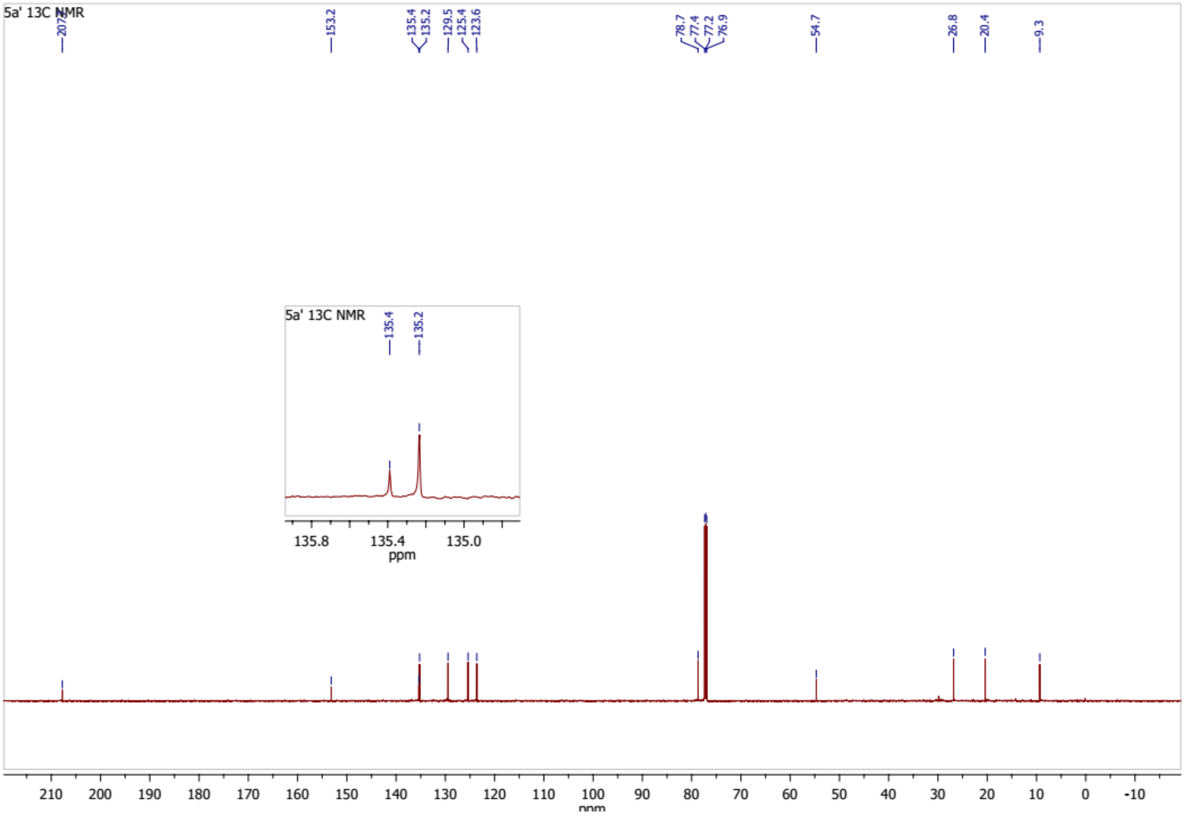

Figure S31. ^{13}C NMR spectrum of **5a'**.

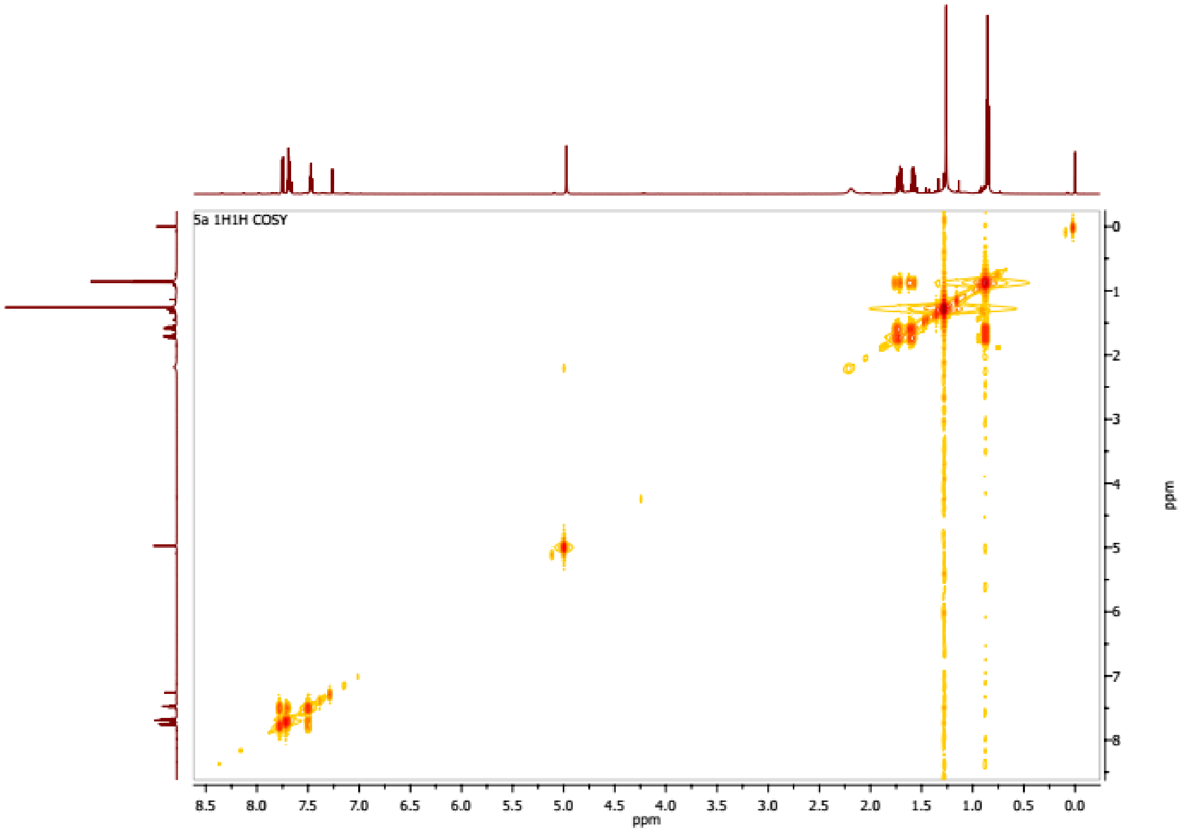

Figure S32. ^{1}H, ^{1}H COSY NMR spectrum of **5a'**.

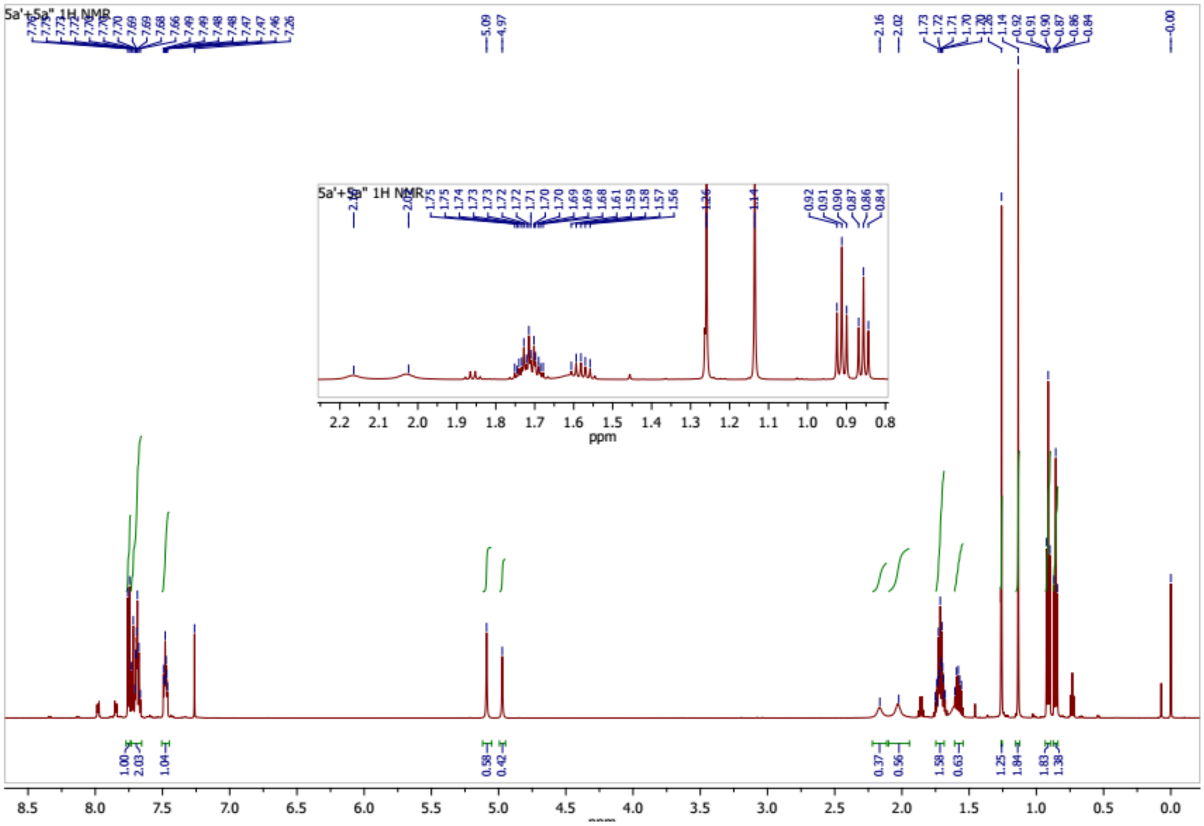

Figure S33. ^{1}H NMR spectrum of **5a′+5a′′**.

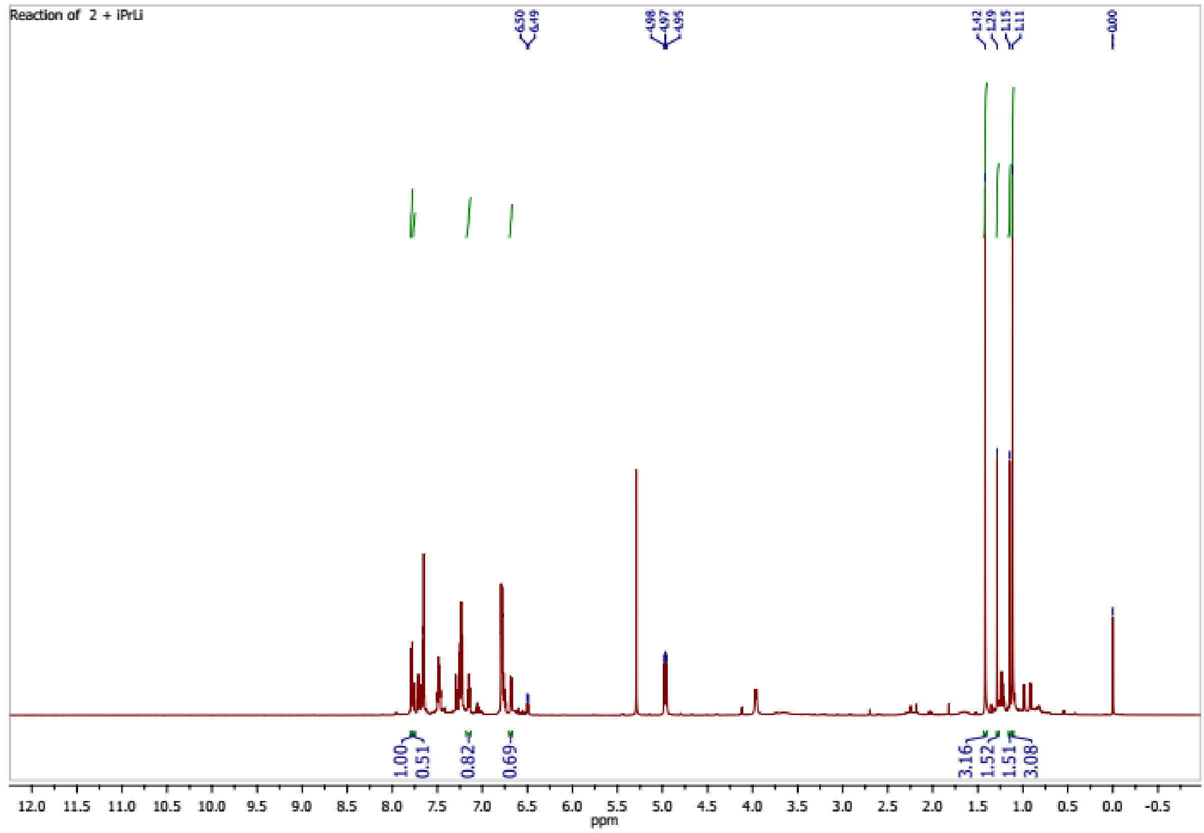

Figure S34. ^{1}H NMR spectrum of **2** + ***i*****PrLi**

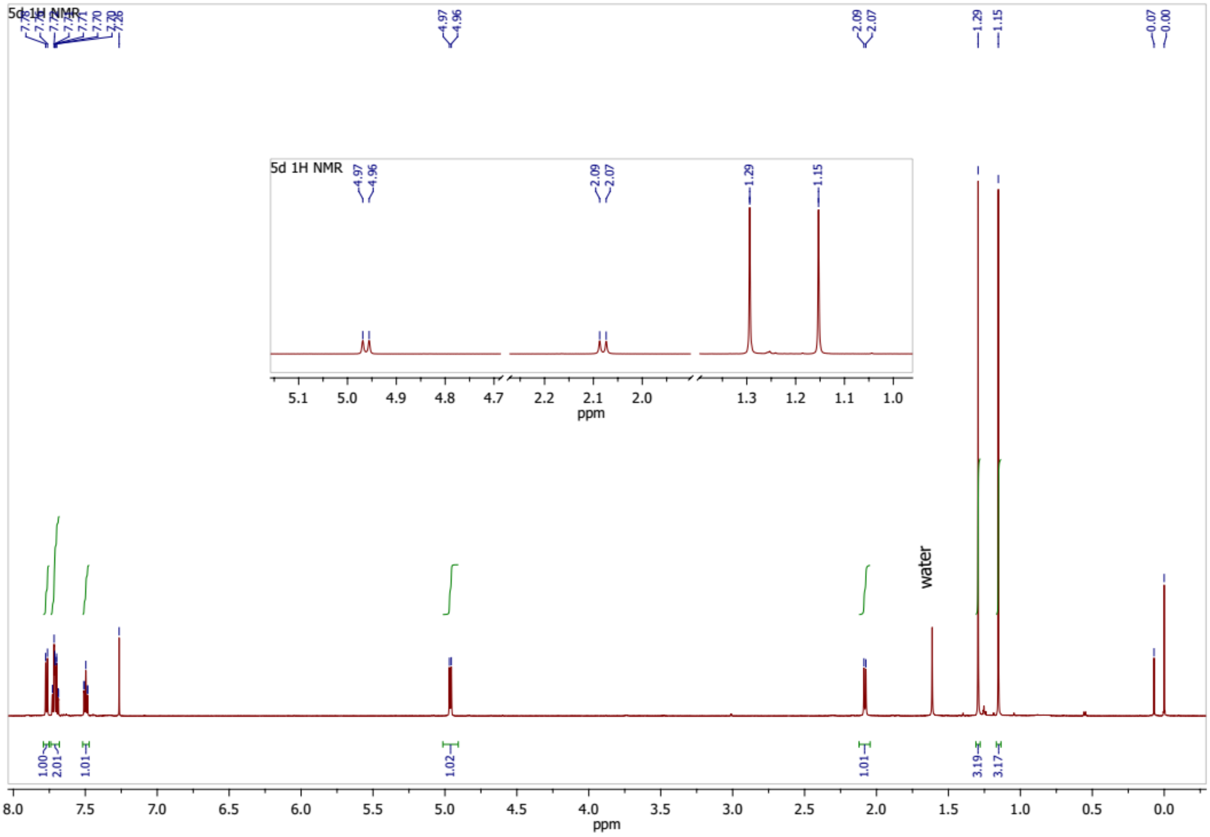

Figure S35. ^{1}H NMR spectrum of **5d**.

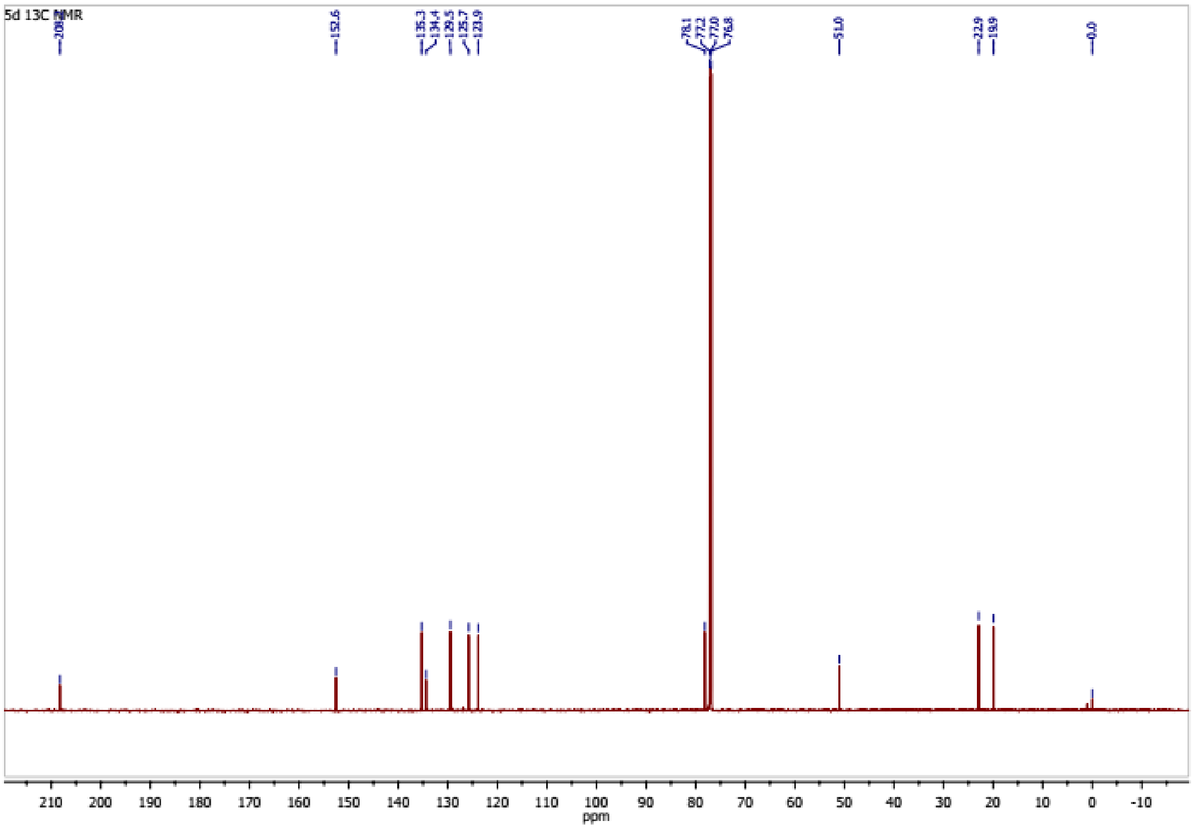

Figure S36. ^{13}C NMR spectrum of **5d**.

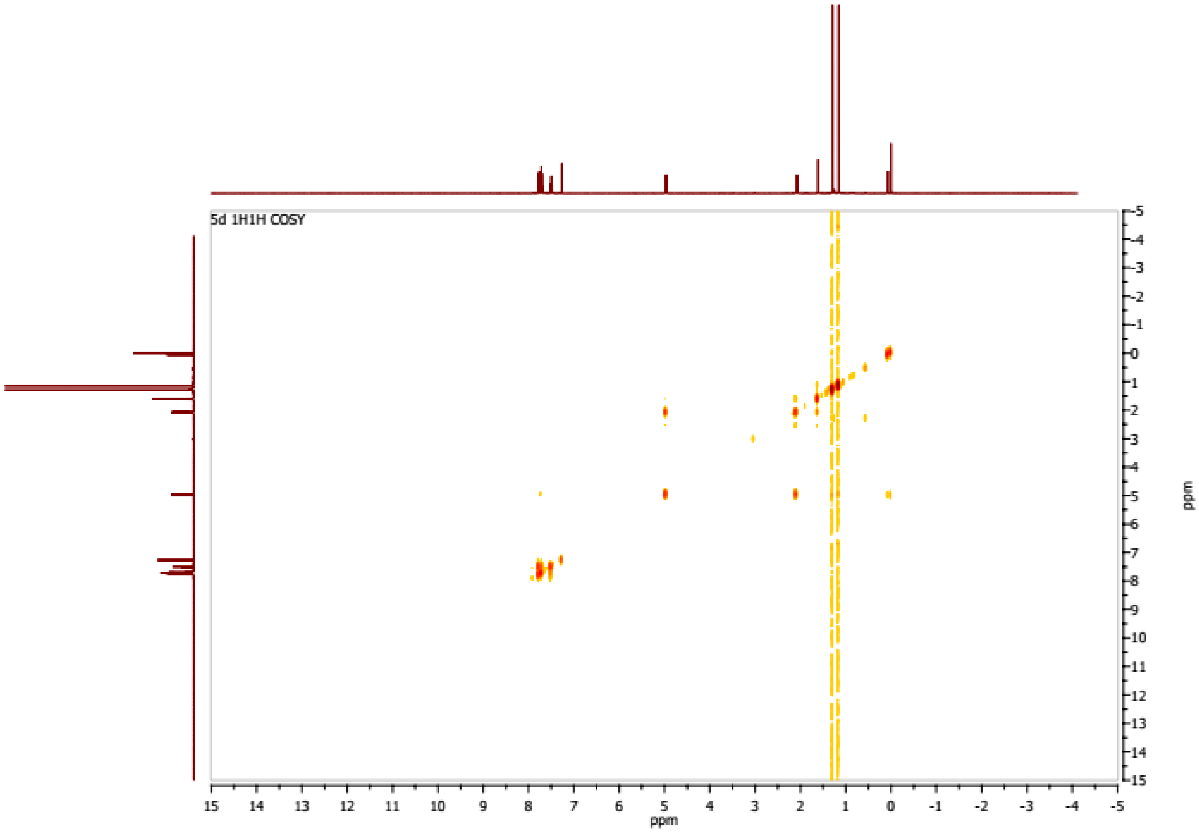

Figure S37. ^{1}H, ^{1}H COSY NMR spectrum of **5d**.

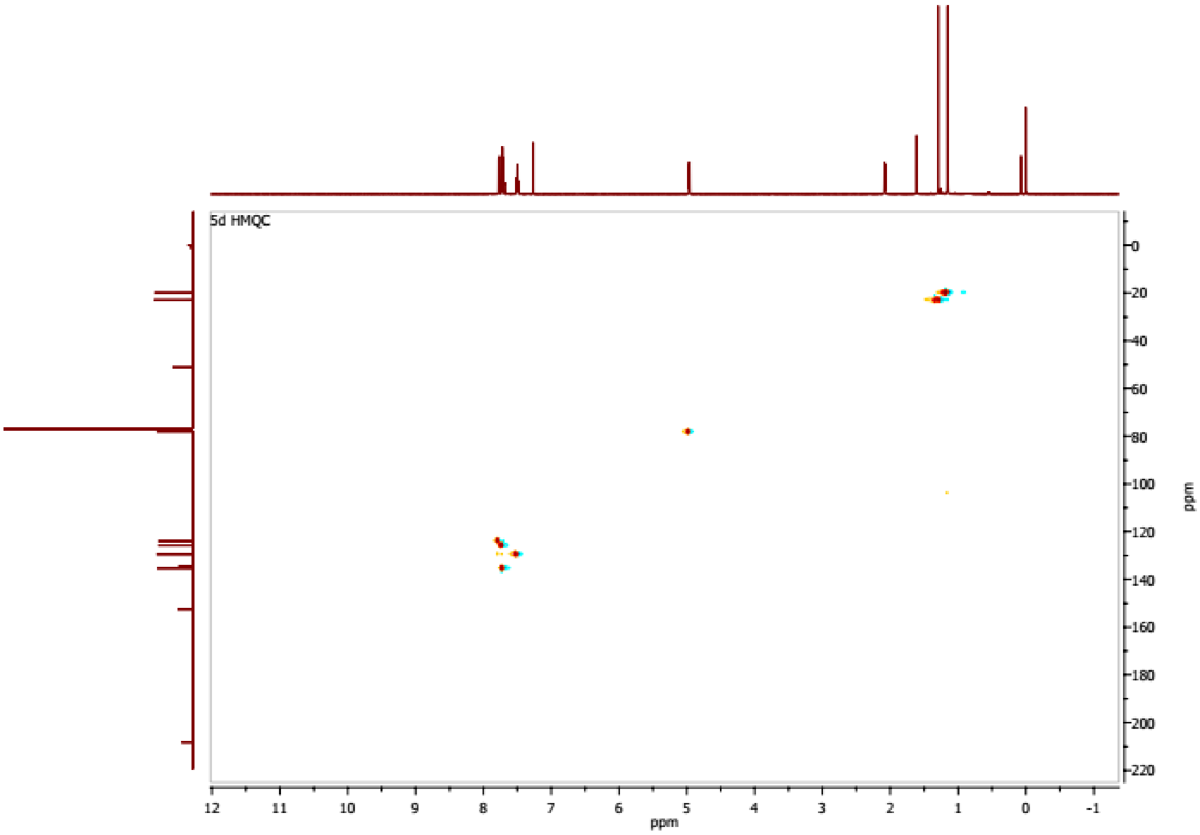

Figure S38. ^{1}H, ^{13}C HMQC NMR spectrum of **5d**.

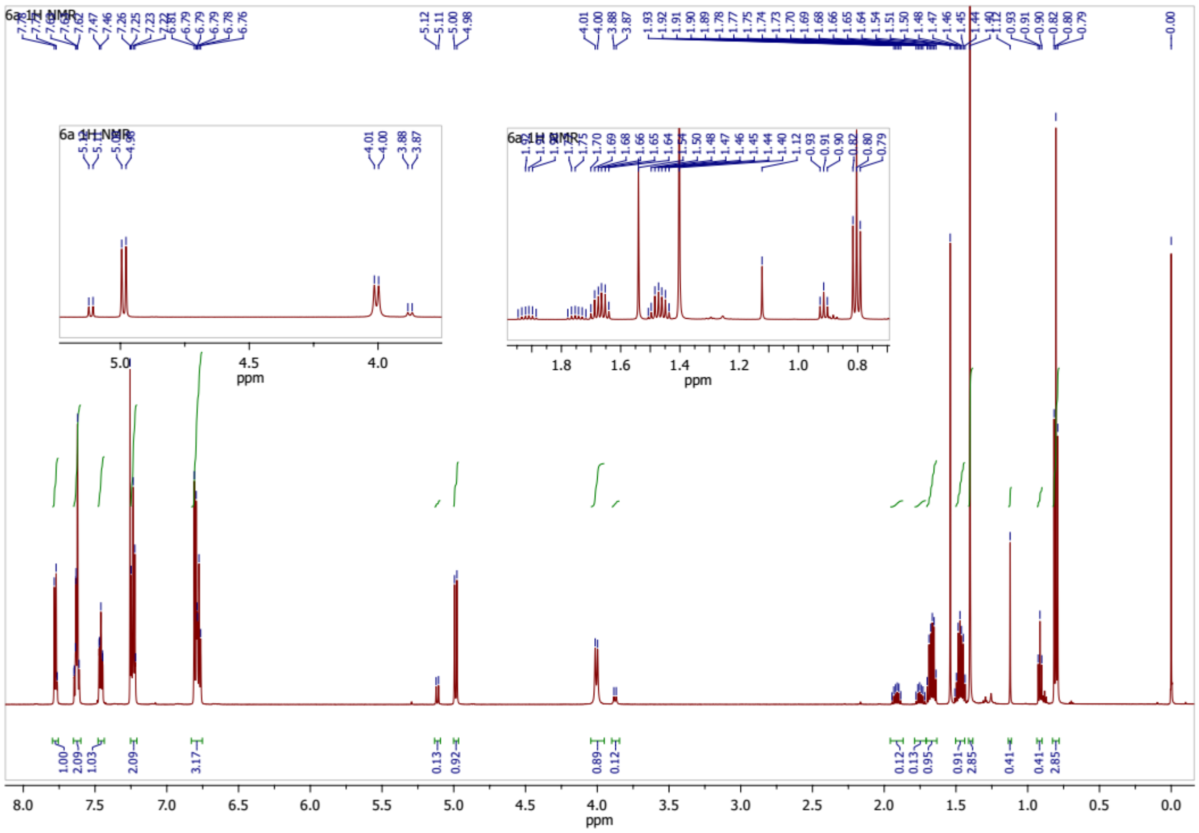

Figure S39. ^{1}H NMR spectrum of **6a**.

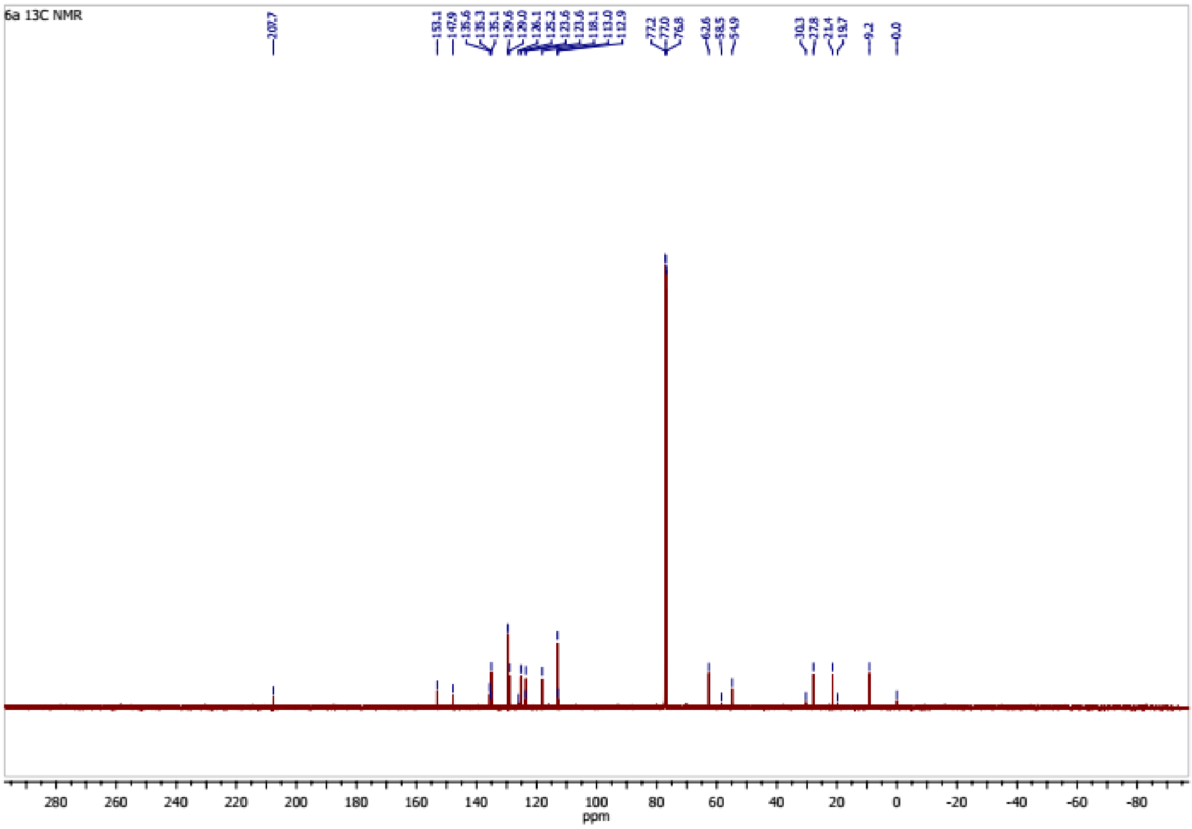

Figure S40. ^{13}C NMR spectrum of **6a**.

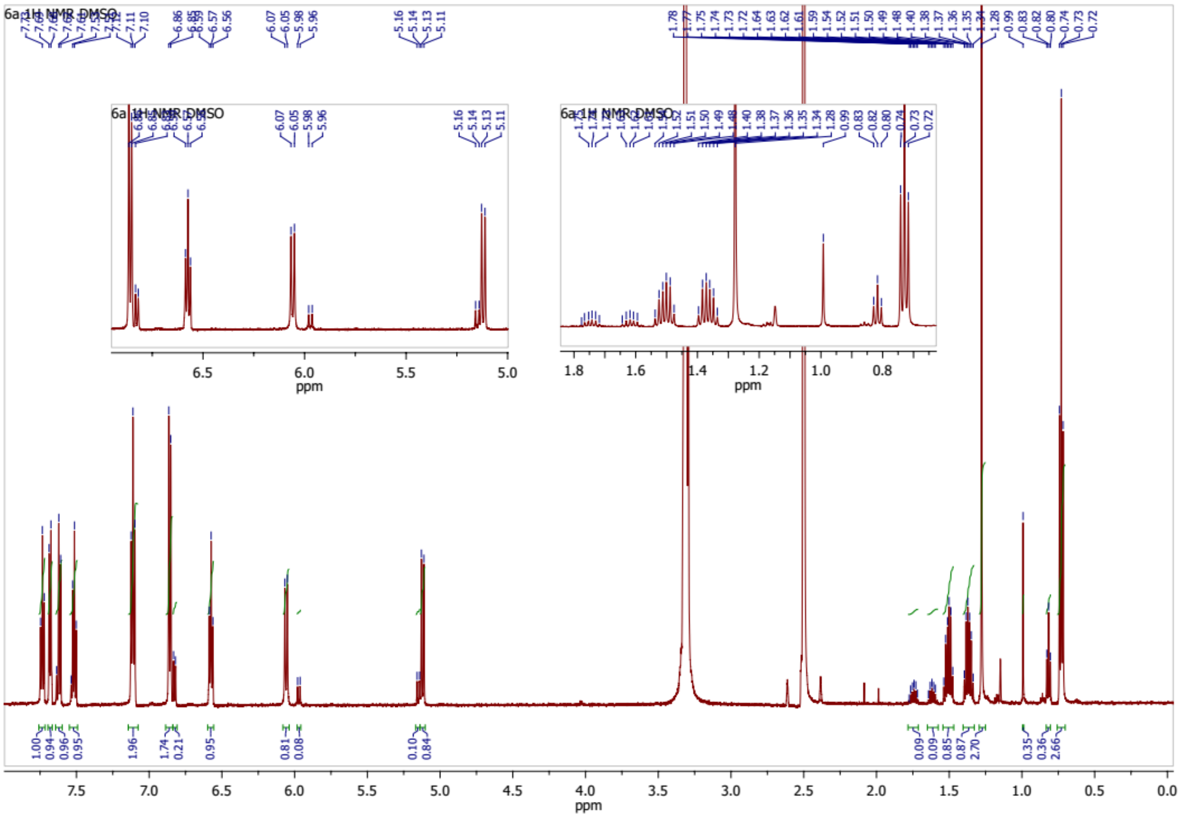

Figure S41. ^{1}H NMR spectrum of **6a** (DMSO-d_6).

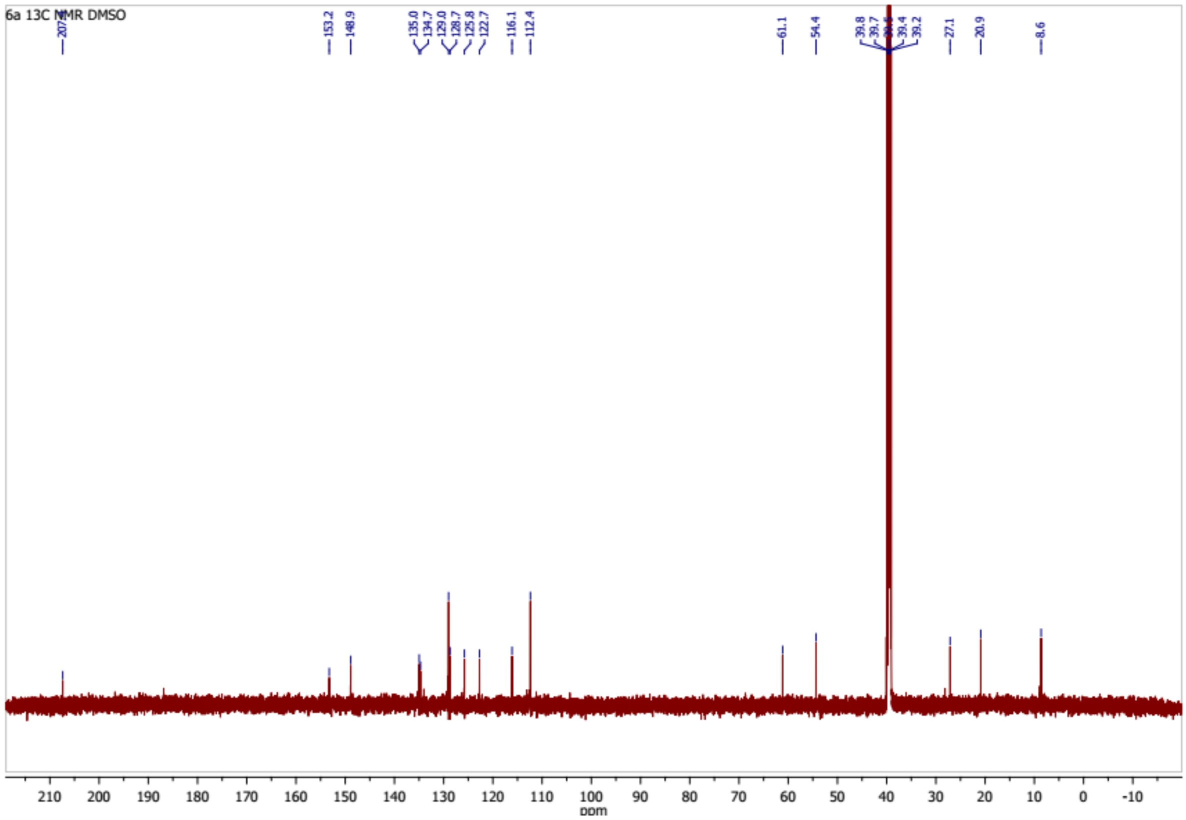

Figure S42. ^{13}C NMR spectrum of **6a** (DMSO-d_6).

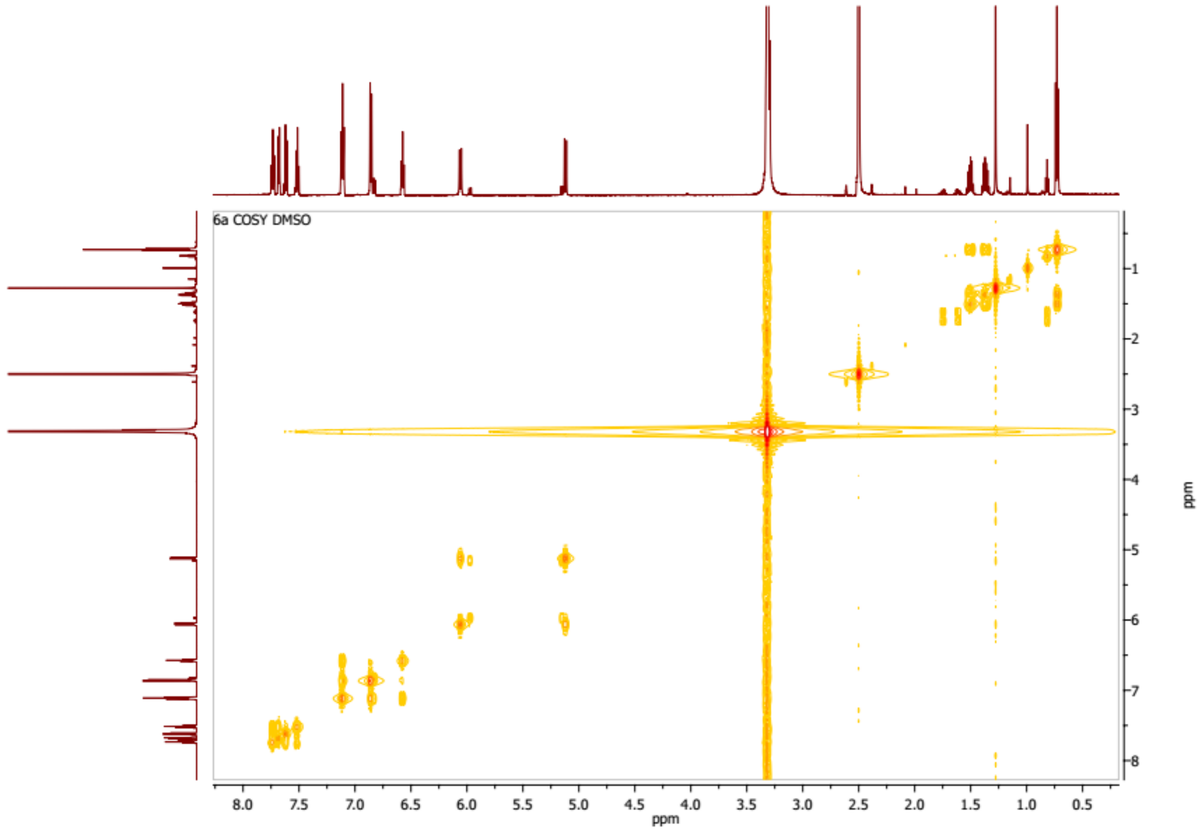

Figure S43. ^{1}H, ^{1}H COSY NMR spectrum of **6a** (DMSO-*d*$_6$).

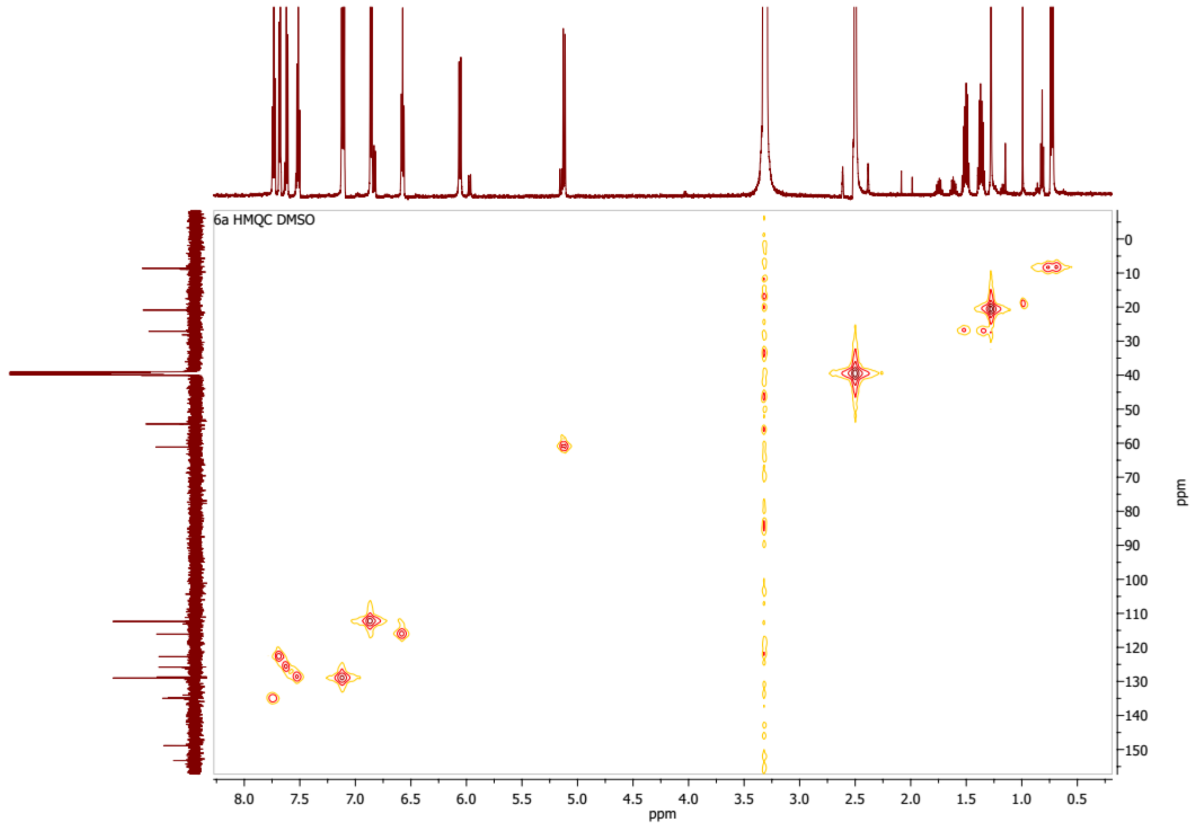

Figure S44. ^{1}H, ^{13}C HMQC NMR spectrum of **6a** (DMSO-*d*$_6$).

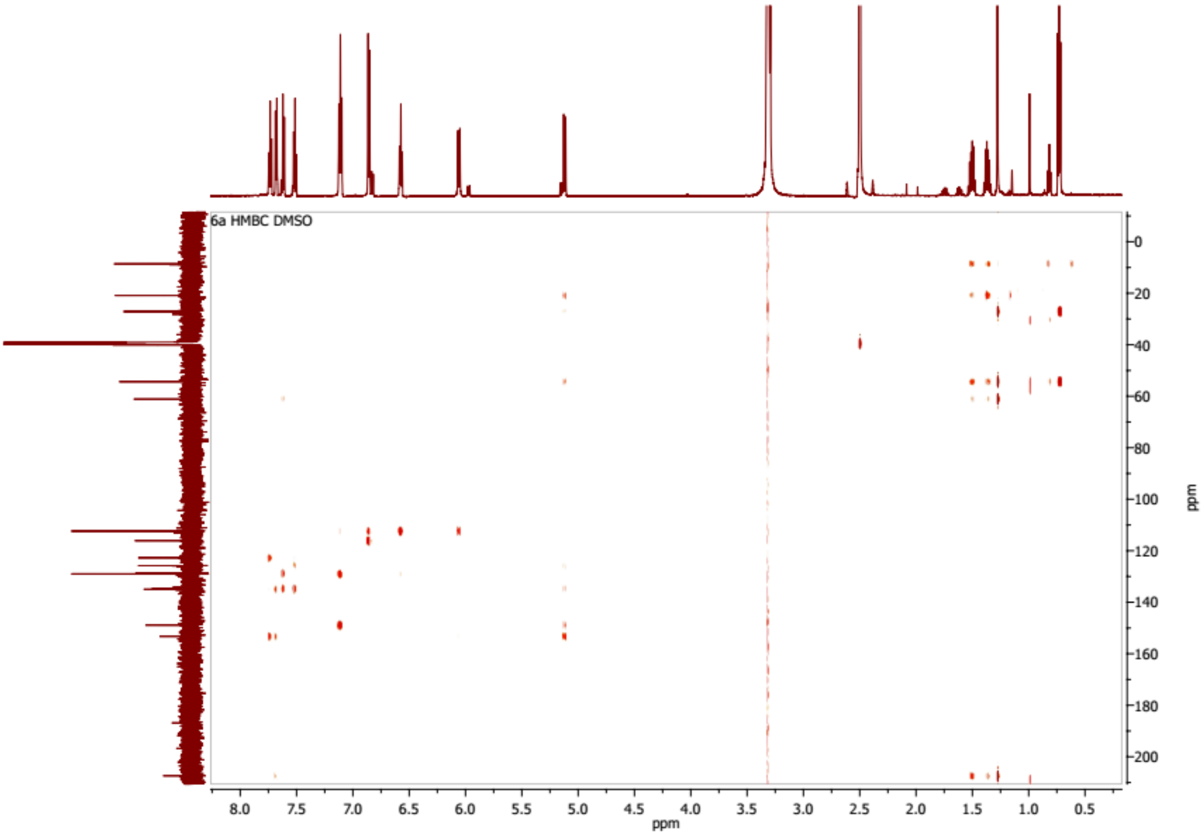

Figure S45. ^{1}H, ^{13}C HMBC NMR spectrum of **6a** (DMSO-d_6).

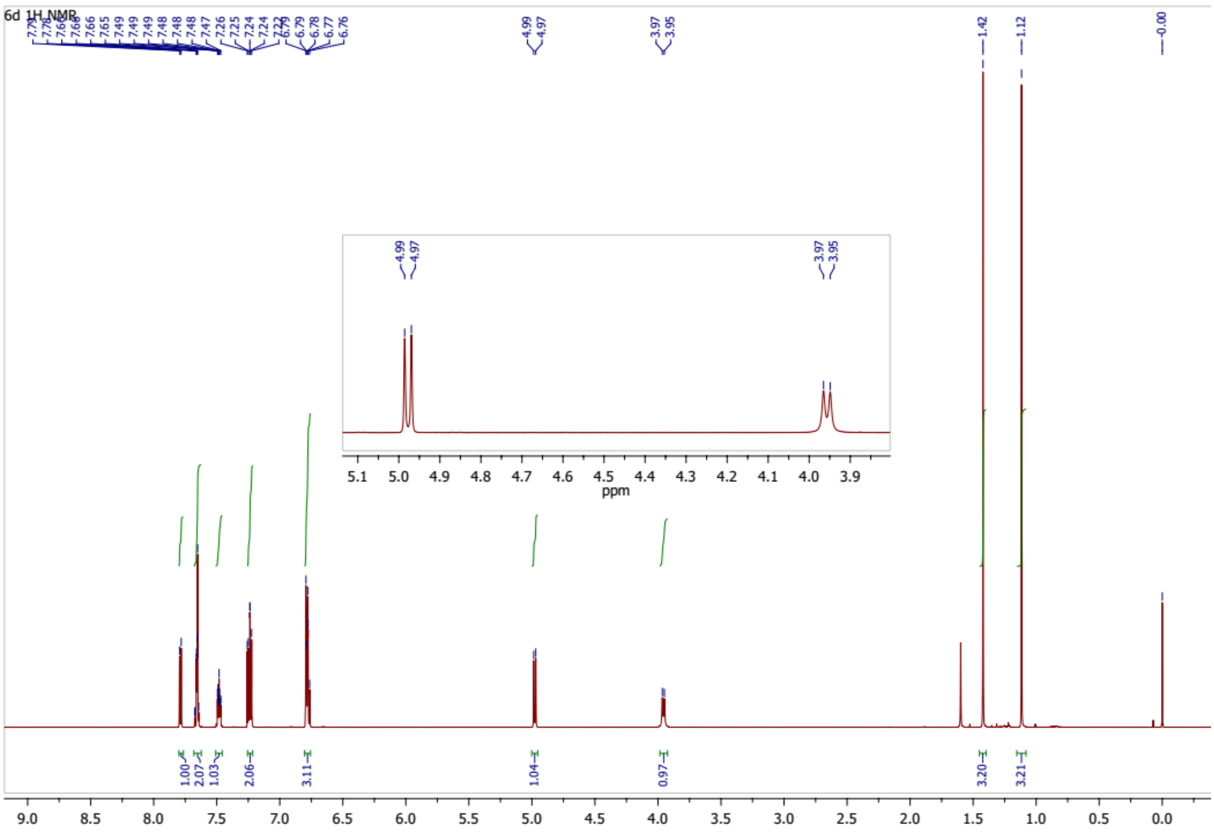

Figure S46. ^{1}H NMR spectrum of **6d**.

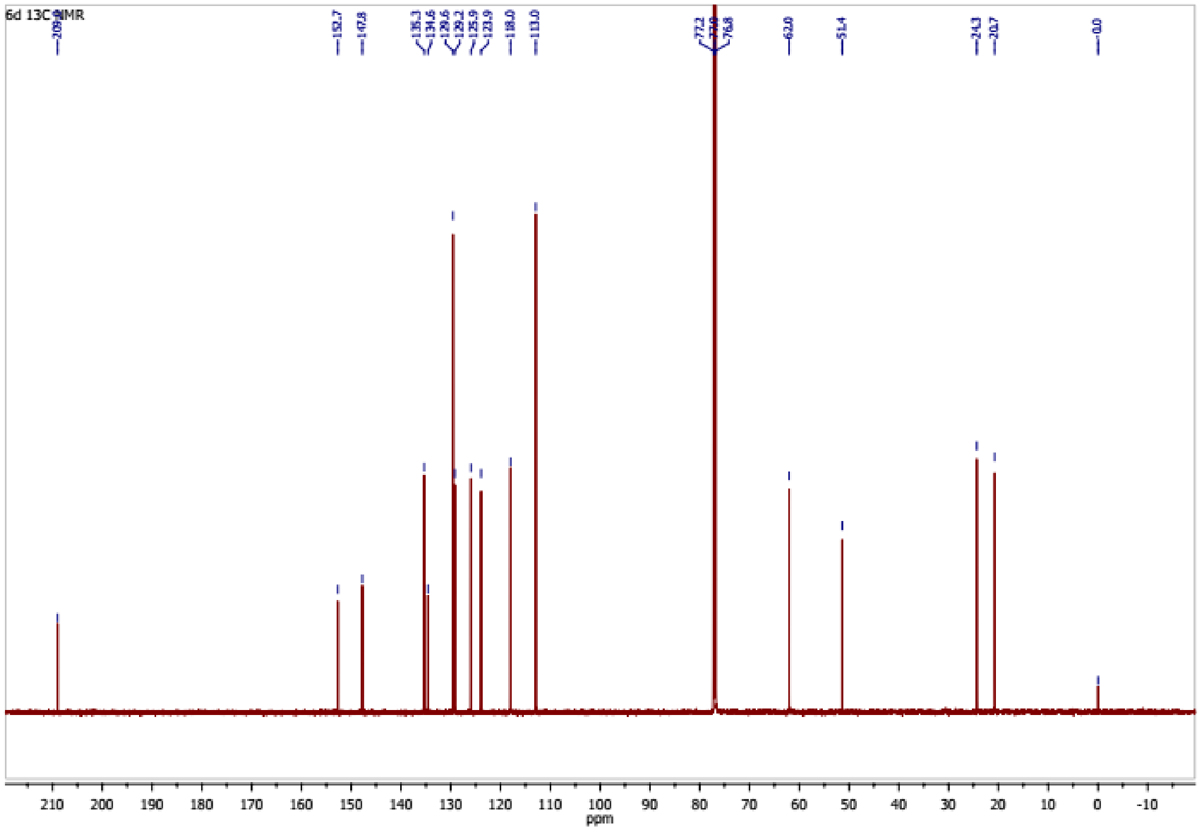

Figure S47. ^{13}C NMR spectrum of **6d**.

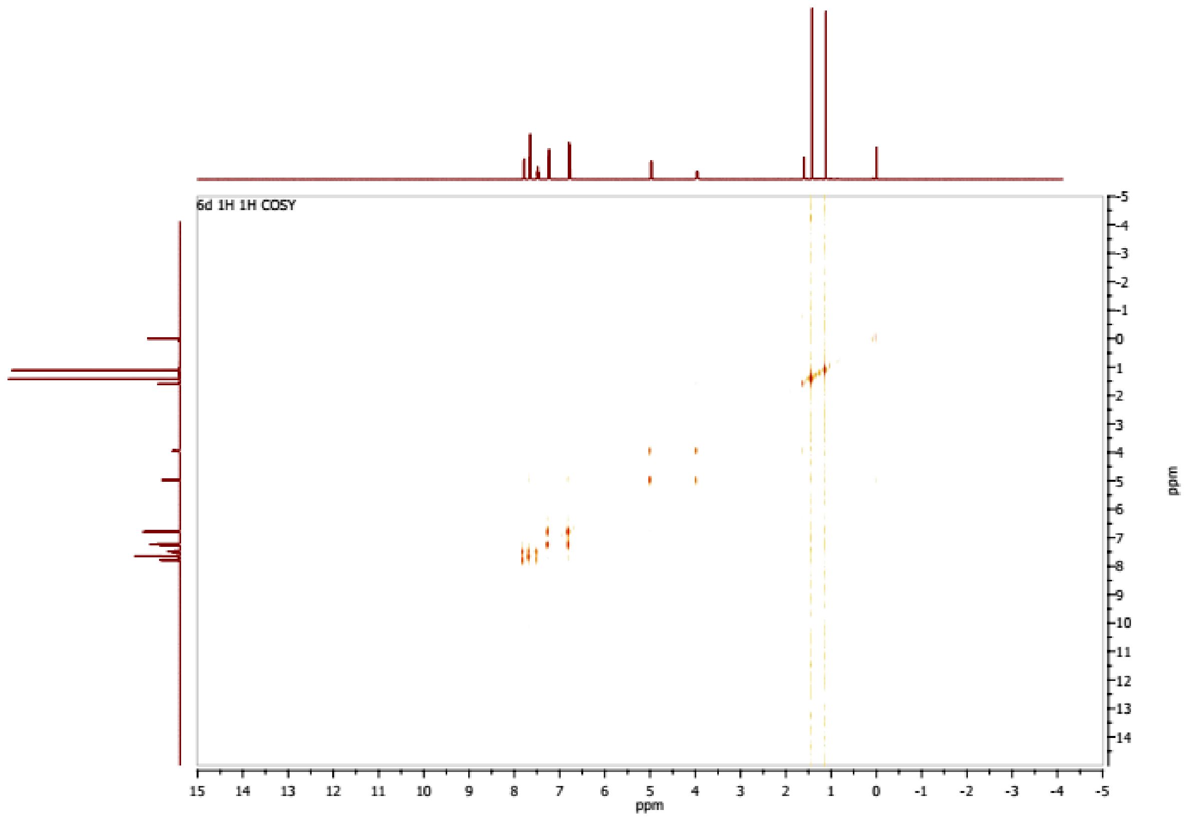

Figure S48. ^{1}H, ^{1}H COSY NMR spectrum of **6d**.

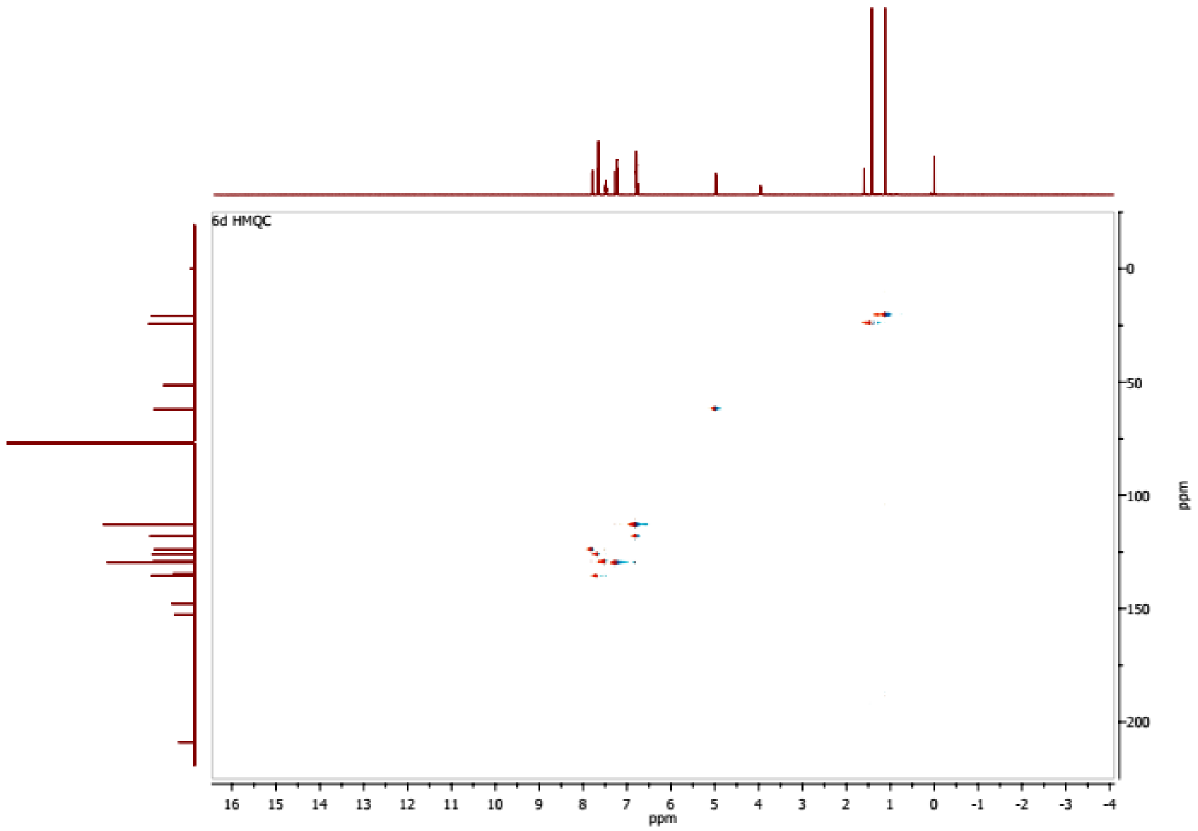

Figure S49. ^{1}H, ^{13}C HMQC NMR spectrum of **6d**.

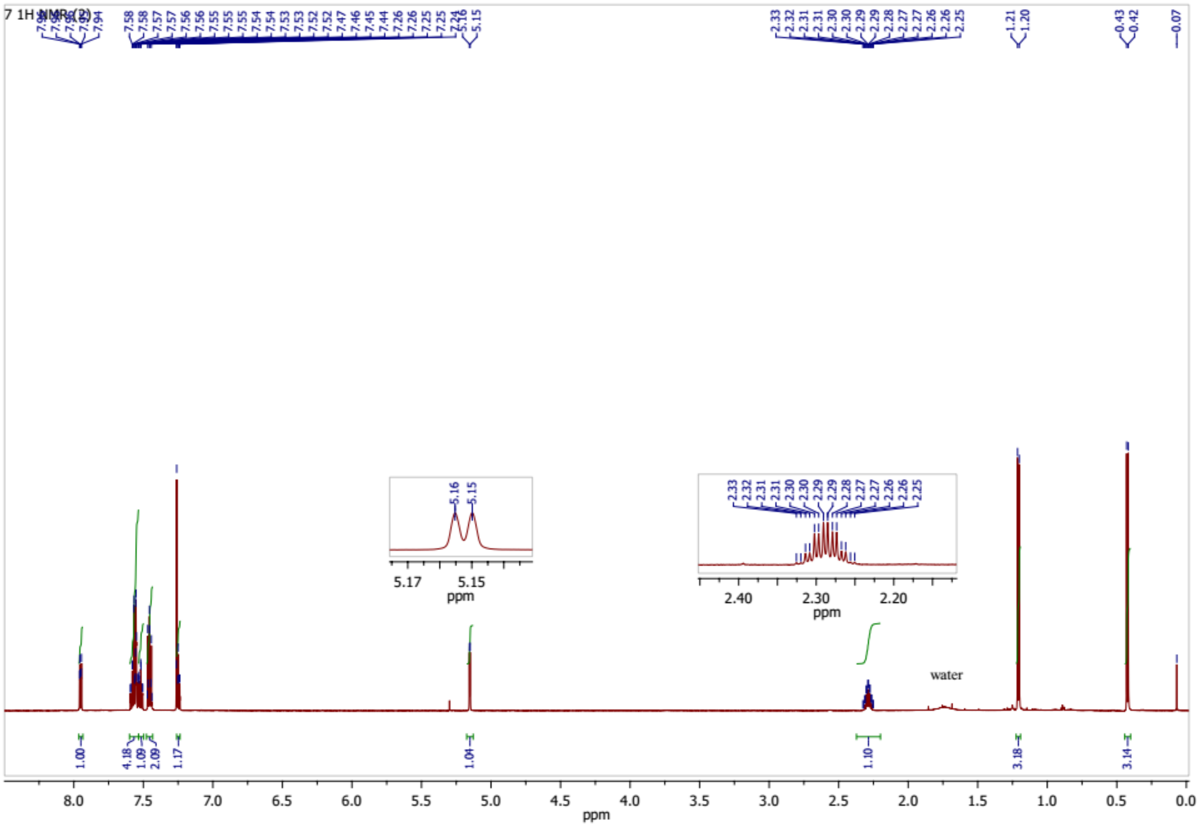

Figure S50. ^{1}H NMR spectrum of **7**.

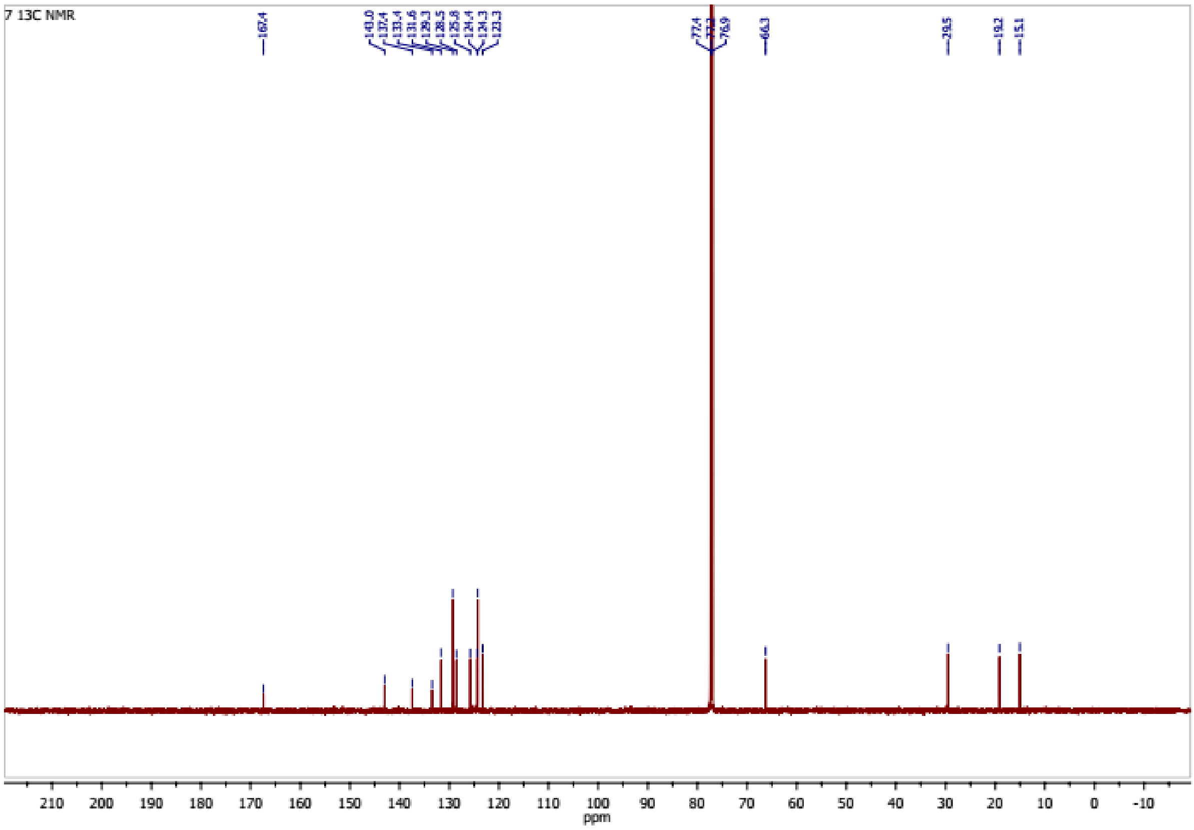

Figure S51. ^{13}C NMR spectrum of **7**.

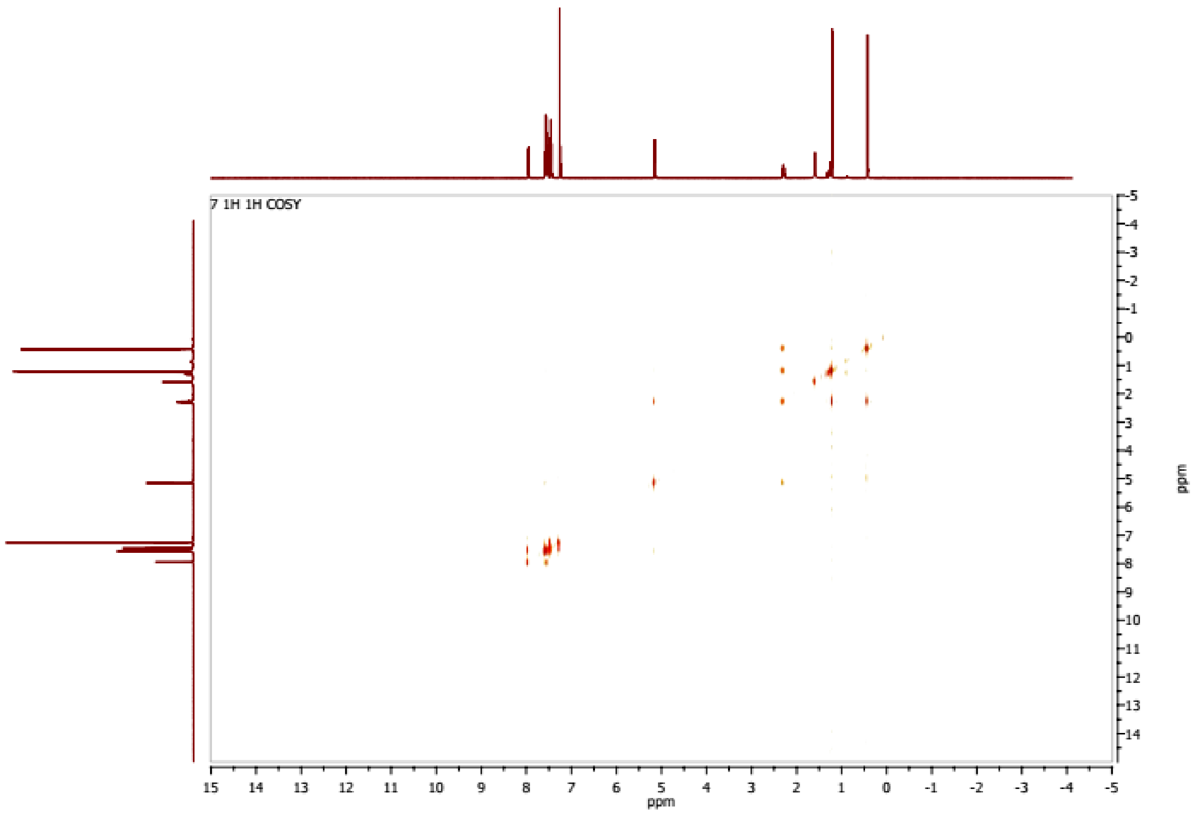

Figure S52. ^{1}H, ^{1}H COSY NMR spectrum of **7**.

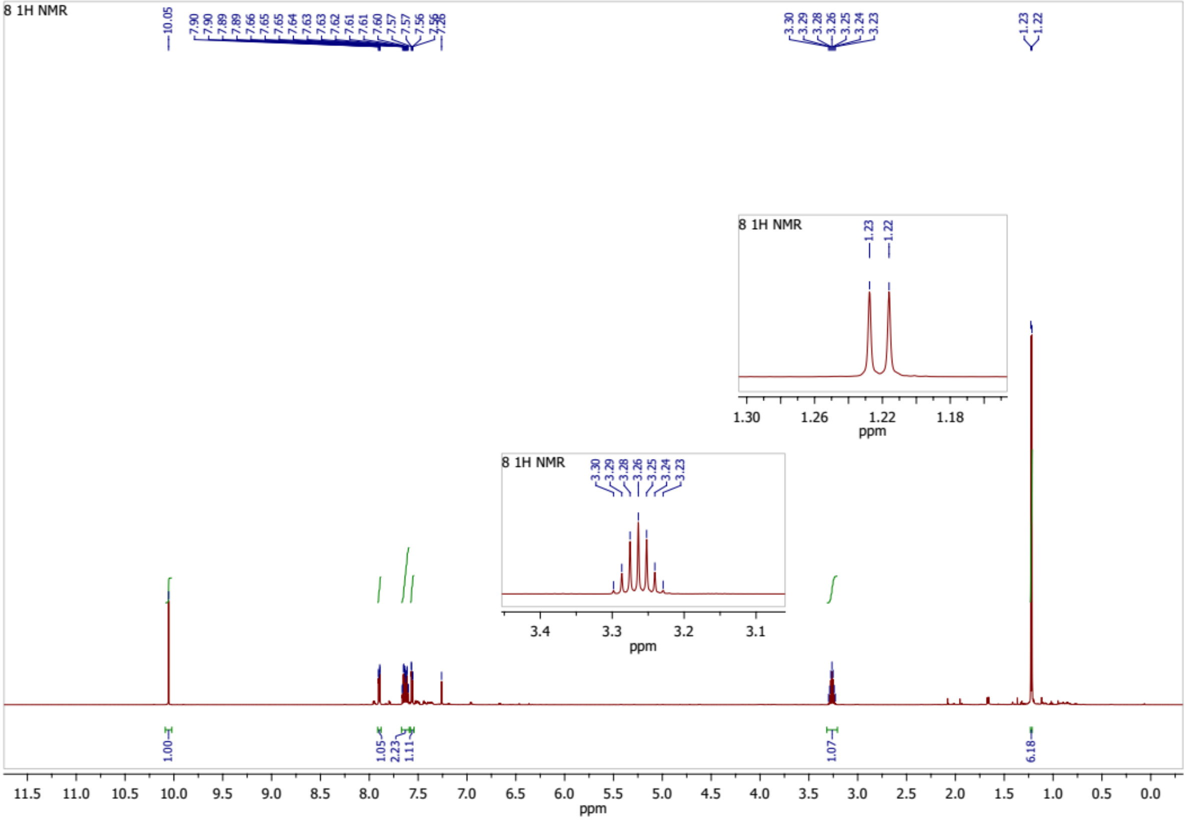

Figure S53. ^{1}H NMR spectrum of **8**.

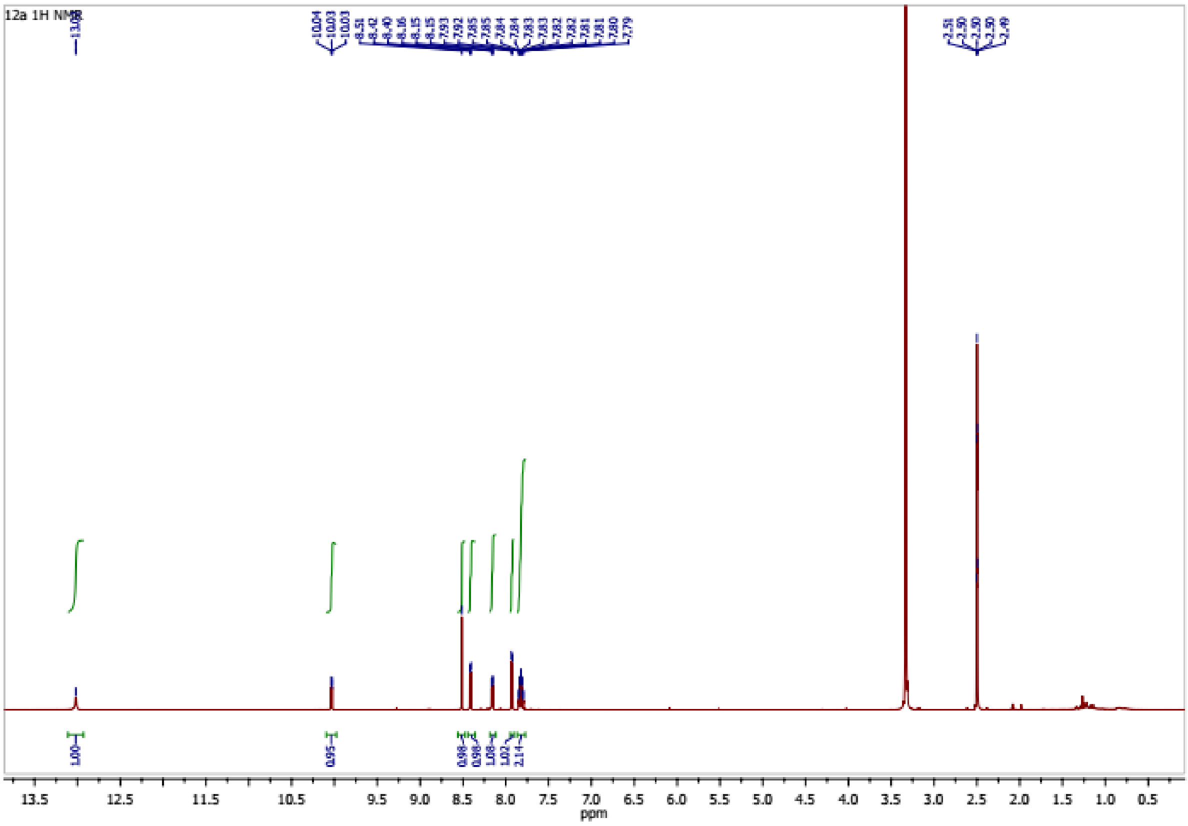

Figure S54. ^{1}H NMR spectrum of **12a**.

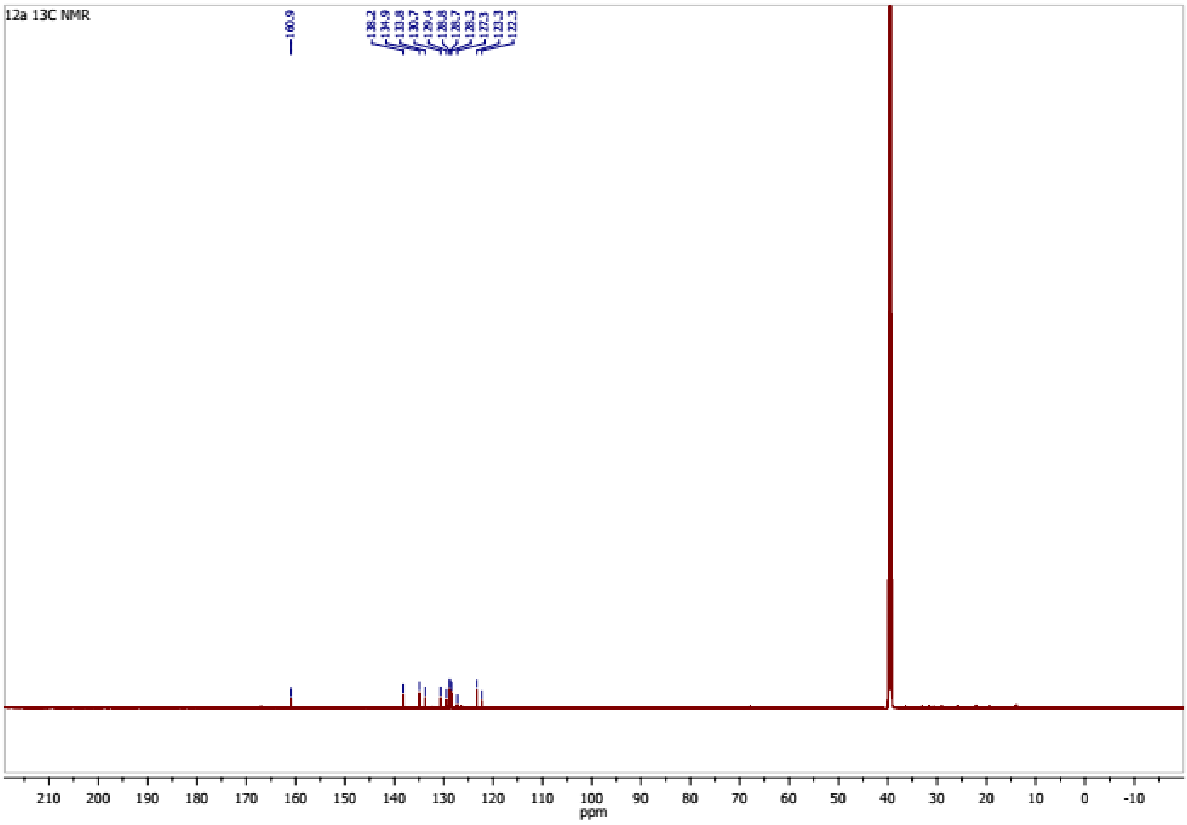

Figure S55. ^{13}C NMR spectrum of **12a**.

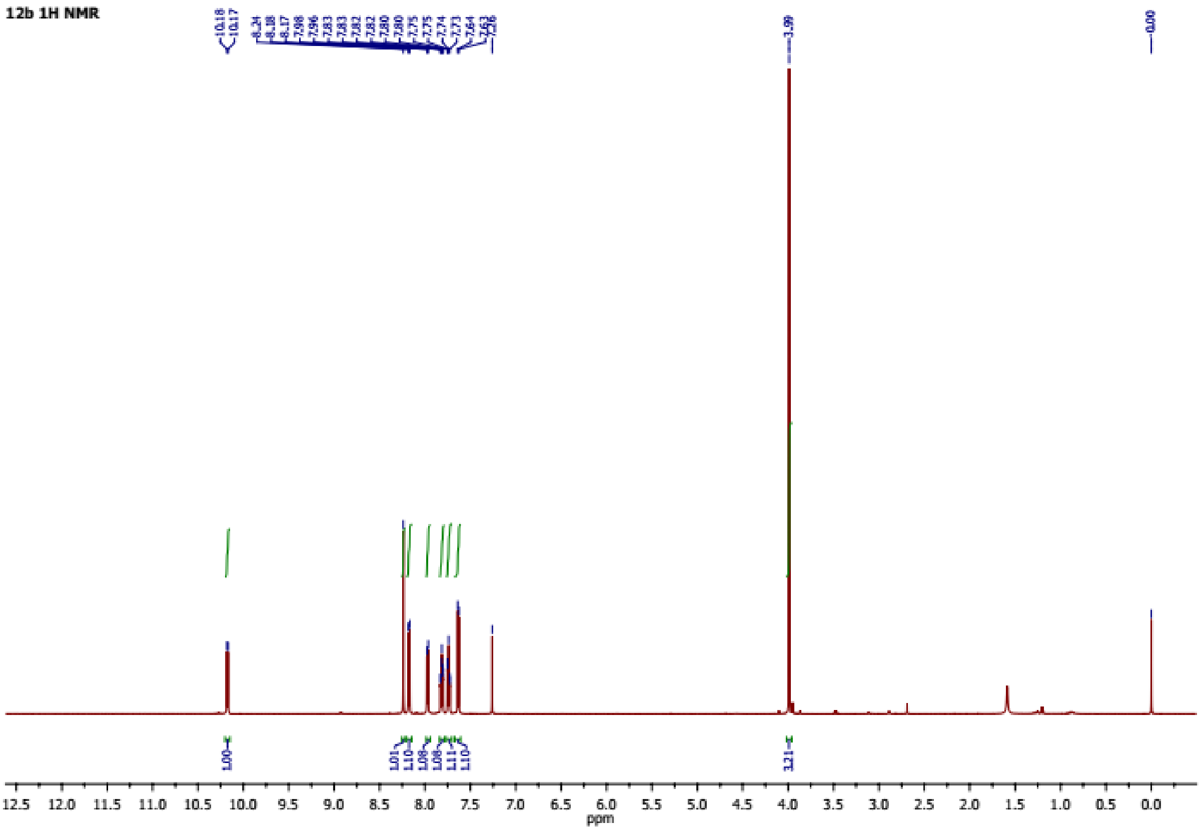

Figure S56. ^{1}H NMR spectrum of **12b**.

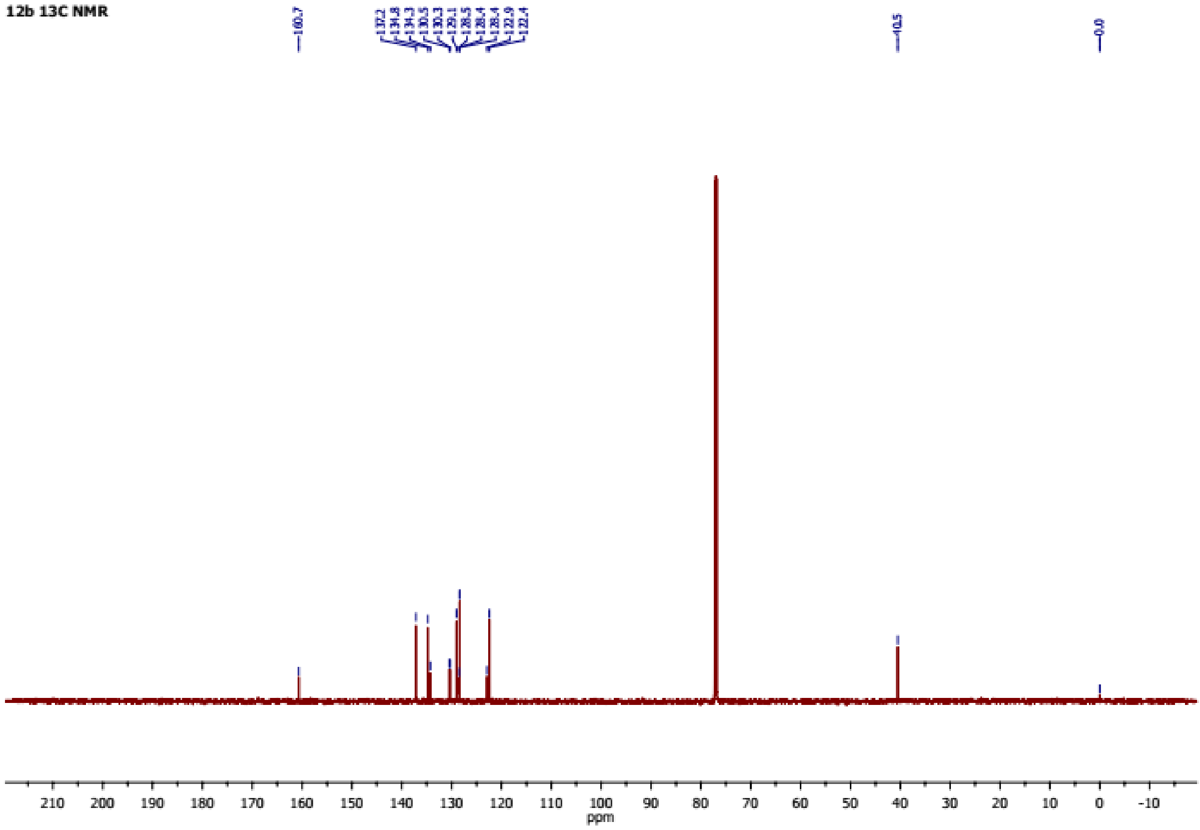

Figure S57. ^{13}C NMR spectrum of **12b**.

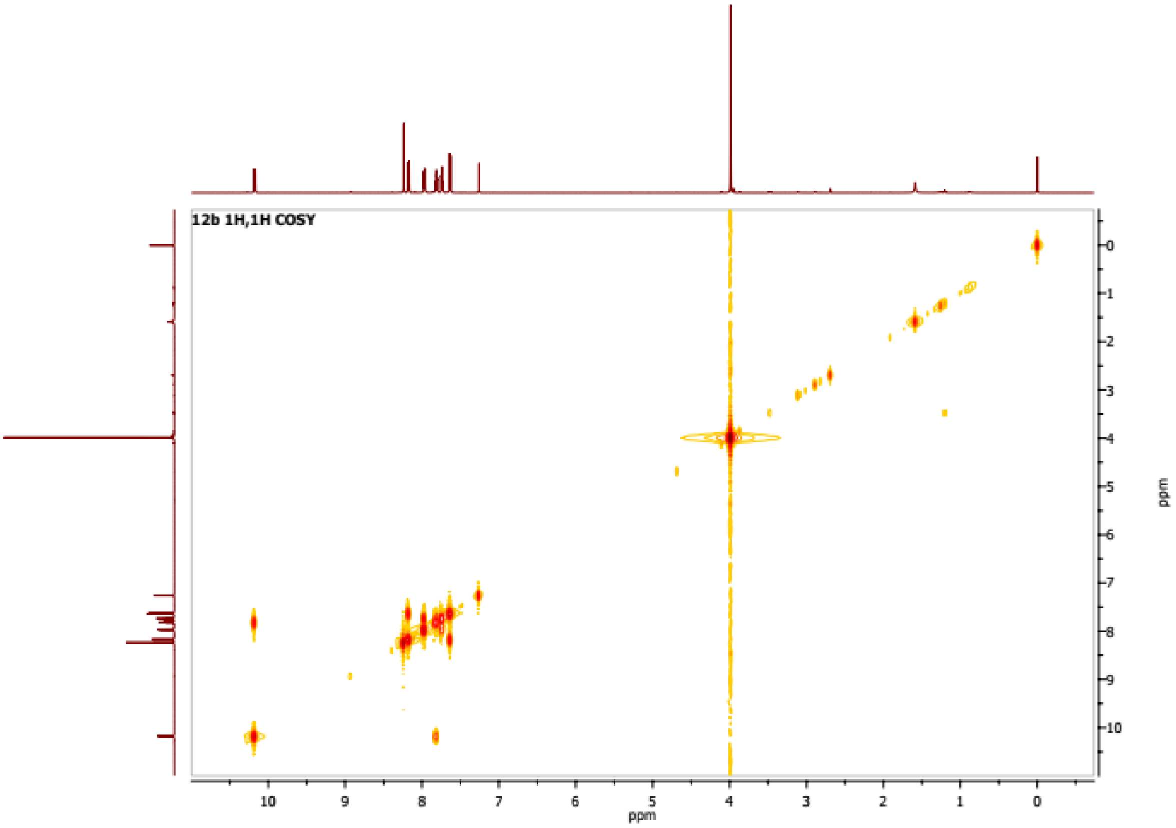

Figure S58. ^{1}H, ^{1}H COSY NMR spectrum of **12b**.

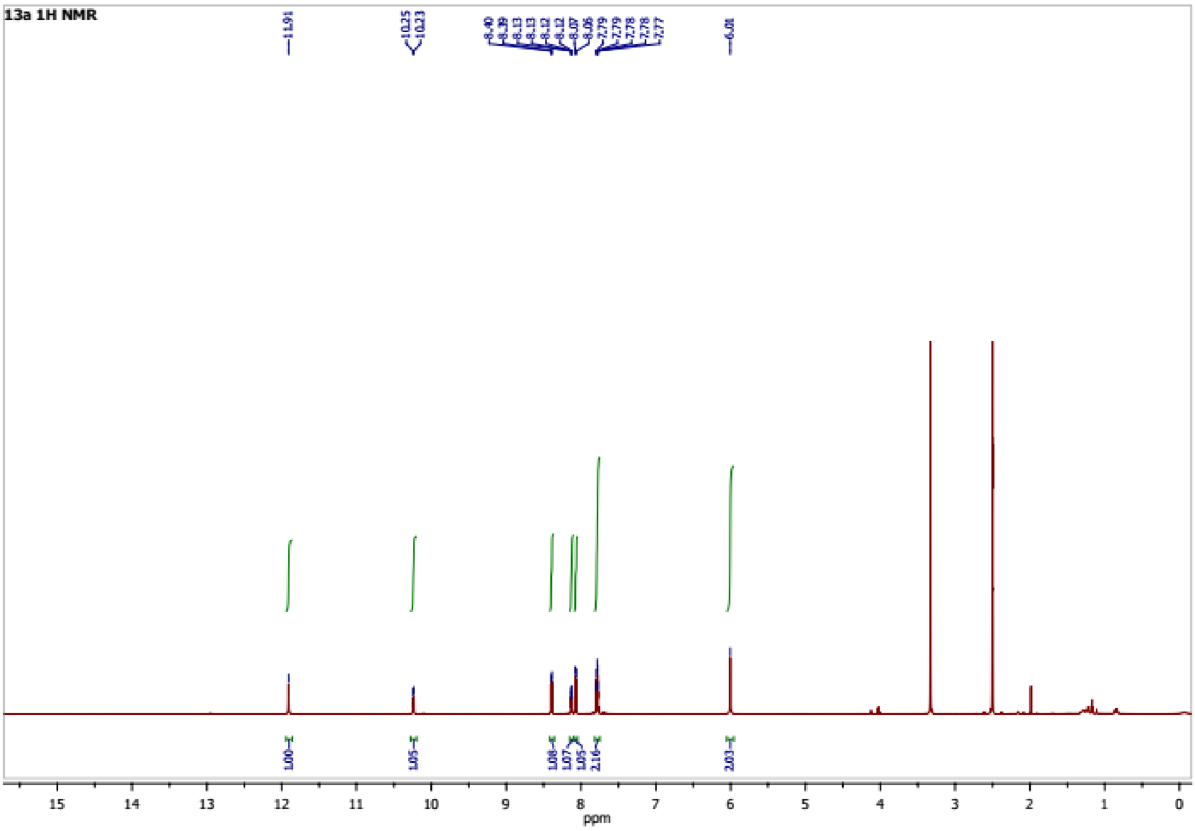

Figure S58. ^{1}H NMR spectrum of **13a**.

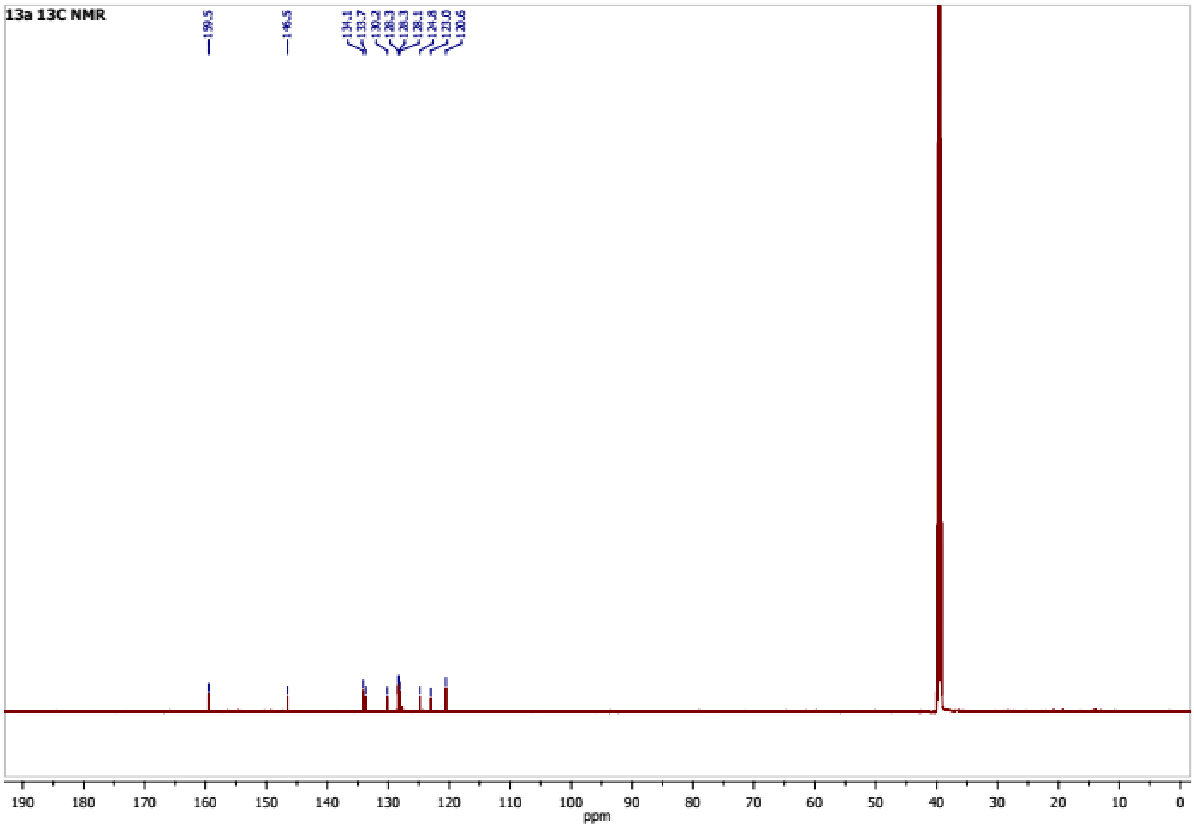

Figure S59. ^{13}C NMR spectrum of **13a**.

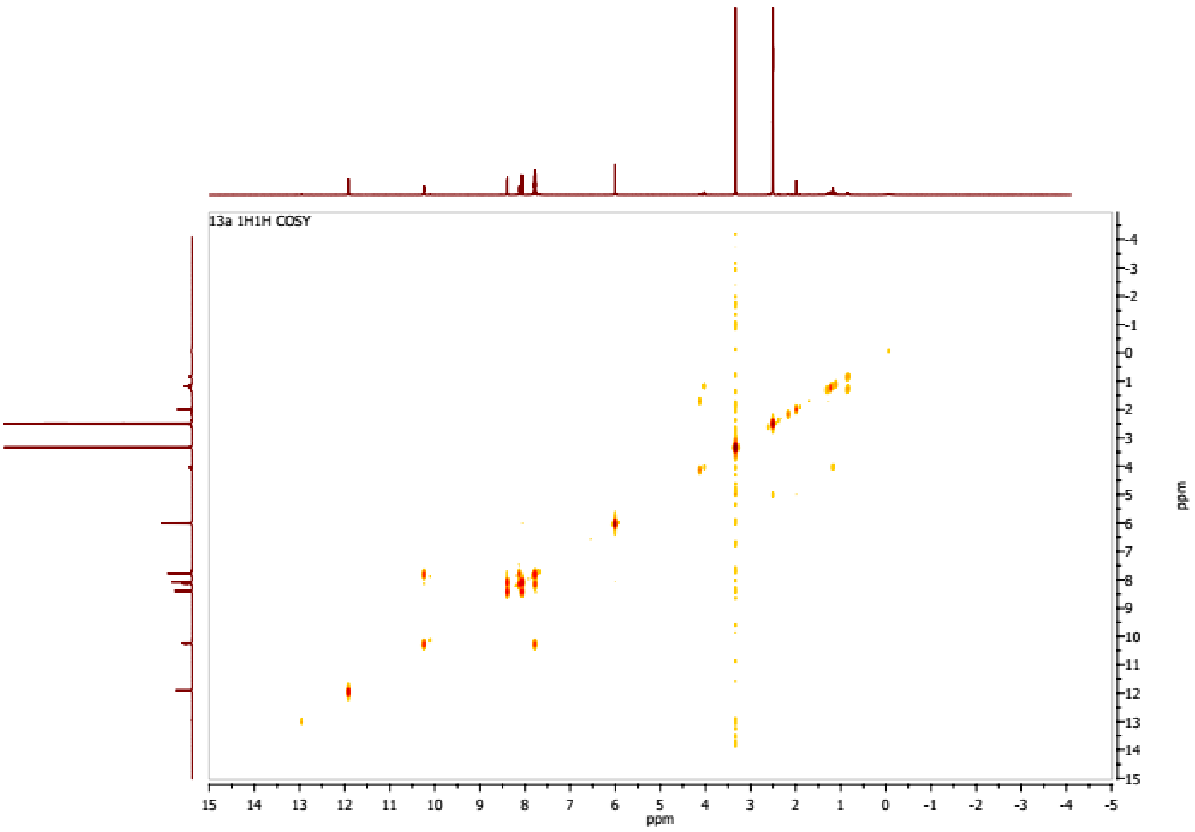

Figure S60. ^{1}H, ^{1}H COSY NMR spectrum of **13a**.

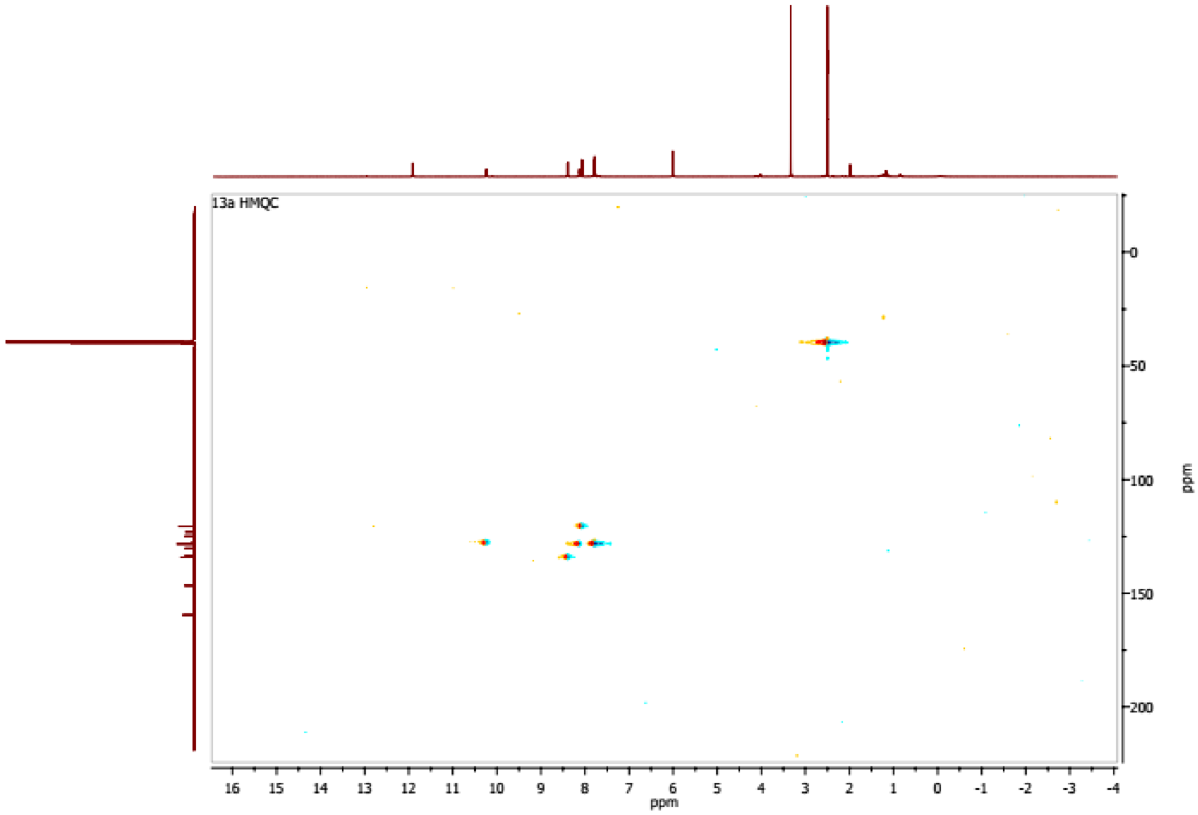

Figure S61. ^{1}H, ^{13}C HMQC NMR spectrum of **13a**.

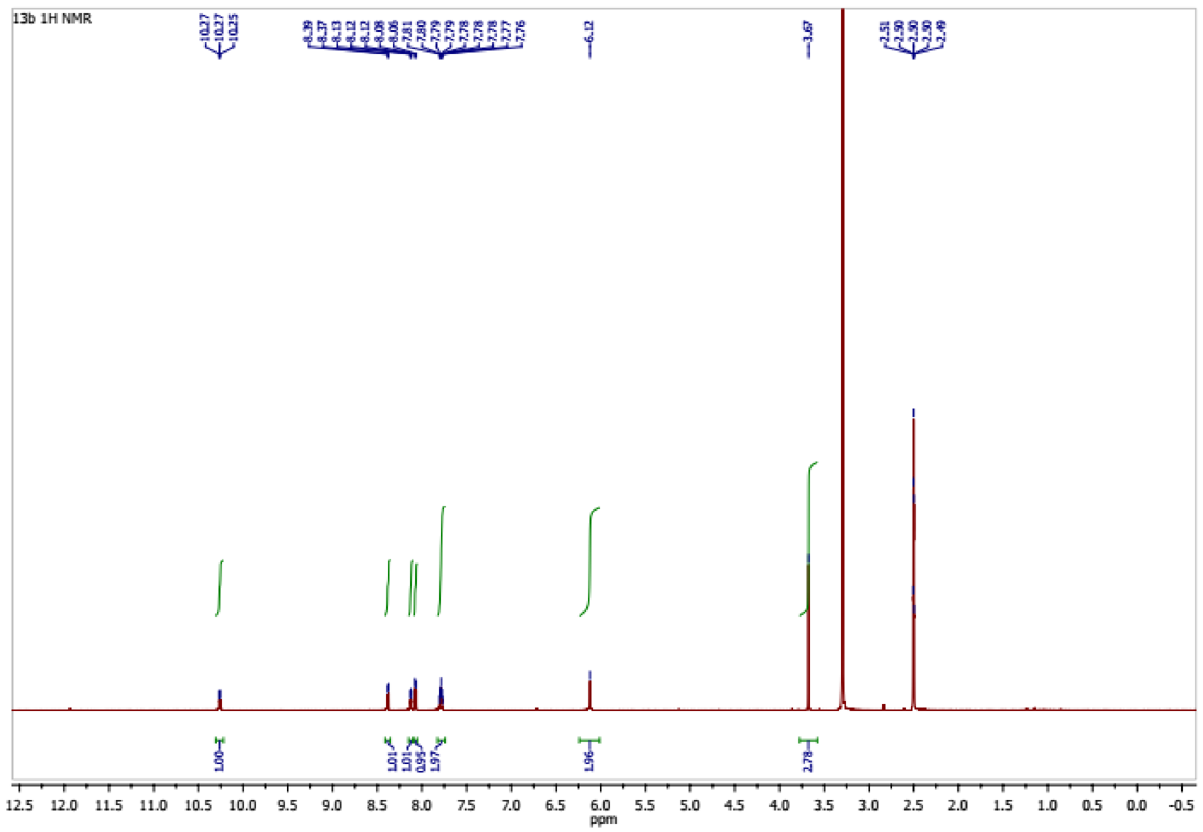

Figure S62. ^{1}H NMR spectrum of **13b**.

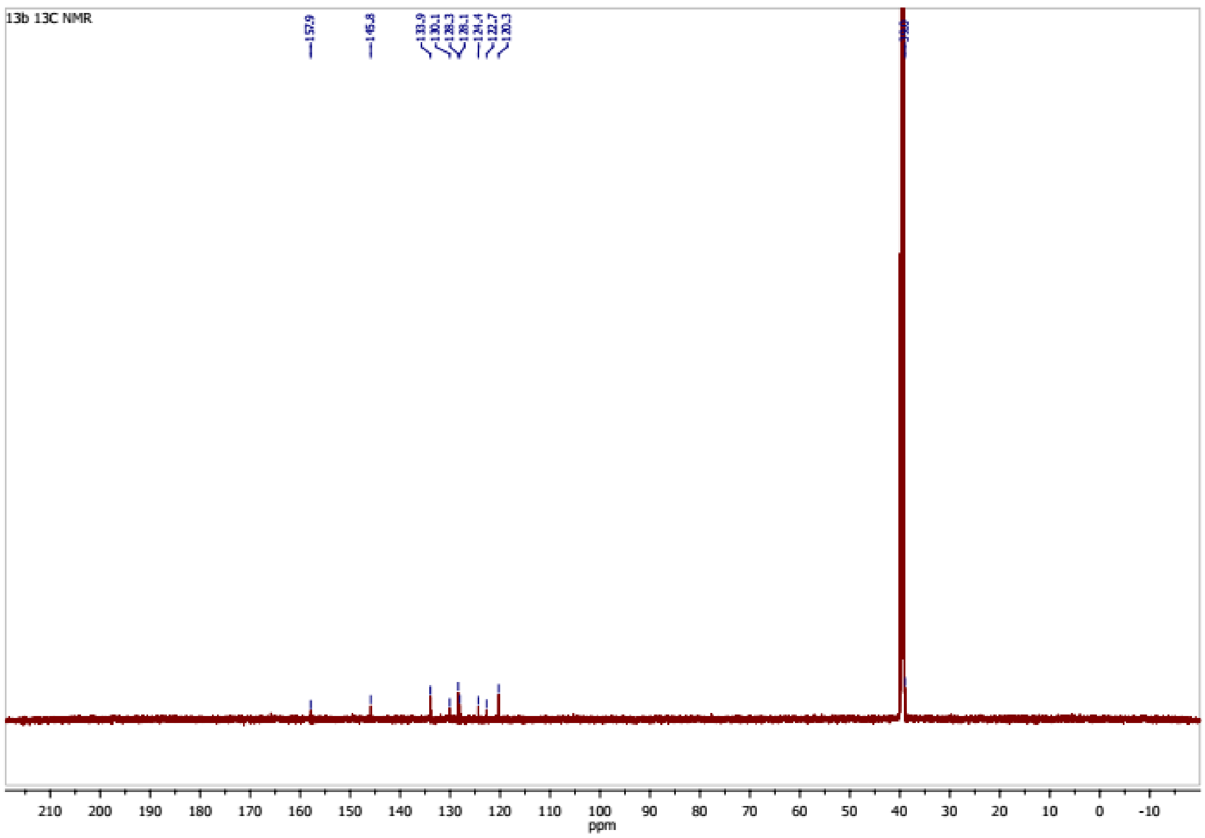

Figure S63. ^{13}C NMR spectrum of **13b**.

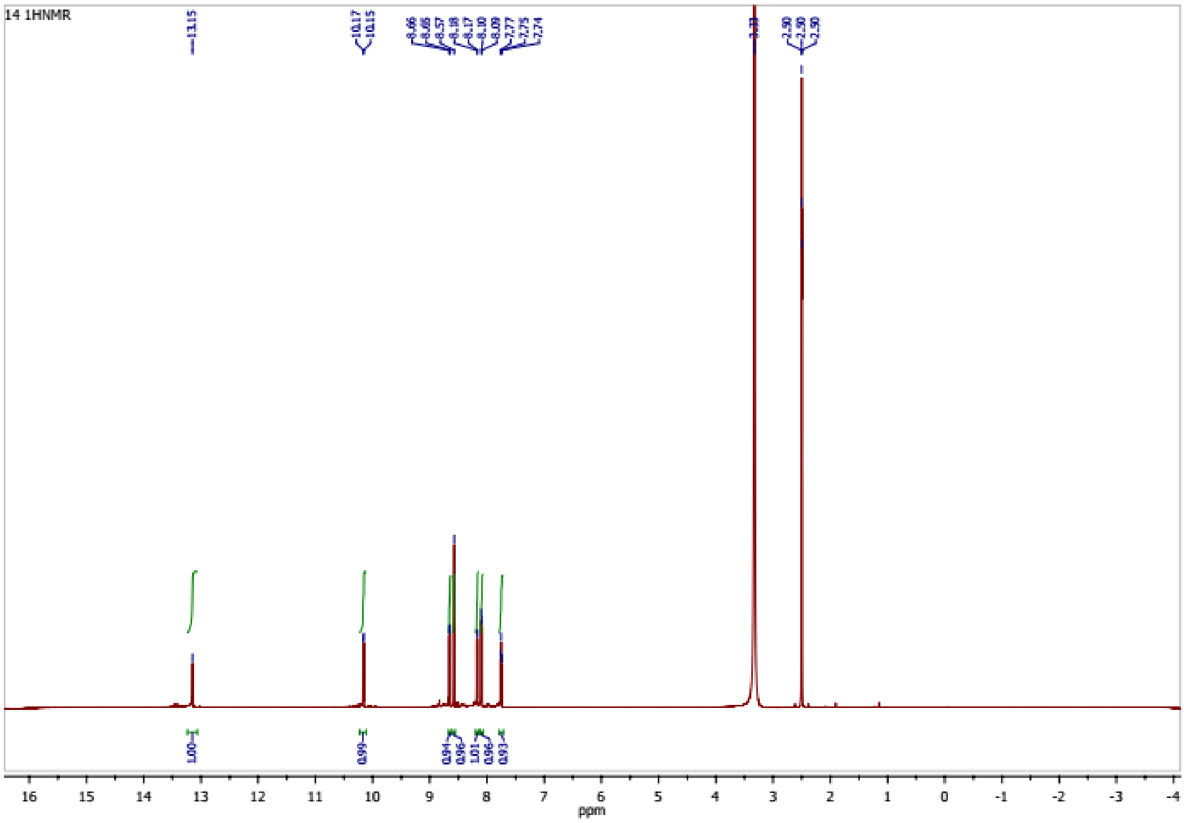

Figure S64. ^{1}H NMR spectrum of **14**.

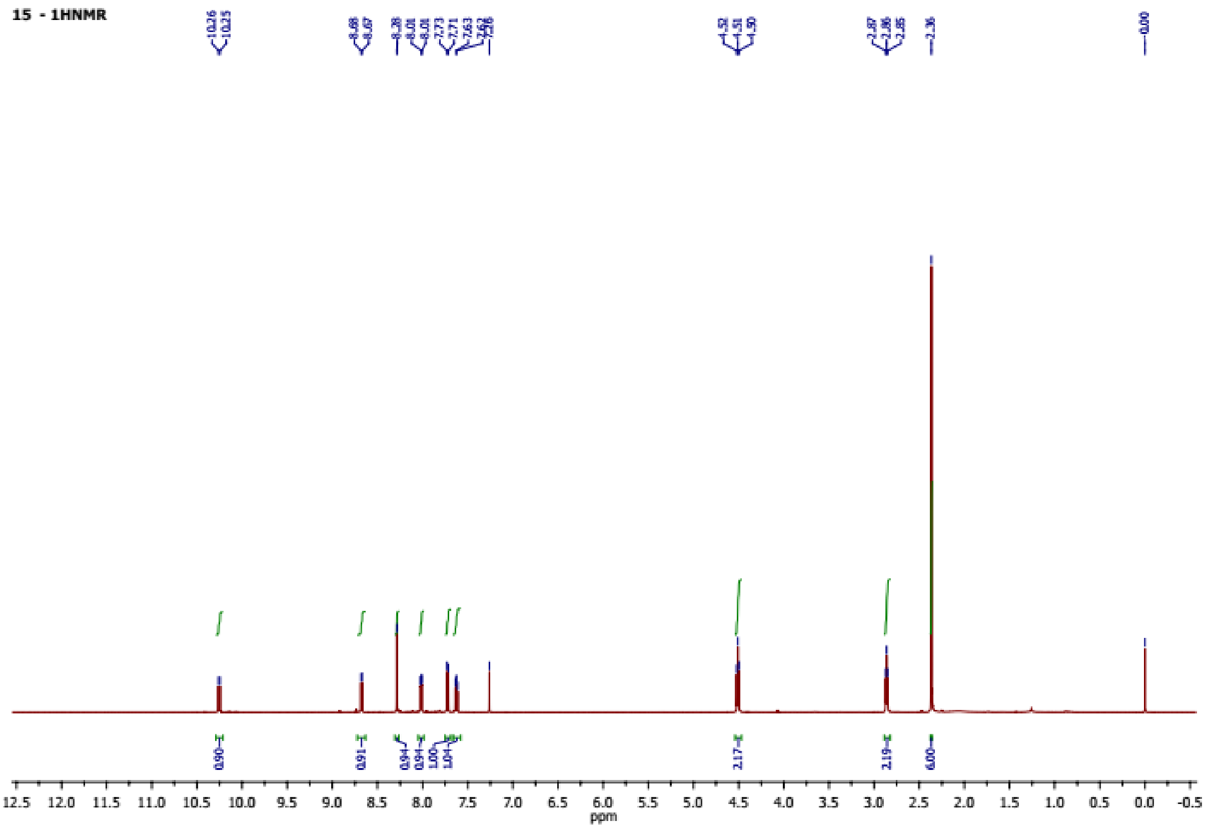

Figure S65. ^{1}H NMR spectrum of **15**.

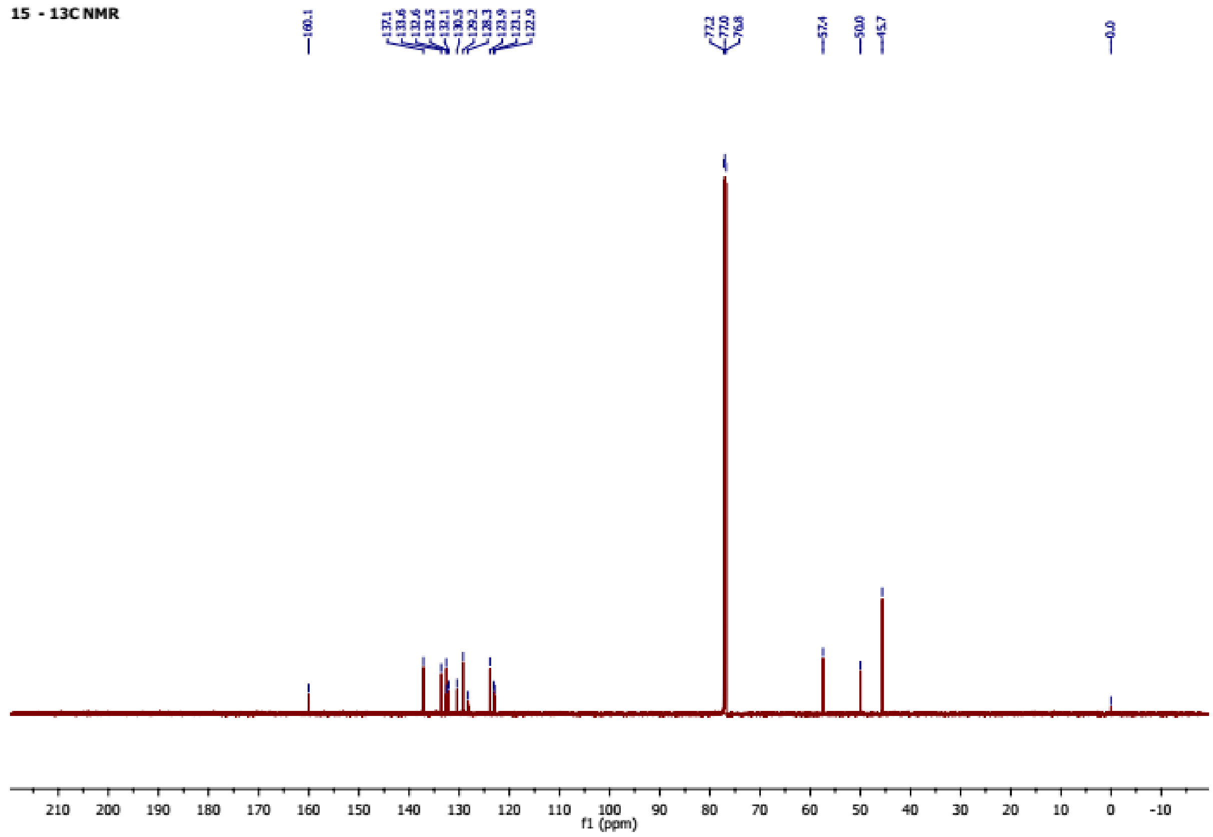

Figure S66. ^{13}C NMR spectrum of **15**.

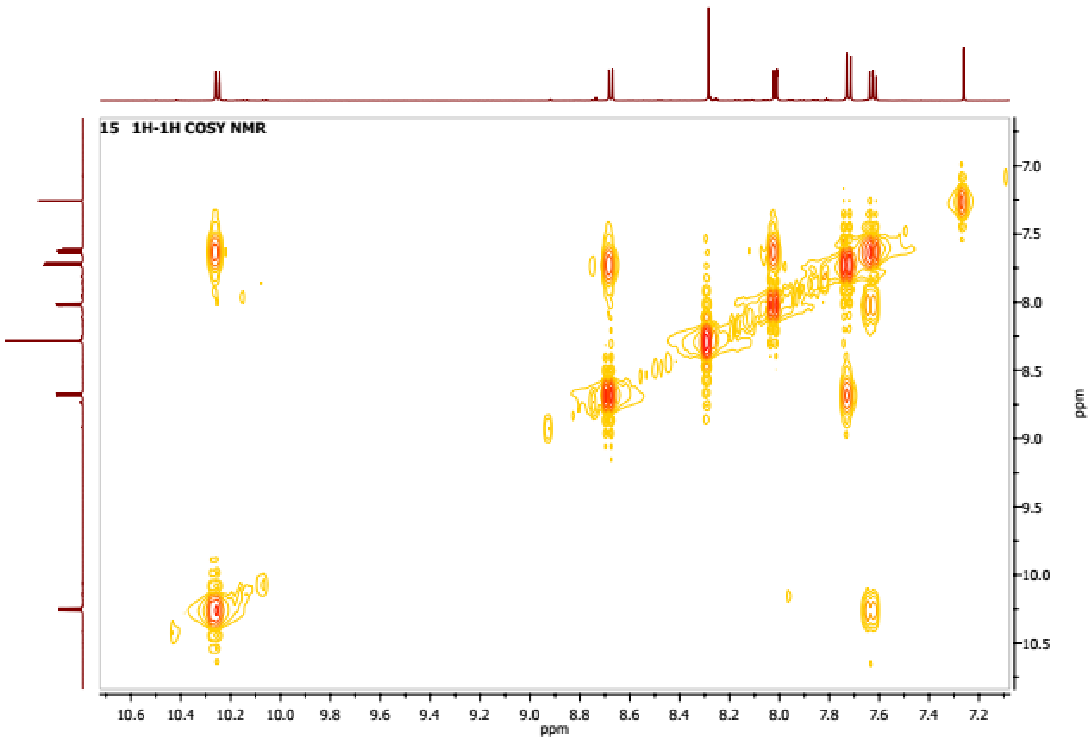

Figure S67. ^{1}H, ^{1}H COSY NMR spectrum of **15**.

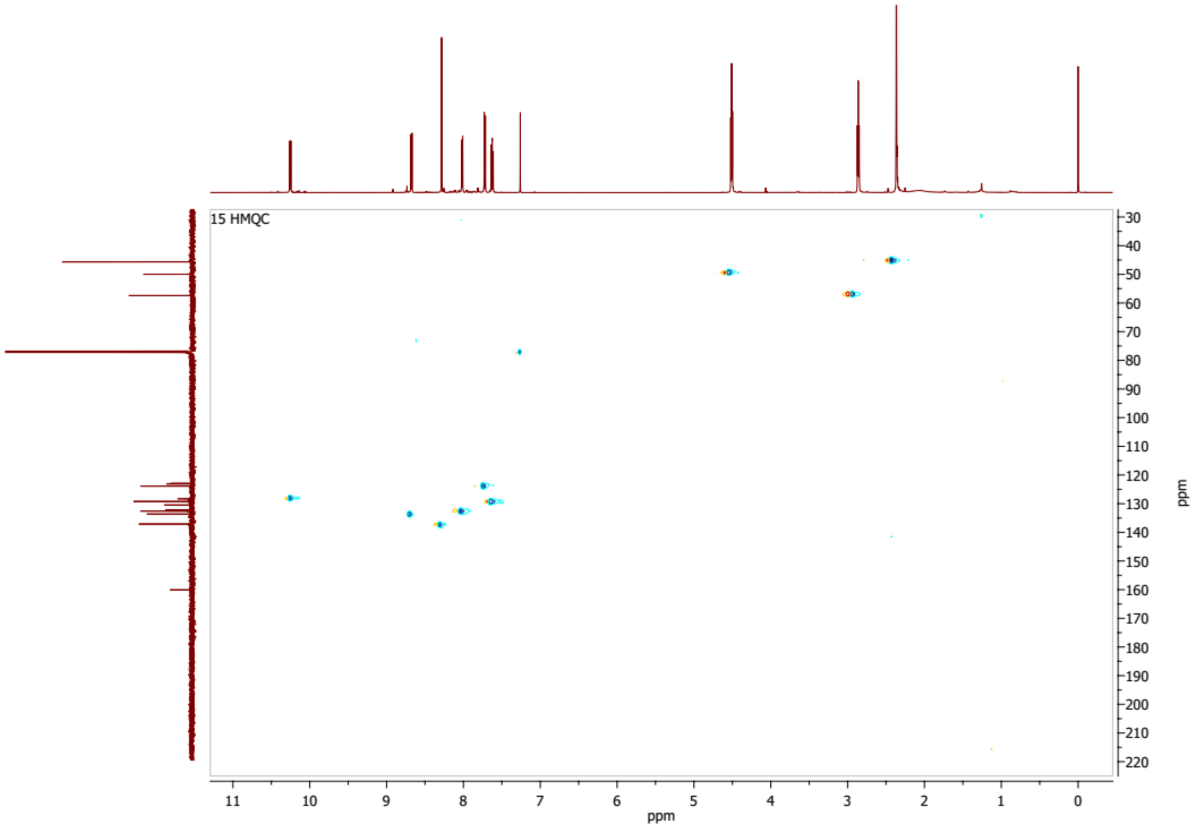

Figure S68. ^{1}H, ^{13}C HMQC NMR spectrum of **15**.

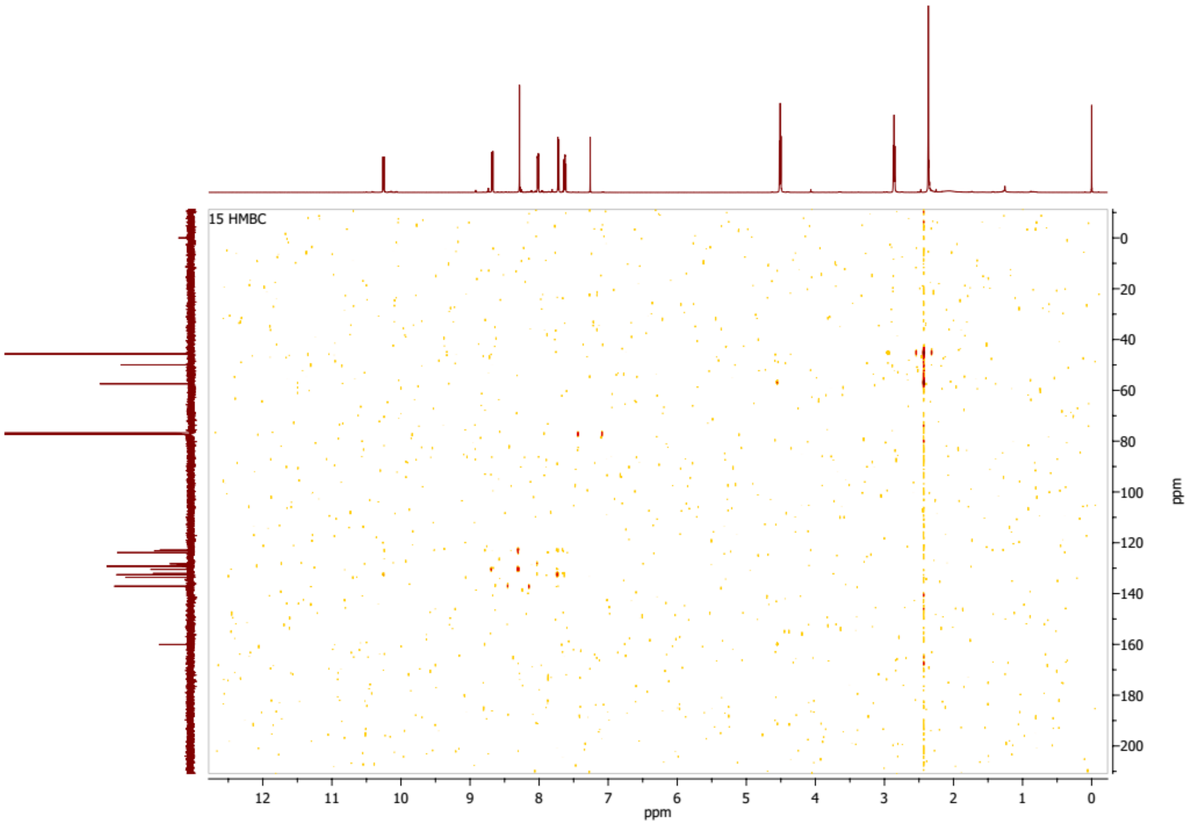

Figure S69. ^{1}H, ^{13}C HMBC NMR spectrum of **15**.

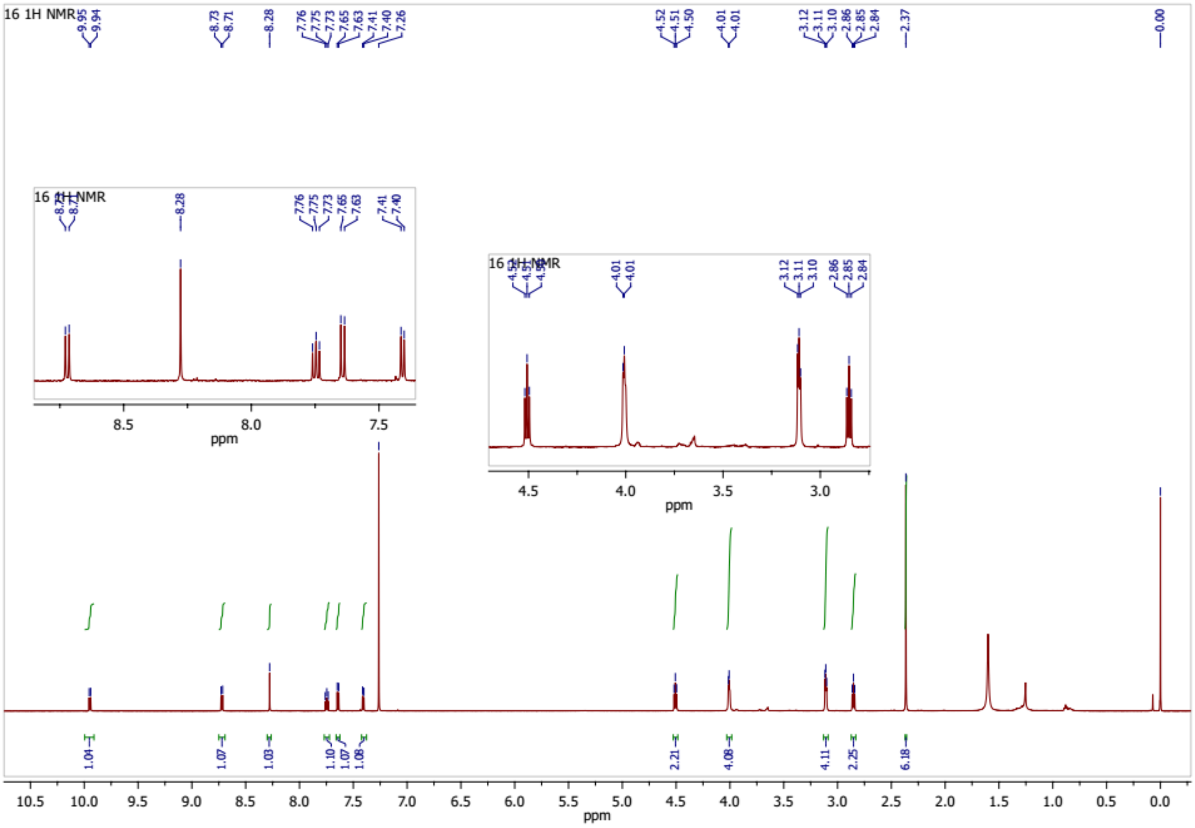

Figure S70. ^{1}H NMR spectrum of **16**.

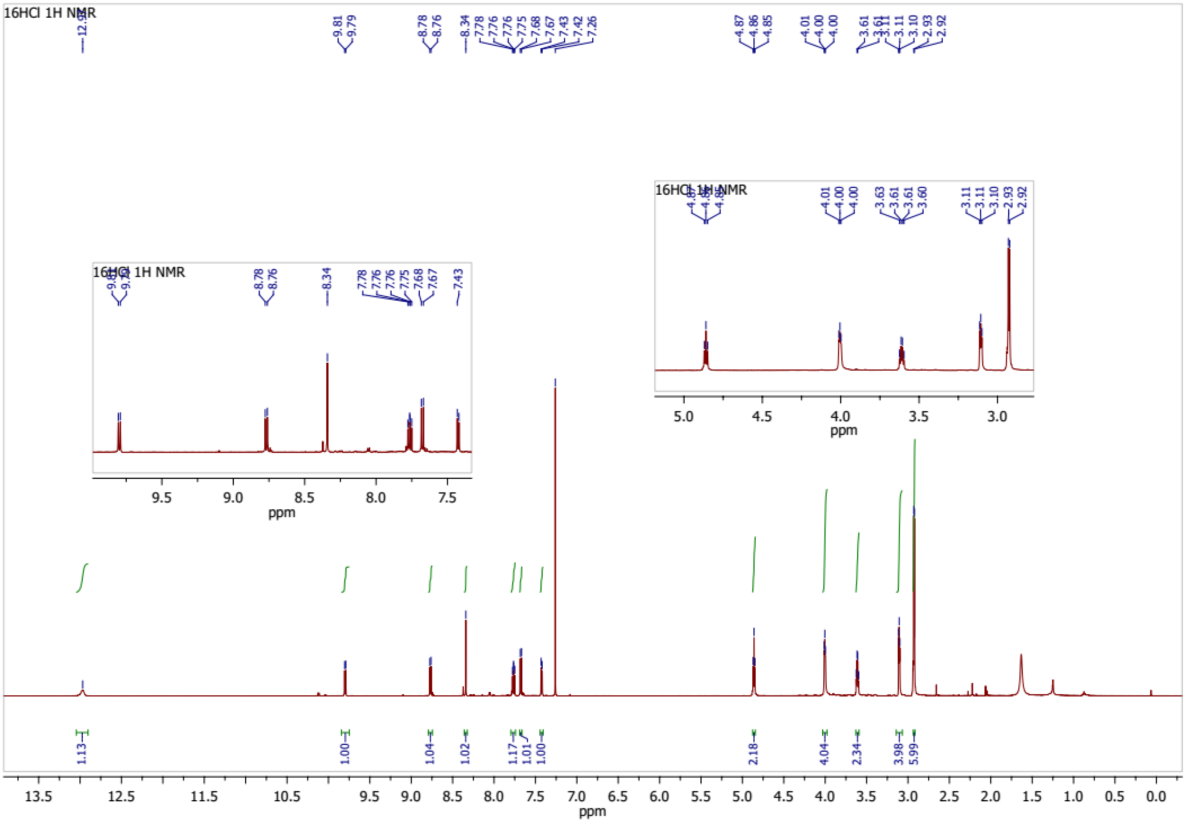

Figure S71. ^{1}H NMR spectrum of **16•HCl**.

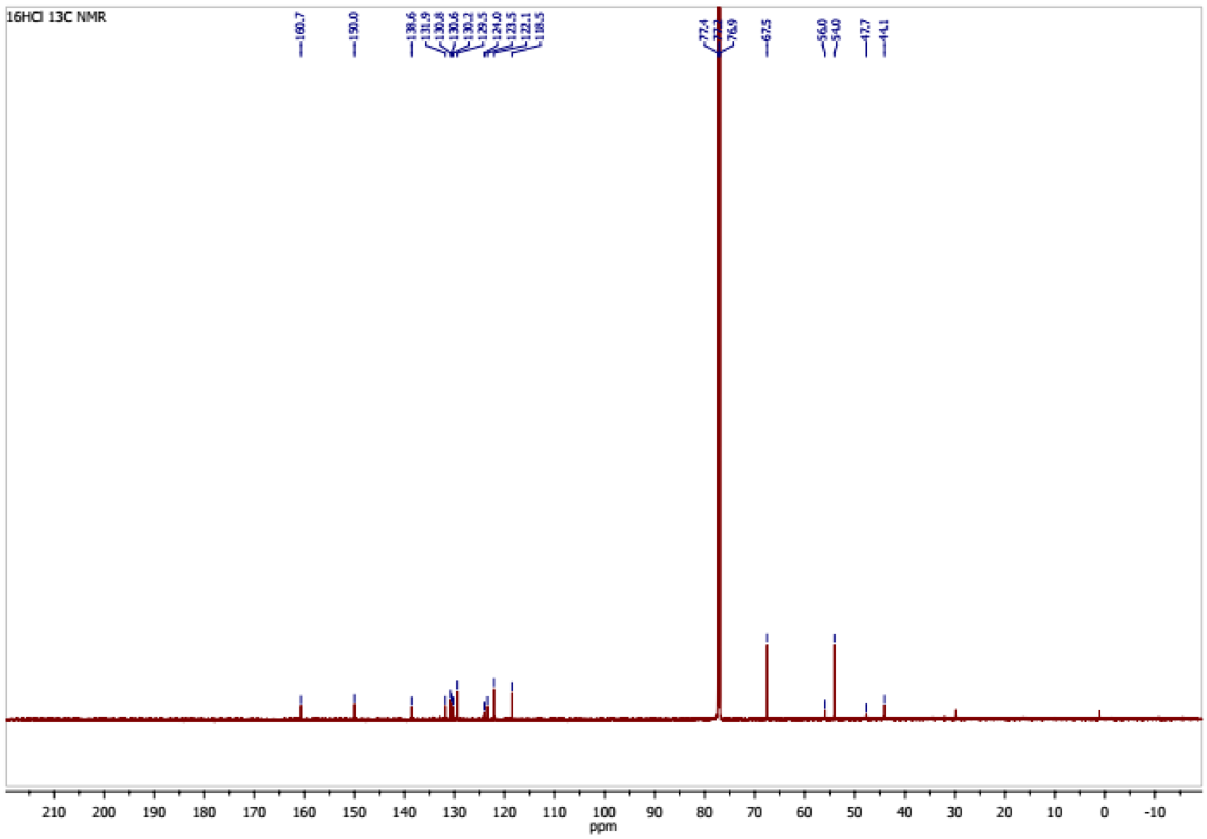

Figure S72. ^{13}C NMR spectrum of **16•HCl**.

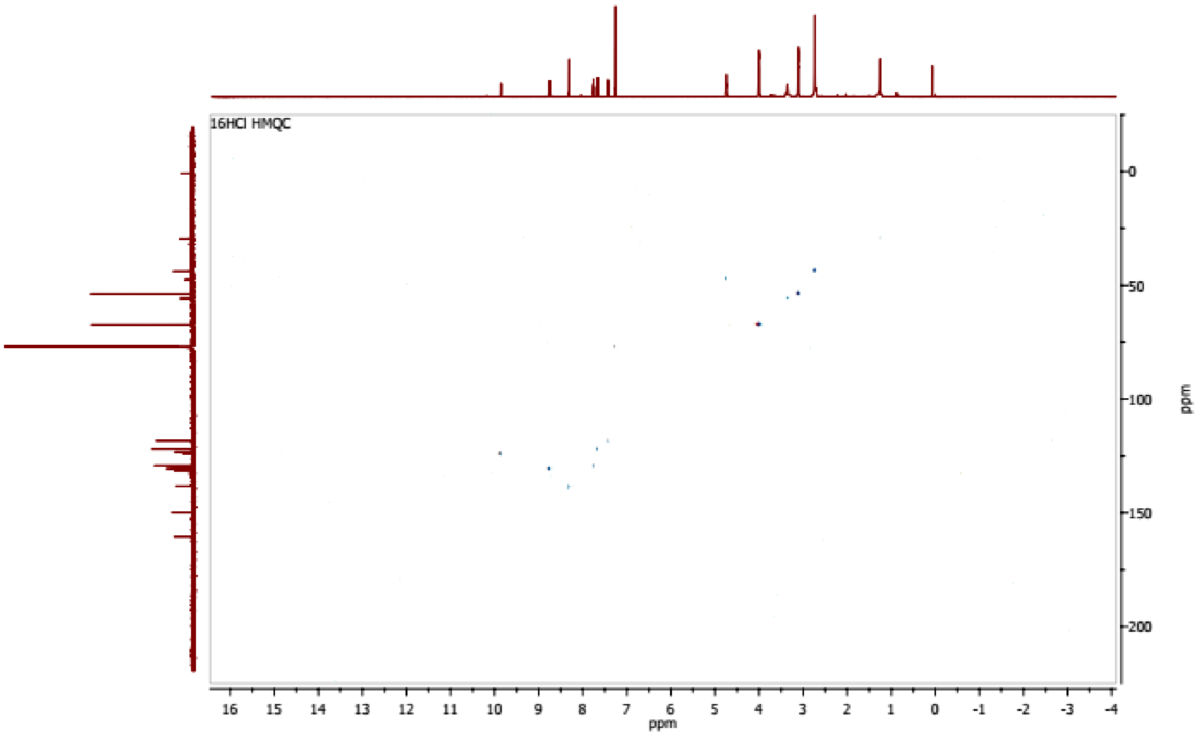

Figure S73. ^{1}H, ^{13}C HMQC NMR spectrum of **16•HCl**.

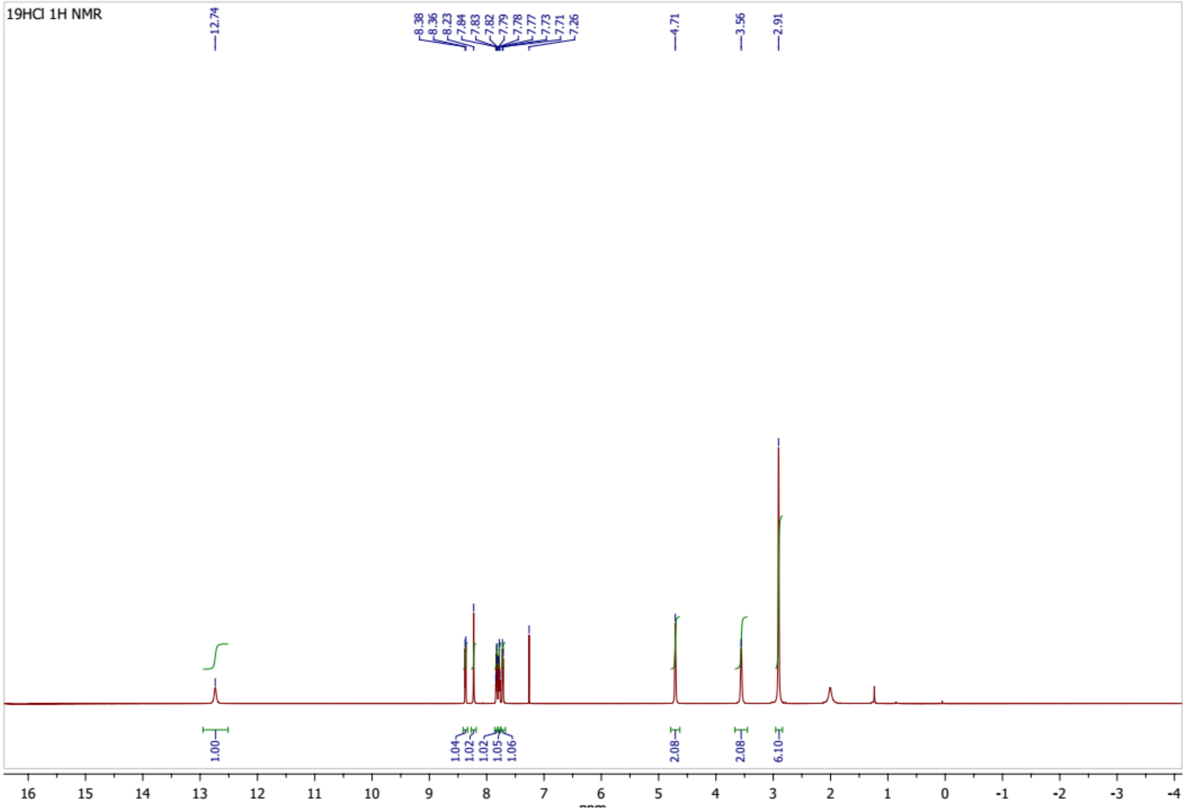

Figure S74. ^{1}H NMR spectrum of **19•HCl**.

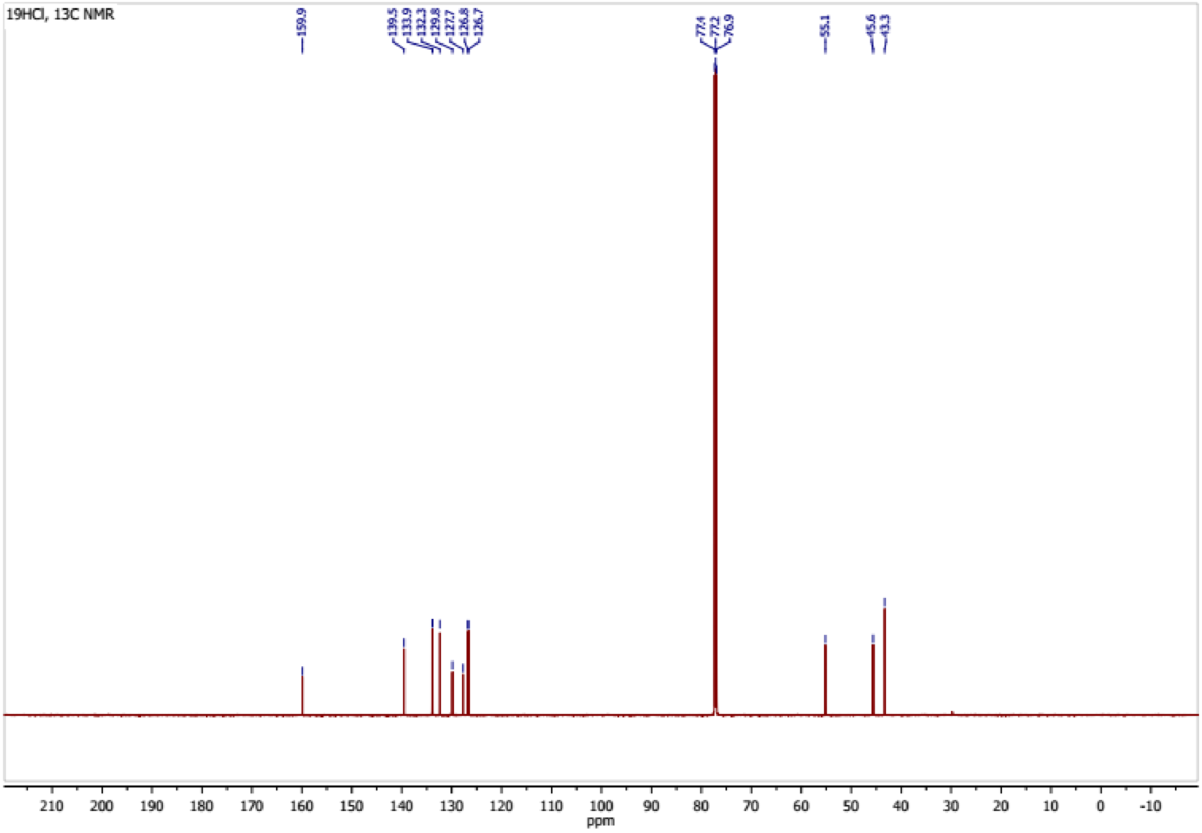

Figure S75. ^{13}C NMR spectrum of **19•HCl**.

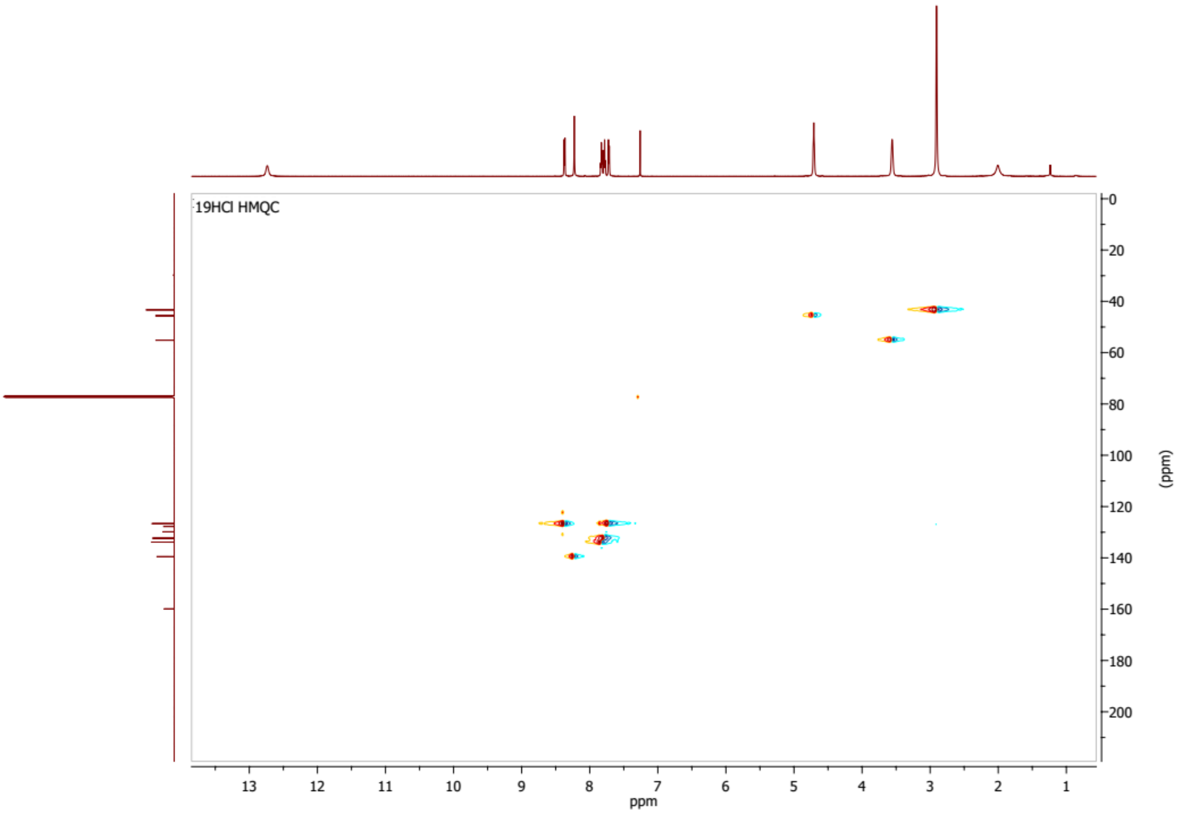

Figure S76. ^{1}H, ^{13}C HMQC NMR spectrum of **19•HCl**.
